# Evaluating the Root Extract of *Reynoutria ciliinervis* (Nakai) Moldenke: An Analysis of Active Constituents, Antioxidant Potential, and Investigation of Hepatoprotective Effects in Rats

**DOI:** 10.3390/molecules29194701

**Published:** 2024-10-04

**Authors:** Zheng Xing, Yang Han, Hao Pang, Li Li, Guangqing Xia, Junyi Zhu, Jing Han, Hao Zang

**Affiliations:** 1 Shenyang Pharmaceutical University, Benxi 117004, China; xingzheng202409@163.com (Z.X.); hy_syphu@163.com (Y.H.); panghao0447@163.com (H.P.); 2 School of Pharmacy and Medicine, Tonghua Normal University, Tonghua 134002, China; lili1984@thnu.edu.cn (L.L.); qingguangx@163.com (G.X.); swx0527@163.com (J.Z.); 3 Key Laboratory of Evaluation and Application of Changbai Mountain Biological Gerplasm Resources of Jilin Province, Tonghua 134002, China

**Keywords:** *Reynoutria ciliinervis* (Nakai) Moldenke, chemical constituents, antioxidant capacity, hepatoprotective ability

## Abstract

*Reynoutria ciliinervis* (Nakai) Moldenke (*R. ciliinervis*) root, a traditional Chinese medicine, was found to exhibit remarkable pharmacological properties through a series of comprehensive investigations. Our study commenced with a qualitative phytochemical analysis that identified 12 bioactive compounds within the plant. Subsequently, utilizing ultraviolet-visible spectrophotometry, the methanol extract emerged as the optimal solvent extract, which was abundant in diverse classes of compounds such as carbohydrates, phenolics, steroids, alkaloids, phenolic acids, and tannins. In vitro antioxidant assays underscored the exceptional free radical scavenging, metal ion chelation, hydrogen peroxide scavenging, singlet oxygen quenching, and *β*-carotene bleaching capabilities of the methanol extract, significantly outperforming other solvent extracts. Further ultra high-performance liquid chromatography–electrospray ionization–quadrupole time of flight–mass spectrometry analysis revealed the presence of 45 compounds, predominantly anthraquinones and phenolics, in the methanol extract. The extract demonstrated robust stability under various conditions, including high temperatures, varying pH levels, and simulated gastrointestinal digestion as well as efficacy in inhibiting the oxidation in edible oils. Acute toxicity tests in mice confirmed the safety of the methanol extract and provided a valuable dosage reference for future studies. Importantly, high-dose methanol extract exhibited a significant pre-protective effect against *D*-galactosamine-induced liver injury in rats, as evidenced by reduced alanine aminotransferase, aspartate aminotransferase, *γ*-glutamyl transpeptidase, malondialdehyde levels, and elevated catalase and albumin levels. These findings suggest a potential role for the methanol extract of *R. ciliinervis* root in treating oxidative stress-related disorders, highlighting the plant’s immense medicinal potential. Our research offers a thorough evaluation of the bioactive components, antioxidant properties, stability, and liver-protecting effects of the methanol extract, setting the stage for deeper investigation and potential clinical applications.

## 1. Introduction

**Figure 1:**
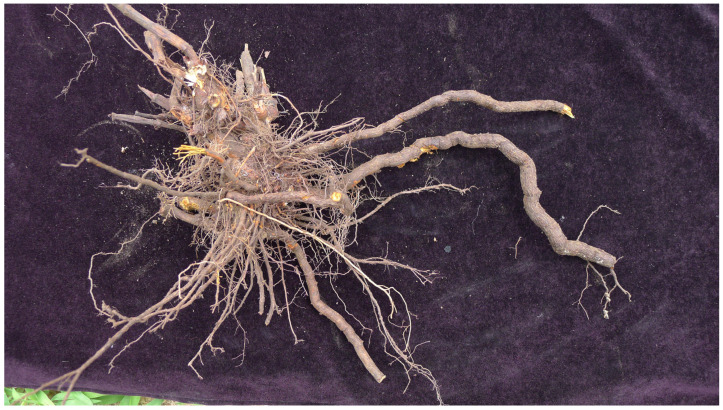
Morphology of *R. ciliinervis* root.

*Reynoutria ciliinervis* (Nakai) Moldenke (*R. ciliinervis*), also known by its two important synonyms *Polygonum ciliinerve* and *Pleuropterus ciliinervis*, is a perennial herb. This versatile plant thrives abundantly in various regions of China, including Shaanxi, Gansu, Sichuan, Guizhou, and Jilin provinces, predominantly inhabiting valley shrubs and crevices of rocky mountain slopes at altitudes spanning from 700 to 1300 m. Its root (Figure 1), renowned for their medicinal properties, exhibits a color and luster strikingly similar to cinnabar, earning it the alias “Zhushaqi.” *R. ciliinervis* root, a celebrated traditional Chinese medicine hailing from the esteemed Taibai Mountain, boasts a rich historical background extending over a millennium. Its medicinal significance and application are first documented in the Song Dynasty’s renowned *Bencao Tujing (Illustrated Classics of Materia Medica)* [1]. Characterized by its cooling nature and bitter taste tinged with a subtle astringency, *R. ciliinervis* harmoniously aligns with the meridians of the stomach, spleen, heart, and liver. This herb possesses a multitude of therapeutic effects, including astringency and acid inhibition, fostering blood circulation to dispel stagnation, arresting bleeding, and facilitating tissue regeneration. Furthermore, it regulates qi to alleviate pain and purges heat and toxins from the body [2]. In the realm of folk medicine, *R. ciliinervis* is extensively employed to address a diverse array of ailments such as epigastric discomfort, diarrhea, swollen sore throats, rheumatic arthralgia, trauma and injuries resulting from falls and blows as well as external trauma with bleeding [3].

*R. ciliinervis* root comprises a diverse array of chemical constituents, primarily anthraquinones, stilbenes, tannins, and polysaccharides, as evidenced by the literature [4]. In recent years, the most extensive research has centered on anthraquinones [5] and polysaccharides [6] within *R. ciliinervis* root. *R. ciliinervis* root boasts numerous pharmacological benefits, including antiviral, antibacterial, anti-inflammatory, antitumor, immunomodulatory, and antioxidant properties, as documented in various studies [7,8,9]. However, the primary research emphasis has been on its anti-inflammatory, antitumor, and antiviral activities [10] with relatively fewer reports focusing on its antioxidant effects. Furthermore, most studies tend to analyze a single phytochemical component in isolation. For instance, one study extracted crude polysaccharides from *R. ciliinervis* root to investigate their antioxidant potential both in vivo and in vitro [5]. Another report examined the impact of *R. ciliinervis* root’s total anthraquinone on the antioxidant capacity of S180 tumor-bearing mice. Additionally, researchers examined thoroughly the regulatory effects of *R. ciliinervis* root’s total anthraquinone on the immune and antioxidant functions of H22 tumor-bearing mice, revealing its significant antioxidant capacity [11,12]. Wang et al. further assessed the free radical scavenging abilities of *R. ciliinervis* root’s total tannin and polysaccharides through a chemical simulation system, emphasizing their potent antioxidant effects [13,14]. While these studies confirm *R. ciliinervis* root’s antioxidant activity, comprehensive and systematic research, encompassing both in vitro and in vivo evaluations, is crucial for its optimal development and utilization.

*R. ciliinervis* root possesses significant application potential and has garnered extensive attention in clinical practice. For instance, there are documented cases where *R. ciliinervis* root, as the primary component, was utilized in self-formulated stomach-protecting capsules to treat 100 patients with chronic atrophic gastritis, achieving a remarkable total effectiveness rate of 98% [15]. Additionally, a study investigating the use of traditional Chinese medicine primarily composed of *R. ciliinervis* root in treating 180 patients with chronic gastritis reported a staggering clinical cure rate of 95.60% [16]. Furthermore, *R. ciliinervis* root tablets were employed in treating 110 patients with acute bacterial dysentery, yielding a total effectiveness rate of 85.45% [17]. Despite these promising clinical outcomes, academic research on *R. ciliinervis* root remains scarce and is in its nascent stages. The present study aims to conduct a comprehensive analysis of *R. ciliinervis* root’s components, both qualitatively and quantitatively, ascertain its antioxidant prowess through in vitro experiments, and identify the extract with the optimal content and strongest antioxidant activity. Utilizing UHPLC-MS technology, the chemical composition of the extract is meticulously characterized, its oxidative stability is rigorously evaluated, and its potential hepatoprotective effects in rats is extensively explored. This endeavor contributes to broadening the application of *R. ciliinervis* root as an antioxidant and propels forward its therapeutic research in oxidative stress-related diseases.

## 2. Results and Discussion

### 2.1. Preliminary Identification of Phytochemicals

Table S1 showcases a qualitative phytochemical analysis of *R. ciliinervis* root, providing valuable insights into its chemical makeup. Notably, the analysis highlights the absence of saponins, alkaloids, and cyanogenic glycosides, emphasizing the exclusion of these potentially bioactive or potentially harmful compounds. However, two captivating findings emerged from the conducted experiments that warrant further scrutiny. Firstly, the detection of flavonoids presented an intriguing paradox with a mix of two positive and two negative results. This inconsistency might stem from the minute concentrations of flavonoids inherent in the plant material. Additionally, the varying sensitivity and detection limits of the utilized analytical methods—including the highly sensitive Shinoda test and lead acetate test, in contrast to the less sensitive AlCl_3_ test and alkaline reagent test—could have contributed to this discrepancy in results. To conclusively resolve this enigma, a quantitative assessment of flavonoid content is paramount. Secondly, the literature consistently cites *R. ciliinervis* root as a copious source of anthraquinones, predominantly present as anthraquinone glycosides, with lower concentrations of free anthraquinones [18]. The experiments were conducted with the existing literature, as all anthraquinone tests yielded positive outcomes, validating their presence in *R. ciliinervis* root. Nevertheless, the specific forms and chemical structures of these anthraquinones remain enigmatic. To unravel these mysteries, the employment of advanced analytical tools, such as UHPLC-MS, is indispensable.

### 2.2. Extraction Yields

It is universally recognized that the selection of solvents utilized in the extraction of chemical compounds from plants plays a pivotal role in determining both the yield and biological potency of the resulting extracts. The strategic use of solvents with varying polarities significantly enhances mass transfer efficiency, expanding the range of extraction capabilities [19]. In this research work, the extraction of bioactive compounds from *R. ciliinervis* root was undertaken utilizing four distinct solvents: water, methanol, ethanol, and an 80% ethanol solution. The primary objective of the study was to characterize the antioxidant activity of these extracts. The experimental findings revealed a diverse range of extraction yields, varying from 21.97 ± 0.09% to 25.50 ± 0.12%, as presented in Table 1. A meticulous comparison of the extraction efficiencies among the solvents emphasized the superiority of water as the solvent of choice, yielding the highest percentage of extract. Notably, the 80% ethanol extract closely followed suit, demonstrating a yield comparable to that of the water extract, highlighting its commendable extraction efficiency. In contrast, the methanol and ethanol extracts exhibited a consistent, albeit slightly lower, yield than the aforementioned solvents, maintaining a relatively high level overall. The exceptional yield of the water extract can be attributed to the abundance of highly polar and water-soluble compounds naturally present in *R. ciliinervis* root, such as carbohydrates and glycosides. However, it is crucial to acknowledge that water extracts may also contain a significant quantity of lesser-studied components, including pigments and pectin, which could potentially complicate the identification and accurate assessment of the biological activities of the targeted compounds. Therefore, while water emerges as an effective solvent for maximizing yield, it is imperative to consider the potential confounding factors when interpreting the results and evaluating the biological significance of the extracted compounds.

### 2.3. Content of Active Ingredients

#### 2.3.1. Total Carbohydrate Content (TCC)

Carbohydrates are the cornerstone of living cell structures, functioning as the primary source of energy essential for sustaining cellular activities and regulating intricate biological processes. Beyond their fundamental energetic role, carbohydrates have garnered substantial attention for their myriad biological activities and potential therapeutic benefits in addressing various diseases. Notably, plant polysaccharides have emerged as a promising frontier in research, exhibiting remarkable pharmacological effects [20,21]. This comprehensive study presents a thorough evaluation of the TCC in diverse extracts. The adopted methodology allows for the simultaneous quantification of both carbohydrates and glycosides, offering a more comprehensive and nuanced analysis [22]. The results, as presented in Table 2, reveal a TCC range extending from 676.51 ± 3.63 to 749.5 ± 7.06 mg glucose equivalents (GE)/g extract, highlighting the substantial variation in the content of carbohydrates and glycosides among different extracts. These findings align with numerous reports that document the isolation of multiple glycosides, primarily anthraquinone glycosides, from *R. ciliinervis* root [23,24]. A meticulous study aimed at optimizing the extraction of anthraquinone from *R. ciliinervis* root has unequivocally demonstrated that 80% ethanol yields the highest total anthraquinone content [25]. This exceptional performance can be attributed to the high polarity of anthraquinone glycosides, which are abundant in *R. ciliinervis* root and are most efficiently extracted using 80% ethanol [26]. Consequently, the TCC of the 80% ethanol extract comprises both carbohydrates and glycosides, whereas the methanol extract primarily contains glycosides.

#### 2.3.2. Total Protein Content (TP_ro_C)

Plant-based proteins are exceedingly beneficial to health, owing to their low fat and cholesterol content, low calorie density, and abundant dietary fiber, making them an invaluable source of nutrition that is readily digested and absorbed by the human body. Furthermore, plant proteins possess antioxidant properties, effectively scavenging free radicals, and they play a pivotal role in maintaining gastrointestinal health and immune regulation [27]. The study has demonstrated that the aqueous extract of *R. ciliinervis* root exhibits a substantial TP_ro_C of 1233.90 ± 14.71 mg bovine serum albumin equivalents (BSAE)/g extract (Table 2). This may initially appear counterintuitive. However, it is crucial to understand that the extract is derived from raw medicinal material and is a concentrated product. The extraction efficiency achieved with water as the solvent is 25.50%, which means that 1 g of the extract is equivalent to 3.922 g of the raw medicinal material. To address the apparent concern of the content exceeding 100%, the calculation has been revised to express the TP_ro_C as 1233.90 mg BSAE per 3.922 g of raw medicinal material. This adjustment ensures accuracy and removes any confusion. Nevertheless, for the sake of brevity and clarity in presenting the experimental results, the extract is typically used as the basis for calculations.

This notable protein content not only has the potential to bolster patients’ overall health but also facilitates the absorption and resolution of inflammatory edema while mitigating pain responses associated with inflammation. Research indicates that the aqueous decoction of *R. ciliinervis* root exhibits significant analgesic, anti-inflammatory, and immunomodulatory effects on mouse ear edema induced by xylene or croton oil [8,28]. This may be attributed to the potent pharmacological effects derived from the high protein content in the decoction, and these findings further support the use of *R. ciliinervis* root in traditional Chinese medicine for treating conditions such as stomach pain, diarrhea, and rectal bleeding.

#### 2.3.3. Total Phenolic Content (TP_he_C)

Plant polyphenols are replete with diverse pharmacological activities and are widely recognized for their myriad of health benefits in humans. These compounds possess a robust antioxidant capacity, which has been demonstrated to exert favorable effects on a wide range of health conditions, including antibacterial and anti-inflammatory properties. They have also shown promise as adjunctive therapies in the management of diseases such as cancer, diabetes, and cardiovascular disorders [29]. As demonstrated by the experimental findings presented in Table 2, the TP_he_C values of various extracts range from 89.18 ± 1.98 to 180.6 ± 1.60 mg gallic acid equivalents (GAE)/g extract. Notably, polyphenols derived from blueberries and grapes have been found to modulate intestinal flora, lower serum cholesterol levels, mitigate insulin resistance, and improve fatty liver degeneration and chronic inflammation [30,31]. Experimental data further suggest that solvents containing alcohol are more effective in extracting polyphenols. Specifically, the TP_he_C values of ethanol or 80% ethanol extracts are approximately twice those of water extracts, indicating that the inclusion of a certain proportion of ethanol during the extraction process can significantly enhance the yield and thus the potency of polyphenols.

#### 2.3.4. Total Steroid Content (TSC)

Steroids, naturally abundant compounds, display a myriad of biological activities such as anti-inflammatory, antitumor, hormone-regulating, and immunosuppressive properties [32]. A comprehensive analysis was undertaken to assess the total steroidal content (TSC) present in four distinct solvent extracts of *R. ciliinervis* root. Experimental outcomes revealed notable variations in TSC among the tested solvent extracts (Table 2). Specifically, the methanol extract exhibited the highest TSC, reaching 3.44 ± 0.08 mg oleic acid equivalents (OAE)/g extract. Furthermore, the literature review illuminated that only a single steroid compound, *β*-sitosterol, has been isolated from *R. ciliinervis* root [10]. This limited repertoire of steroidal compounds in *R. ciliinervis* root provides a plausible explanation for the relatively low TSC levels observed in the study.

#### 2.3.5. Total Alkaloid Content (TAC)

Alkaloids, also referred to as pseudo-bases due to their properties resembling those of alkalis, are ubiquitous in the plant kingdom and are a significant component of Chinese herbal medicine, which are known for their nitrogen-containing structure and potent bioactivity [33,34]. In the analysis presented in Table 2, notably, the water extract yielded the highest concentration of alkaloids, which was measured at 12.34 ± 0.89 mg berberine hydrochloride equivalents (BHE)/g extract. It is noteworthy that only two specific alkaloids: pyrrolezanthine-6-methyl-ether and *N*-trans-feruloyl-3-methoxytyramine—have been reported in the literature pertaining to the plant [35,36,37]. This finding not only validates the accuracy and precision of our experimental methodology but also emphasizes the fact that *R. ciliinervis* root contains alkaloids in relatively low concentrations.

#### 2.3.6. Total Flavonoid Content (TFC)

Flavonoids, a versatile class of natural polyphenols, are prevalent in diets, and they are abundantly found in vegetables, fruits, grains, and even beverages such as tea. They serve as essential dietary components for humans, providing numerous health benefits [38]. The remarkable versatility of flavonoids extends far beyond their nutritional value, as they are also incorporated into health supplements, pharmaceutical formulations, and cosmetic products. Their diverse pharmacological effects, which encompass antibacterial, antioxidant, and anti-inflammatory properties, play a significant role in mitigating the risk of various diseases [39,40,41]. Experimental evaluations have yielded valuable insights into the flavonoid content of various extracts. Specifically, the TFC in these extracts ranged from 2.73 ± 0.16 to 10.84 ± 0.54 mg quercetin equivalents (QE)/g of extract, highlighting the substantial variation among different sources.

Free flavonoids, devoid of sugar moieties, generally tend to be insoluble in water but readily dissolve in organic solvents like methanol and ethanol. In contrast, flavonoid glycosides, which are flavonoids bound to sugar molecules, display enhanced solubility in solvents such as hot water, methanol, and ethanol. Upon perusing the current scientific literature, it becomes apparent that only a limited number of flavonoids—specifically, kaempferirin, annulatin 3′-*O*-*β*-*D*-xyloside [35,36], quercetin, and quercetin-3-*O*-glucoside [10], have been isolated from *R. ciliinervis* root. Based on the qualitative analysis results, the initial hypothesis regarding the low flavonoid content in *R. ciliinervis* root has been validated.

#### 2.3.7. Total Phenolic Acid Content (TPAC)

This comprehensive study extends beyond the examination of TP_he_C to encompass an analysis of TPAC with a focus on diverse extracts sourced from *R. ciliinervis* root. The findings, as detailed in Table 2, reveal a considerable variation in TPAC values, spanning from 3.71 ± 0.23 to 11.05 ± 0.64 mg caffeic acid equivalents (CAE)/g extract. To emphasize the significance of the findings, it is imperative to highlight that the experimental data conclusively verify the presence of phenolic acids in *R. ciliinervis* root, albeit in relatively low concentrations.

#### 2.3.8. Total Tannin Content (TT_an_C), Gallotannin Content (GC) and Condensed Tannin Content (CTC)

Tannins, inherently occurring polyphenol compounds found in plants, serve as vital secondary metabolites that bolster the chemical defense mechanisms of plants. They effectively shield plants from the threats posed by pathogens, insects, herbivores, and other potential adversaries. Additionally, tannins play a pivotal regulatory role in the growth and development of plants. Tannins exhibit a broad spectrum of pharmacological effects, and they are renowned for their antioxidant, antibacterial, anthelmintic, antiviral, and anti-inflammatory properties. Broadly speaking, tannins can be categorized into two main groups: hydrolyzable tannins and condensed tannins, with gallotannins being a notable representative of the former category [42,43,44]. To gain a deeper understanding of the tannin content in various extracts of *R. ciliinervis* root, measurements were conducted for GC, CTC, and TT_an_C, as outlined in Table 2. Condensed tannins, for instance, display limited solubility in polar organic solvents, whereas methanol or ethanol are commonly used solvents for the extraction of hydrolyzable tannins [45,46]. This explains the higher CTC content observed in the water extract. However, for the extraction of total tannins, the use of alcohol-based solvents is advantageous, ensuring a more efficient extraction process.

This study highlights the immense potential of *R. ciliinervis* root as a repository of bioactive compounds, such as proteins, carbohydrates, glycosides, and phenolics, each endowed with distinct pharmacological properties. The literature attests to *R. ciliinervis* root’s abundance in anthraquinones, tannins, and polysaccharides [4]. Notably, the anthraquinone content in *R. ciliinervis* root extracts has been reported to reach 80.81%, primarily in the form of bound anthraquinones [47]. Furthermore, the polysaccharide extraction rate from *R. ciliinervis* root purification has achieved 5.1% [6], while an ultrasound-assisted extraction method yielded a 2.0% tannin extraction rate [48]. To date, an impressive 86 compounds have been isolated and identified from *R. ciliinervis* root, encompassing anthraquinones, stilbenes, flavonoids, organic acids, lignans, and alkaloids [49], which confirms the accuracy of the content determination outcomes. Notably, our research has discovered that extraction with methanol, ethanol, and 80% ethanol yields higher concentrations of active ingredients compared to water extraction. It is crucial to emphasize that numerous qualitative analyses encompass chemical reactions that transcend the realm of any specific component type. These reactions often involve intricate interactions among various molecular entities, making them non-exclusive to any single component being studied. To ensure the authenticity of the results, at least two distinct experimental protocols were devised, minimizing the risk of false positives and enhancing the precision of the detection outcomes. Concurrently, these outcomes not only offer guidance but also provide robust support for quantitative analysis endeavors.

### 2.4. Antioxidant Activity In Vitro

#### 2.4.1. 1,1-Diphenyl-2-Picrylhydrazyl Radical (DPPH) and 2,2′-Azino-Bis(3-Ethylbenzothiazoline-6-Sulfonicacid) Diammonium Salt (ABTS)

Evaluating the antioxidant potency of natural compounds and their extracts through their free radical scavenging capacity is crucial for understanding their therapeutic potential. DPPH and ABTS, two stable free radicals, serve as reliable benchmarks in assessing such capacity, as they undergo reduction and decolorization upon encountering antioxidants. This process is quantitatively measured by assessing the resultant absorbance, which inversely correlates with the antioxidant’s effectiveness [19]. In the DPPH assay, all extracts except the water extract demonstrated superior scavenging capabilities, surpassing the performance of the antioxidant butylated hydroxytoluene (BHT) and even approaching the efficacy of trolox and *L*-ascorbic acid (Table 3). These findings align with previous research identifying active components in *R. ciliinervis* root capable of neutralizing DPPH radicals [6,23,37,50], strengthening the observed results. Similar results were also observed in the ABTS assay (Table 3). Interestingly, studies on a closely related plant species, *Polygonum multiflorum*, have also documented significant ABTS free radical scavenging activity, particularly in its roots, highlighting the prevalence of potent antioxidants within this genus [51]. It is hypothesized that the robust free radical scavenging abilities of *R. ciliinervis* root may stem from its rich abundance of anthraquinones, their glycosides, and phenolic compounds.

#### 2.4.2. Hydroxyl Radicals and Superoxide Radicals

Evaluating the antioxidant capacity of a compound or plant extract necessitates a thorough examination of the hydroxyl and superoxide radicals generated within the biological system. These radicals, particularly hydroxyl radicals, possess highly reactive chemical properties with hydroxyl radicals exhibiting the most potent reactivity among them. An excess of these radicals in the human body can inflict oxidative damage on vital biomolecules, including proteins and DNA, ultimately disrupting the normal cellular structure and function. This, in turn, accelerates the aging process and predisposes individuals to a myriad of diseases. Consequently, the capacity to neutralize these free radicals serves as a crucial indicator of antioxidant detoxification, effectively mitigating cellular oxidative toxicity [13,52].

Our comprehensive investigation revealed that with the exception of the aqueous extract, all three tested extracts demonstrated remarkable hydroxyl radical scavenging abilities (Table 3). When discussing superoxide radicals, notably, the scavenging abilities of both 80% ethanol and ethanol extracts exceeded those of curcumin by a factor of ten (Table 3). This superior performance might be attributed to the rich content of tannins and polysaccharides present in the extracts, as prior research has emphasized the potent hydroxyl and superoxide radical scavenging abilities of these compounds in *R. ciliinervis* root [6,12,13,50].

#### 2.4.3. Ferric Reducing Antioxidant Power (FRAP) and Cupric Reducing Antioxidant Capacity (CUPRAC)

The capacity of a sample to reduce iron and copper ions serves as an indicator of its antioxidant potential [53]. Two prevalent methods employed for this assessment are the FRAP and CUPRAC assays. Experimental findings from the FRAP assay reveal a pronounced positive correlation with the outcomes of the DPPH test. In contrast, during the CUPRAC test, the extracts, barring the water extract, manifested copper ion reduction capabilities that closely resembled that of trolox (Table 4). Furthermore, there is a documented report stating that *Polygonum multiflorum* demonstrates remarkable reduction abilities in FRAP experiments [51]. Additionally, some studies in the literature emphasize the iron ion reduction potential of *R. ciliinervis* root extracts [13], which is primarily attributed to the abundant phenol content present in both plant species.

#### 2.4.4. Metal Chelation

The presence of ferrous and copper ions can amplify the Fenton reaction, resulting in heightened oxidative stress in the body [54]. Consequently, assessing the metal chelating capacity of samples is paramount for evaluating their antioxidant potential. Experimental findings revealed that various extracts demonstrated varying degrees of iron-chelating abilities. In terms of copper ion chelation, all extracts displayed commendable chelating abilities (Table 4). These outcomes emphasize the inferior metal chelating ability of the water extract in contrast to the superior metal ion chelating proficiency of the methanol extract. Furthermore, it was discovered that both *Polygonum minus* and *Polygonum glabrum* extracts possess chelating effects, which are intimately tied to the presence of phenolic compounds [19,55].

#### 2.4.5. Hydrogen Peroxide (H_2_O_2_)

H_2_O_2_ is a potent oxidant that arises as a by-product during the body’s metabolic processes. While it plays a crucial role in cell signaling and immune responses, excessive levels of H_2_O_2_ can trigger oxidative stress, resulting in an elevation of intracellular reactive oxygen species (ROS) and subsequent damage to mitochondria, ultimately leading to apoptosis [56]. Consequently, the effective scavenging of H_2_O_2_ is paramount for preserving good health. Experimental findings have demonstrated that various extracts of *R. ciliinervis* root exhibit robust H_2_O_2_ scavenging capabilities (Table 5). Furthermore, research has indicated that the H_2_O_2_ scavenging activity of the methanol extract of *Polygonum glabrum* is comparable to that of vitamin E [55].

#### 2.4.6. Singlet Oxygen

Singlet oxygen, a highly reactive variant of molecular oxygen, is generated within mammalian cells under pathophysiological circumstances, posing a threat to health by oxidizing vital cellular macromolecules such as lipids, nucleic acids, and proteins [57]. In the singlet oxygen scavenging experiment conducted, all extracts except for the water extract exhibited commendable scavenging activity (Table 5).

#### 2.4.7. β-Carotene Bleaching

*β*-Carotene is a natural pigment endowed with crucial physiological functions and biological activities. It is prevalent in plants but prone to fading upon oxidation [58]. In this experiment, linoleic acid was subjected to heating and oxidation, generating free radicals that subsequently caused the bleaching of *β*-carotene. However, in the presence of antioxidants, the formation of these free radicals was delayed, mitigating the bleaching rate of *β*-carotene. The antioxidant activity was quantitatively assessed by monitoring the decrease in *β*-carotene’s absorbance over time [59].

**Figure 2:**
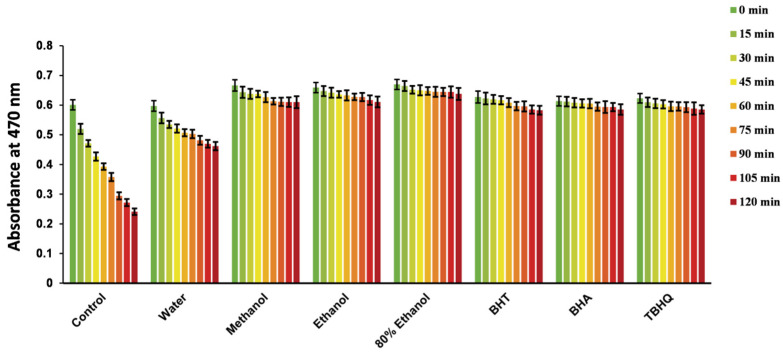
Alterations in the absorbance of *β*-carotene in the presence of different extracts of *R. ciliinervis* root. BHT, BHA and TBHQ were employed as positive controls. BHT: butylated hydroxytoluene; BHA: butyl hydroxyanisole; TBHQ: tertiary butylhydroquinone.

This study employed the *β*-carotene bleaching assay to evaluate the antioxidant potential of various extracts of *R. ciliinervis* root. Notably, the methanol, ethanol, and 80% ethanol extracts demonstrated robust antioxidant capabilities (Table 5), surpassing synthetic antioxidants like BHT, butyl hydroxyanisole, and tertiary butylhydroquinone (TBHQ) (Figure 2). Additionally, existing literature attests to the *β*-carotene bleaching inhibition activity exhibited by *Polygonum minus*, further reinforcing the significant potential of the *Polygonum* genus as natural antioxidants for food and pharmaceutical applications [60,61].

The objective of this study is to investigate the antioxidant potential of *R. ciliinervis*. The research findings reveal that the plant contains active constituents, representing valuable natural sources of antioxidants. Notably, the methanol extract displays particularly promising outcomes, demonstrating superior values in TCC, TP_he_C, TAC, TPAC, TT_an_C, and GC. Furthermore, this extract exhibits robust scavenging capabilities against four distinct free radicals, strong reduction abilities toward iron and copper ions, moderate metal ion chelation, effective scavenging of singlet oxygen and H_2_O_2_, and it even surpasses synthetic antioxidants in inhibiting *β*-carotene bleaching. A previous report examining the antioxidant capacities of various solvent extracts from *Polygonum minus* concurred that the methanol extract possessed the highest antioxidant capacity [19], aligning with the research findings. Consequently, guided by these experimental results, the methanol extract was selected for further in-depth investigation, utilizing UHPLC-MS for compound identification with the aim of elucidating its active ingredients. Subsequently, the stability of the methanol extract under various environmental conditions will be evaluated with a focus on its ability to inhibit edible oil oxidation. This assessment aims to determine the stability and sustainability of its antioxidant capacity across various environments and conditions. Following this, acute toxicity tests were performed on mice to evaluate the extract’s acute toxicity and establish dosage guidelines for subsequent rat liver protection experiments. Ultimately, the focus shifted to examining the extract’s pre-protective effects against acute liver injury in rats with the ultimate goal of uncovering its therapeutic potential and protective mechanisms.

### 2.5. UHPLC-MS Identification

The current study examines the chemical composition of the methanol extract of *R. ciliinervis* root, utilizing UHPLC-ESI-Q-TOF-MS technology. Through meticulous comparison of molecular and fragment ions with reference data, the sources of the reference data are derived from the published literature (please refer to the analysis process of each peak for further details) and reputable public databases, including the Human Metabolome Database (https://hmdb.ca/, accessed on 10 August 2024) and MassBank (https://www.massbank.jp/, accessed on 10 August 2024). A comprehensive list of 45 bioactive compounds has been compiled (Table 6) with their structural representations illustrated in Figure S1. Additionally, Figure S2 presents the UHPLC-MS findings obtained in positive-ion mode, while detailed MS and MS/MS spectra are available in the Supplementary Materials (Figures S3–S100).

This thorough chemical characterization of the methanol extract provides a rich understanding of the diverse array of compounds it encompasses. Notably, several of the identified compounds have been previously isolated from *R. ciliinervis* root and have been implicated in its antioxidant properties. These findings emphasize the significant contribution of these compounds to the overall therapeutic potential of *R. ciliinervis* root, serving as a robust foundation for future research endeavors. By focusing on specific compounds responsible for the observed antioxidant effects, this study provides a foundation for targeted therapeutic developments and advancements in medicinal applications, setting the stage for future progress.

Peak 1 was identified as *D*-sucrose by matching fragment ions (*m*/*z* 343.1175 [M+H]^+^, 180.0820 [M−C_6_H_11_O_5_+H]^+^, and 163.0560 [M−C_6_H_11_O_6_]^+^) with reference [62]. Peak 2 at *m*/*z* 222.1066 had MS^2^ ions at *m*/*z* 163.0339 [M−C_2_H_3_N_2_O_2_+NH_4_]^+^ and 91.0519 [M−C_4_H_7_N_2_O_3_+NH_4_]^+^, and it was tentatively assigned as glutamylglycine [63]. The mass spectrum of peak 3 displayed an ion at *m*/*z* 292.1318 with its MS^2^ spectrum showing fragments at *m*/*z* 153.0145 and 136.0722. Peak 4 registered an *m*/*z* of 171.0241 and exhibited fragment ions at *m*/*z* 132.0983 [M−OH+H]^+^ and 103.0515 [M−COOH]^+^, and it was presumptively identified as citramalic acid [64]. Peak 5 displayed a [M+H]^+^ ion at *m*/*z* 307.0742 with its main fragment ions at *m*/*z* 289.0641 [M−OH]^+^ and 181.0462 [M−C_6_H_5_O_3_]^+^. These findings were characteristic of epigallocatechin [65] or gallocatechin [66]. Peak 6 (*m*/*z* 188.0657) was recognized as phenylalanine, which was based on the typical fragment ions at *m*/*z* 165.0507 [M]^+^ and 100.0730 [M−C_3_H_6_NO_2_+Na]^+^ [67]. Peak 7 had an *m*/*z* of 579.1385 and gave fragment ions at *m*/*z* 453.1047 and 289.0649, which correlated with the loss of −C_6_H_5_O_2_−OH+H and −C_15_H_13_O_6_, suggesting peak 7 was procyanidin B1 [68]. The precursor ion [M+H]^+^ of peak 8 appeared at *m*/*z* 291.0798, with its main fragment ions at *m*/*z* 273.0710 [M−OH]^+^ and 165.0512 [M−OH−C_6_H_5_O_2_]^+^, corresponding to *D*-(+)-catechin or (−)-epicatechin [10]. Peak 9 at *m*/*z* 365.0759 had MS^2^ ions at *m*/*z* 297.0541 [M−COOH]^+^ and 181.0453 [M−C_6_H_11_O_6_+NH_4_]^+^, and it was identified as caffeic acid 3-glucoside [69]. The main fragment ions of peak 10 (*m*/*z* 395.1254) were at *m*/*z* 227.1697 [M−C_6_H_11_O_5_+NH_4_]^+^ and 194.1131 [M−C_6_H_11_O_6_]^+^, and it was assigned as *β*-syringin [70].

Peak 11 had an *m*/*z* of 439.1509 and MS^2^ ions at *m*/*z* 340.2528 [M−C_6_H_5_+H]^+^ and 322.2425 [M−C_6_H_5_−H_2_O+H]^+^, and it was identified as 2-phenylethyl 6-*O*-*β*-*D*-xylopyranosyl-*β*-*D*-glucopyranoside [71]. Peak 12 showed an [M+NH_4_]^+^ ion at *m*/*z* 654.1180, producing major fragment ions at *m*/*z* 467.0745 [M−C_7_H_5_O_5_]^+^ and 449.0634 [M−C_7_H_5_O_5_−H_2_O]^+^, which is characteristic of 1,3,6-trigalloyl glucose [72]. Peak 13 was identified as *N*-acetylmethionine with an *m*/*z* of 191.0653. The MS^2^ spectrum displayed connections at *m*/*z* 177.0044 and 133.0623, corresponding to the loss of −CH_3_+H and −C_2_H_4_NO [73]. Peak 14, with an *m*/*z* of 245.0748, was identified as uridine, featuring fragment ions at *m*/*z* 227.1707 and 133.0612, corresponding to the loss of −OH and −C_4_H_3_N_2_O_2_ [74]. The mass spectrum of peak 15 exhibited an ion at *m*/*z* 463.1131, with its MS^2^ spectrum showing fragments at *m*/*z* 340.2531 [M−C_7_H_7_O_2_+H]^+^, 283.0540 [M−C_6_H_11_O_6_]^+^, and 163.0340 [M−C_16_H_11_O_6_]^+^, and it was recognized as chrysoeriol 7-*O*-glucoside [75]. Peak 16 was observed at *m*/*z* 600.2537 and characterized as (+)-lyoniresinol-3a-*O*-*β*-glucoside. Its MS^2^ ions at *m*/*z* 340.2528 and 163.0143 indicated the loss of −C_8_H_9_O_3_+H and −C_22_H_27_O_8_, respectively [76]. Peak 17 had an [M+H]^+^ ion at *m*/*z* 391.1310, producing major fragment ions at *m*/*z* 211.0708 [M−C_6_H_11_O_6_]^+^ and 107.0461 [M−C_13_H_16_O_7_+H]^+^, which are characteristics of polydatin [37]. Peak 18 was detected at *m*/*z* 419.1234 with fragment ions at *m*/*z* 257.0745 [M−C_6_H_11_O_6_+NH_4_]^+^ and 163.0150 [M−C_15_H_11_O_4_]^+^, and it was assigned as polygonimitin B [37]. Peak 19 (*m*/*z* 419.1248) was recognized as liquiritin based on the typical fragment ions at *m*/*z* 257.0747 [M−C_6_H_11_O_6_+NH_4_]^+^ and 222.1077 [M−C_6_H_11_O_6_−OH]^+^ [77]. The mass spectrum of peak 20 revealed an ion at *m*/*z* 183.0729 with its MS^2^ spectrum showing fragments at *m*/*z* 133.0826 and 89.0570.

Peak 21 had an *m*/*z* of 229.0796 with MS^2^ ions at *m*/*z* 229.0810 [M−OH+NH_4_]^+^ and 127.0123 [M−C_8_H_7_O+NH_4_]^+^, and it was tentatively identified as resveratrol based on the literature [37]. Peak 22, with an *m*/*z* of 233.0745 and MS^2^ ions at *m*/*z* 215.0650 [M−OH]^+^ and 205.0547 [M−CO+H]^+^, was tentatively characterized as cassiachromone [37]. Peak 23 at *m*/*z* 455.0837 was proposed as emodin 8-*β*-*D*-glucopyranoside with the main MS^2^ ions at *m*/*z* 271.0545 and 197.0547 corresponding to the loss of −C_9_H_6_O_3_+H and −C_15_H_9_O_4_+NH_4_ [37]. Peak 24 had an *m*/*z* of 543.1372 with major fragments including *m*/*z* 245.1807 [M−C_13_H_15_O_9_+NH_4_]^+^ and 192.1337 [M−C_20_H_21_O_7_+Na]^+^, and it was identified as resveratrol-3-*O*-(2″-*O*-galloyl)-*β*-*D*-glucopyranoside [24]. Peak 25 exhibited a precursor ion [M+H]^+^ at *m*/*z* 409.1396, generating major fragment ions at *m*/*z* 431.1235 [M+Na]^+^, 391.1313 [M−OH]^+^, and 229.0809 [M−C_6_H_11_O_6_]^+^, corresponding to torachrysone 8-*O*-glucoside [37]. Peak 26 had an *m*/*z* of 271.0528 with MS^2^ ions at *m*/*z* 225.0495 [M−CO−OH]^+^ and 197.0546 [M−C_5_H_4_O_2_+Na]^+^, and it was identified as aloe emodin [78]. The mass spectrum of peak 27 displayed an ion at *m*/*z* 309.0892 with its MS^2^ spectrum showing fragments at *m*/*z* 291.0800 and 147.0401. Peak 28 showed an *m*/*z* of 177.0495 and was recognized as 7-methoxycoumarin based on the characteristic fragment ions at *m*/*z* 149.0557 [M−CO+H]^+^, 145.0244 [M−OCH_3_]^+^, and 117.0301 [M−OCH_3_−CO]^+^ [79]. Peak 29, with an *m*/*z* of 256.0662, was identified as piscidic acid based on the characteristic fragment ions at *m*/*z* 222.1073 [M−2OH]^+^ and 194.1128 [M−OH−COOH]^+^ [35]. Peak 30 had [M+H]^+^ at *m*/*z* 271.0531 and produced significant fragment ions at *m*/*z* 163.0348 [M−C_6_H_4_O_2_+H]^+^ and 107.0464 [M−C_8_H_4_O_4_+H]^+^, which are characteristic of emodin [37].

Peak 31 presented an *m*/*z* of 285.0684 with fragment ions at *m*/*z* 201.0858 and 143.0363 after the elimination of −C_4_H_4_O_2_+H and −C_8_H_4_O_4_+Na, leading to the identification of 1-methyl emodin [37]. Peak 32 appeared at *m*/*z* 285.0686 with MS^2^ ions at *m*/*z* 242.0522 [M−CO−CH_3_+H]^+^ and 222.1077 [M−CH_3_−OH−OCH_3_+H]^+^, and it was identified as emodin 3-methyl ether [37]. Peak 33, observed at *m*/*z* 256.0666, was characterized as emodin anthrone with its MS^2^ ions at *m*/*z* 224.1228 and 194.1128 indicating the loss of −OH−CH_3_ and −2OH−CO, respectively [80]. Peak 34 at *m*/*z* 511.1279 exhibited fragment ions at *m*/*z* 453.3350 and 205.0554, indicating losses of −OH−H_2_O and −C_16_H_11_O_5_, and it was thus determined to be physicon-8-*β*-*D*-(6′-*O*-acetyl)glucoside [23]. The mass spectrum of peak 35 displayed an ion at *m*/*z* 285.0686 with its MS^2^ spectrum showing fragments at *m*/*z* 270.0827 and 222.1075. Peak 36, a [M+H]^+^ ion at *m*/*z* 521.1683, was suggested to be piceid-2″-*O*-conmarate, with its MS^2^ ions at *m*/*z* 293.0960 [M−C_14_H_11_O_2_−OH+H]^+^ and 275.0852 [M−C_14_H_11_O_2_−OH−H_2_O+H]^+^ corresponding to the literature [24]. Peak 37 was identified as palmitic acid at *m*/*z* 274.2673, with characteristic fragment ions at *m*/*z* 212.2320 [M−COOH+H]^+^, 71.0831 [C_5_H_11_]^+^, and 57.0679 [C_4_H_9_]^+^ [81]. Peak 38 at *m*/*z* 318.2925, with MS^2^ ions at *m*/*z* 183.0735 [C_13_H_27_]^+^, 155.0428 [C_11_H_23_]^+^, and 127.0123 [C_9_H_19_]^+^, was tentatively identified as phytosphingosine based on the literature [82]. Peak 39 displayed [M+NH_4_]^+^ at *m*/*z* 298.2663 and produced significant fragment ions at *m*/*z* 235.1866 [M−COOH]^+^ and 222.1074 [C_16_H_29_+H]^+^, which is characteristic of linoleic acid [83]. The mass spectrum of peak 40 displayed an ion at *m*/*z* 415.2021 with its MS^2^ spectrum showing fragments at *m*/*z* 340.2524 and 183.0737.

Peak 41 had [M+H]^+^ at *m*/*z* 302.2980 and yielded significant fragment ions at *m*/*z* 276.1902 [M−C_3_H_7_+NH_4_]^+^, 183.0741 [C_13_H_27_]^+^, and 100.0730 [C_7_H_15_+H]^+^, which are characteristic of sphinganine [84]. The mass spectrum of peak 42 displayed an ion at *m*/*z* 280.2560 with its MS^2^ spectrum showing fragments at *m*/*z* 263.2314 and 222.1071. Peak 43 exhibited a [M+NH_4_]^+^ peak at *m*/*z* 294.2350, which produced primary fragment ions at *m*/*z* 277.2105 [M−OH+NH_4_]^+^ and 249.2150 [M−COOH+NH_4_]^+^. These fragment ions are indicative of stearidonic acid [85]. The precursor ion [M+NH_4_]^+^ of peak 44 appeared at *m*/*z* 320.2480 with its main fragment ions at *m*/*z* 280.2576 [M−COOH+Na]^+^, 135.0404 [C_10_H_15_]^+^, and 123.0885 [C_9_H_14_+H]^+^, corresponding to eicosapentanoic acid [86]. Peak 45 registered an *m*/*z* of 296.2528 and exhibited fragment ions at *m*/*z* 279.2264 [M+H]^+^, 149.1033 [C_11_H_17_]^+^, and 135.0404 [C_10_H_15_]^+^, and it was presumptively identified as *α*-linolenic acid [87]. Peak 46 appeared at *m*/*z* 279.2245 with MS^2^ ions at *m*/*z* 222.1077 [M−C_4_H_9_+Na]^+^, 183.0734 [C_13_H_27_]^+^, and 127.0123 [C_9_H_19_]^+^, and it was identified as 14-methylpentadecanoic acid [88]. Peak 47 displayed [M+NH_4_]^+^ at *m*/*z* 471.3196 and produced significant fragment ions at *m*/*z* 155.0432 [C_3_H_9_NO_4_P+H]^+^ and 140.1148 [C_2_H_7_NO_4_P]^+^, which is characteristic of 1-palmitoyl-2-hydroxy-sn-glycero-3-PE [89]. The mass spectrum of peak 48 exhibited an ion at *m*/*z* 338.3331, with its MS^2^ spectrum showing fragments at *m*/*z* 279.1536 [C_20_H_39_]^+^, 237.2582 [C_17_H_33_]^+^, and 149.0196 [M−C_13_H_25_NO+Na]^+^, and it was recognized as erucamide [90]. Peak 49 presented an *m*/*z* of 437.3736 with fragment ions at *m*/*z* 407.3290 and 338.3350 after the elimination of −CH_2_OH+Na and −C_7_H_15_+Na, leading to the identification of *β*-sitosterol [10].

### 2.6. Stability of Methanol Extract

**Figure 3:**
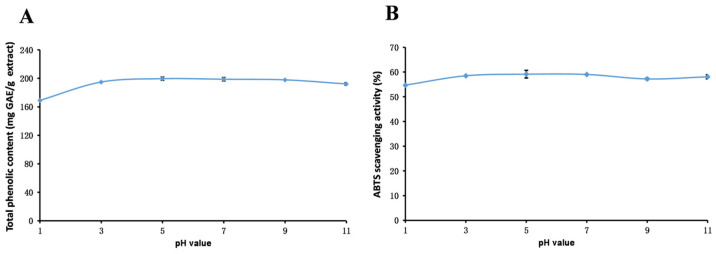
Total phenolic content (**A**) and ABTS (**B**) assays to assess the stability of the methanol extract of *R. ciliinervis* root at various pH values. ABTS: 2,2′-azino-*bis*(3-ethylbenzothiazoline-6-sulfonicacid) diammonium salt.

**Figure 4:**
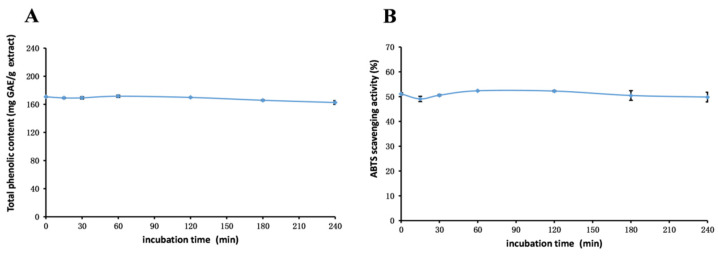
Total phenolic content (**A**) and ABTS (**B**) assays to assess the thermal stability of the methanol extract of *R. ciliinervis* root. ABTS: 2,2′-azino-*bis*(3-ethylbenzothiazoline-6-sulfonicacid) diammonium salt.

**Figure 5:**
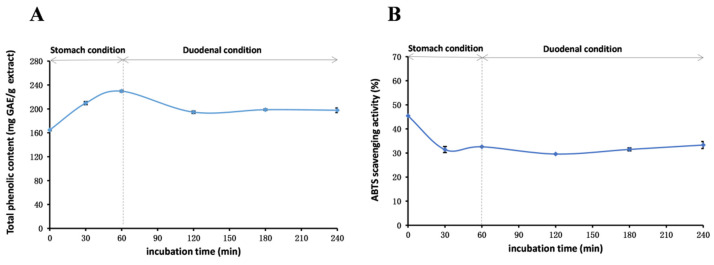
Total phenolic content (**A**) and ABTS (**B**) assays to assess the stability of the methanol extract of *R. ciliinervis* root an in vitro simulation of the human digestive system. ABTS: 2,2′-Azino-*bis*(3-ethylbenzothiazoline-6-sulfonicacid) diammonium salt.

This study aims to comprehensively investigate the stability and antioxidant properties of phenolics present in *R. ciliinervis* root methanol extract. The results presented in Figure 3, Figure 4 and Figure 5 offer significant insights into the stability of the methanol extract under varying conditions, adding substantial value to the exploration of the properties.

#### 2.6.1. pH Stability

As depicted in Figure 3, the scavenging capacity of total phenolics and ABTS radicals in the methanol extract exhibits a similar fluctuation pattern under varying pH conditions. With an increase in pH, both parameters initially rise and then decline, peaking at pH 5. This trend is likely attributed to the hydrolysis of bound phenolics under these conditions, leading to an augmentation in total phenolic content and an enhancement in the ABTS radical scavenging capability. However, as the pH further increases, the total phenolic content diminishes, which is potentially due to the degradation of certain polyphenolic compounds under alkaline conditions [91]. These findings highlight the significant influence of pH on the stability of phenolic compounds in *R. ciliinervis* root, and it is advisable to store the methanol extract of *R. ciliinervis* root in neutral or slightly acidic environments.

#### 2.6.2. Thermal Stability

Upon investigating the impact of heating time on the methanol extract, as illustrated in Figure 4, it was discernible that the TP_he_C value and ABTS radical scavenging activity initially decreased at 30 min, which was followed by an increase to their peak values when the heating time was extended to 60 min. Subsequently, no notable alterations were observed with further heating. The findings indicate that heating time exerts a minimal influence on the TP_he_C of *R. ciliinervis* root, while the antioxidant activity diminishes concurrently with the decrease in TP_he_C.

#### 2.6.3. In Vitro Gastrointestinal Stability

To assess the stability of the methanol extract under gastrointestinal conditions, in vitro stability experiments that mimic the human digestive system were conducted. As depicted in Figure 5, over time, the TP_he_C values gradually augmented in the simulated oral and gastric environments, whereas in the simulated duodenal environment, the TP_he_C values abruptly declined and subsequently stabilized. This phenomenon might stem from the hydrolysis of bound phenolics triggered by the enzymatic system and the strong acidic environment of the gastrointestinal tract, leading to an elevation in TP_he_C values. However, upon the introduction of pancreatic enzymes, trypsin, and bile, the gastric acid is neutralized, creating a mildly alkaline pH. In this alkaline environment, a select few polyphenolic compounds undergo degradation, initially causing a transient dip in TP_he_C values before stabilizing. This observation is consistent with previously reported findings regarding pH stability. Additionally, excessive acidity had a pronounced effect on the ABTS radical scavenging capacity, which was partially restored under duodenal conditions, consistent with existing literature reports [92].

This study comprehensively analyzed the stability and antioxidant properties of phenolics in methanol extract of *R. ciliinervis* root under varying conditions. The results indicate that pH, heating time, and gastrointestinal conditions significantly impact the stability and antioxidant activity of these compounds. Optimal storage and handling conditions should consider maintaining a neutral or slightly acidic environment to preserve phenolic content and antioxidant activity.

### 2.7. Oxidative Stability of Oils

Fried foods are favored for their enticing taste and flavor. Nevertheless, during the frying process, exposure to high temperatures can encourage the oxidation and polymerization of oils, adversely impacting the quality of these foods [93]. To stabilize oils, the food industry frequently incorporates synthetic antioxidants into the oil. However, the utilization of synthetic antioxidants may pose safety concerns [94]. As a result, there is a mounting demand for natural antioxidants within the food industry. This experiment serves as a pivotal tool for assessing the antioxidant capability of methanol extract, emphasizing its medicinal value and potential to safeguard oils from oxidative degradation. The primary oxidation level of oils is typically gauged by measuring peroxide and acid values. Furthermore, K_232_ and K_270_ values are extensively employed to evaluate the primary and secondary stages of oil oxidation, respectively [95]. These indicators facilitate monitoring the oxidation status of oils, ensuring the quality and safety of fried foods.

**Figure 6:**
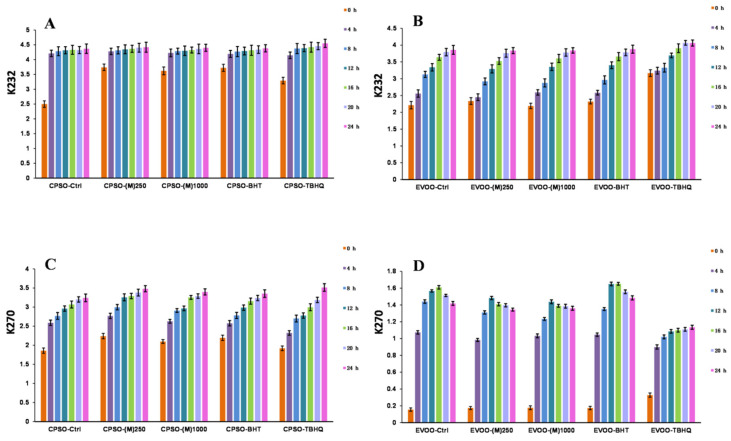
Variations in the levels of conjugated dienes (K_232_) and trienes (K_270_) in CPSO (**A**,**C**) and EVOO (**B**,**D**) supplemented with the synthetic antioxidants, BHT and TBHQ, and different concentrations of the methanol extract of *R. ciliinervis* root, at 160 °C. CPSO: cold-pressed sunflower oil; EVOO: extra virgin olive oil; BHT: butylated hydroxytoluene; TBHQ: tertiary butylhydroquinone.

**Figure 7:**
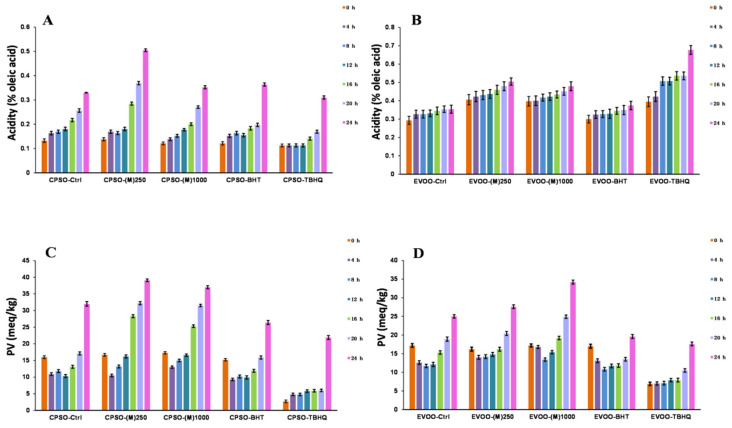
Variations in the levels of acidity values and PV in CPSO (**A**,**C**) and EVOO (**B**,**D**) supplemented with the BHT and TBHQ, and different concentrations of the methanol extract of *R. ciliinervis* root, at 160 °C. CPSO: cold-pressed sunflower oil; EVOO: extra virgin olive oil; PV: peroxide values; BHT: butylated hydroxytoluene; TBHQ: tertiary butylhydroquinone.

The study reveals that the inclusion of two test doses of methanol extract in extra virgin olive oil (EVOO) significantly diminished K_270_ values and outperformed BHT (Figure 6). As for acidity values, the addition of methanol extracts at these doses yielded superior results with acidity levels notably lower than those achieved with TBHQ (Figure 7). In cold-pressed sunflower oil (CPSO), the addition of two experimental doses of methanol extract produced a better K_232_ value than TBHQ, which was accompanied by a marked reduction in acidity values comparable to BHT’s effect (Figure 6). In the comprehensive study aimed at stabilizing peanut oil, as documented in reference [96], resveratrol has emerged as a standout performer, showcasing its prowess by decreasing peroxide and acidity values by an impressive 30%. This remarkable feat not only significantly extends the shelf life of peanut oil by more than twofold but also fortifies its thermal stability, ensuring a higher level of preservation. The exceptional attributes of resveratrol within this context elucidate its pivotal role as a primary component in the methanol extract, which has yielded outstanding outcomes for the two edible oils under investigation in the experiment. This research underscores the profound antioxidant potential of methanol extract when applied to edible oils, highlighting its effectiveness in mitigating acidity levels, which is a pivotal quality indicator that directly correlates with the extent of oil oxidation [97]. The reduction in acidity signifies a slower rate of oxidation, preserving the freshness, flavor, and nutritional value of the oil. Consequently, the findings of this study underscore the need for further exploration into the utilization of methanol extract as a potent antioxidant in edible oils.

### 2.8. Oral Acute Toxicity

An oral acute toxicity study was performed on mice to assess the acute toxicity of the methanol extract. Twenty mice were administered a single oral dose of 2000 mg/kg of the methanol extract. Encouragingly, none of the mice succumbed to any adverse effects within 24 h, suggesting that the methanol extract of *R. ciliinervis* root does not harbor highly toxic components and lacks significant acute toxicity. A previous report indicated that the intraperitoneal injection of *R. ciliinervis* root into mice yielded an LD_50_ of 2678 mg/kg over a 7 d observation period [28]. This LD_50_ value highlights the relatively low toxicity profile of *R. ciliinervis* root. “Chinese Materia Medica” documents that *R. ciliinervis* root possesses mild toxicity with a minority of patients reporting symptoms such as abdominal distension, nausea, vomiting, and hand numbness. Excessive consumption may also induce dizziness, which typically resolves upon cessation of use [98]. These effects may be attributed to the high anthraquinone content in *R. ciliinervis* root, notably emodin methyl ether, which in substantial doses can elicit diarrhea, headache, nausea, vomiting, muscle paralysis, and other symptoms [99]. Despite the widespread traditional usage of *R. ciliinervis* root, there exist a limited number of clinical research reports on its safety and efficacy. Consequently, there remains a dearth of extensive clinical research to fully support its safety and effectiveness. To comprehensively evaluate *R. ciliinervis* root’s suitability and potential health impacts on humans, further in-depth research, encompassing preclinical studies and comprehensive clinical trials, is imperative. However, it is crucial to note that the presence of Aloe emodin in methanol extract has been identified as a potential source of phototoxicity, hepatotoxicity, and nephrotoxicity, particularly when administered in high doses or over extended periods of time, as reported in [100]. Consequently, these aspects necessitate special attention and consideration in future toxicity research pertaining to the use of methanol extract in *R. ciliinervis* root.

### 2.9. Hepatoprotective Activity

The liver is a versatile organ with pivotal roles in numerous bodily functions. Chief among them are metabolizing carbohydrates, fats, and proteins, maintaining stable blood sugar levels, and synthesizing essential plasma proteins. Additionally, it serves as a crucial detoxification hub, breaking down harmful substances like alcohol and drugs. The liver secretes bile, which is vital for fat digestion and absorption, and it acts as a reservoir for iron, vitamins, and glycogen. It also synthesizes clotting factors, ensuring proper blood coagulation. Moreover, the liver possesses remarkable regenerative and immune defense capabilities, contributing to overall well-being. However, it is vulnerable to the adverse effects of conditions such as hepatitis, alcoholism, and fatty liver disease, leading to the release of enzymes when liver cells are compromised [101]. Thus, safeguarding liver health is paramount for preserving overall health and well-being, as it underpins numerous vital bodily processes.

*R. ciliinervis* root is primarily utilized as the primary medication in prescriptions and has been proven to exhibit therapeutic benefits in treating hepatitis, particularly with notable therapeutic effects. The underlying mechanism has been analyzed and attributed to the anthraquinone structure’s potent inhibitory action on the biosynthesis of viral cell DNA, RNA, and proteins [102]. Nevertheless, there remains a scarcity of extensive research reports specifically focusing on *R. ciliinervis* root therapy for liver diseases. Consequently, an experimental study was conducted using rats, wherein *D*-galactosamine was administered to induce liver injury with the aim of assessing the pre-protective effects of methanol extract on the liver. To quantify the severity of acute liver injury in the rats, various biochemical parameters were evaluated, including viscera index, liver enzyme activities, catalase (CAT) activities, malondialdehyde (MDA) and albumin levels. Notably, *D*-galactosamine is a well-established hepatotoxic agent that is capable of inducing liver damage (necrosis and inflammation) that closely mimics the pathology observed in human viral hepatitis [103].

**Figure 8:**
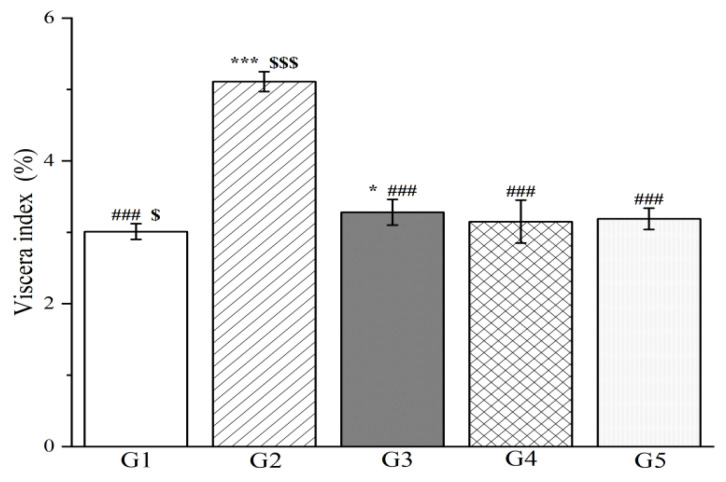
The outcomes of treatment with the methanol extract of *R. ciliinervis* root on the hepatic viscera index in rats with liver injury. Values are expressed as the mean ± standard error of the mean (*n* = 8). G1: control group, G2: *D*-GalN group, G3: *D*-GalN + SMN group, G4: *D*-GalN + *R. ciliinervis* root_300_ group, G5: *D*-GalN + *R. ciliinervis* root_150_ group. *D*-GalN: *D*-galactosamine; SMN: silymarin. Significantly different from the control group at * *p* < 0.05 and *** *p* < 0.001. Significantly different from the *D*-GalN group at ### *p* < 0.001. Significantly different from the *D*-GalN + SMN group at $ *p* < 0.05 and $$$ *p* < 0.001.

To evaluate the impact of methanol extract on liver injury, 40 rats were randomly assigned to five groups (*n* = 8) with each group receiving a distinct oral treatment regimen. The control group (G1) received 0.5% sodium carboxymethyl cellulose, while the model group (G2) was also administered 0.5% sodium carboxymethyl cellulose but served as the baseline for injury comparison. The positive control group (G3) was treated with silymarin at 100 mg/kg body weight, which is a known liver protectant. The high-dose group (G4) and low-dose group (G5) were administered methanol extract at 300 mg/kg and 150 mg/kg body weight, respectively, for seven consecutive days. Following this treatment period, all groups were administered orally for 7 d with the subsequent injection of *D*-galactosamine in G2 through G5. The results indicate that pretreatment with both high-dose and low-dose methanol extract significantly reduced the viscera index in rats compared to G2 (*p* < 0.001, Figure 8). Remarkably, there was no statistically significant difference in the pre-protective effects on liver function between the methanol extract (both high and low doses) and silymarin, highlighting the potential therapeutic value of methanol extract in mitigating liver injury.

**Figure 9:**
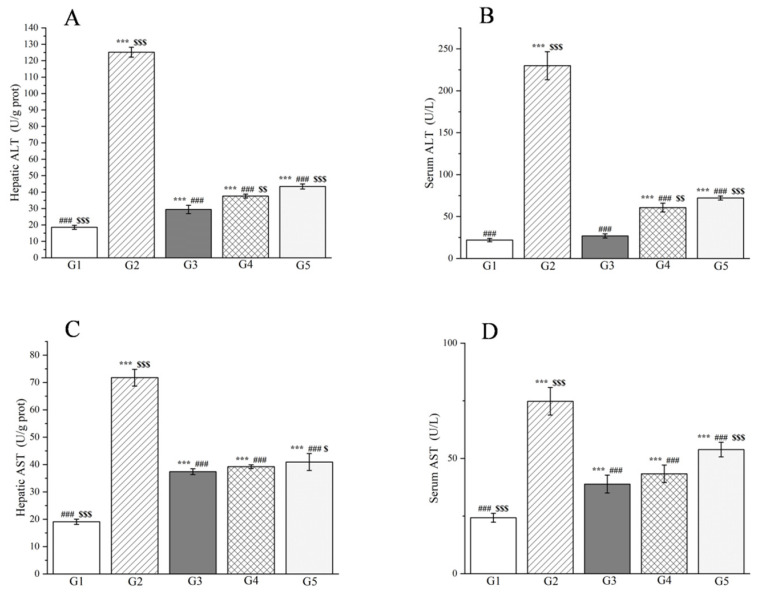
Effects of the methanol extract of *R. ciliinervis* root on hepatic ALT (**A**), AST (**C**) and serum ALT (**B**), AST (**D**) in rats with liver injury. Values are expressed as the mean ± standard error of the mean (*n* = 8). G1: control group, G2: *D*-GalN group, G3: *D*-GalN + SMN group, G4: *D*-GalN + *R. ciliinervis* root_300_ group, G5: *D*-GalN + *R. ciliinervis* root_150_ group. *D*-GalN: *D*-galactosamine; SMN: silymarin; ALT: alanine aminotransferase; AST: aspartate aminotransferase. Significantly different from the control group at *** *p* < 0.001. Significantly different from the *D*-GalN group at ### *p* < 0.001. Significantly different from the *D*-GalN + SMN group at $ *p* < 0.05, $$ *p* < 0.01 and $$$ *p* < 0.001.

**Figure 10:**
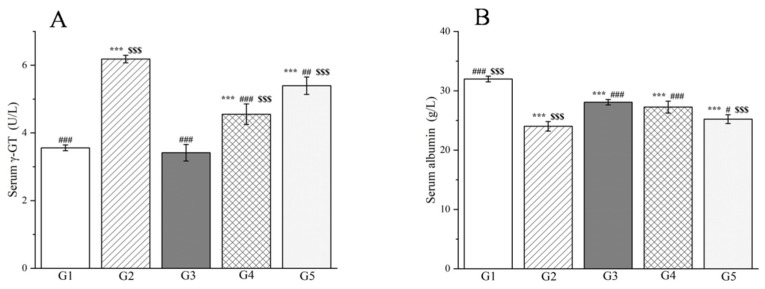
Effects of the methanol extract of *R. ciliinervis* root on serum *γ*-GT (**A**), ALB (**B**) in rats with liver injury. Values are expressed as the mean ± standard error of the mean (*n* = 8). G1: control group, G2: *D*-GalN group, G3: *D*-GalN + SMN group, G4: *D*-GalN + *R. ciliinervis* root_300_ group, G5: *D*-GalN + *R. ciliinervis* root_150_ group. *D*-GalN: *D*-galactosamine; SMN: silymarin; *γ*-GT: *γ*-glutamyl transpeptidase. Significantly different from the control group at *** *p* < 0.001. Significantly different from the *D*-GalN group at # *p* < 0.05, ## *p* < 0.01 and ### *p* < 0.001. Significantly different from the *D*-GalN + SMN group at $$$ *p* < 0.001.

Moreover, the research findings clearly demonstrate that the levels of alanine aminotransferase (ALT), aspartate aminotransferase (AST), and *γ*-glutamyl transpeptidase (*γ*-GT) in both the liver tissue and serum of the model group were markedly elevated compared to the control group (*p* < 0.001, Figure 9 and Figure 10), confirming the successful induction of an acute liver injury rat model. In terms of liver tissue, when compared with G2, the administration of methanol extract in the treatment groups led to a significant reduction in the activities of key liver enzymes, including ALT and AST (*p* < 0.001, Figure 9). Specifically, the ALT levels in G4 and G5 exhibited reductions of 69.9% and 65.3%, respectively. Similarly, AST levels decreased by 45.3% and 43.0% in these groups. These outcomes are comparable to those achieved with silymarin treatment, emphasizing the potential efficacy of the methanol extract in mitigating acute liver injury.

Simultaneously, the levels of ALT, AST, and *γ*-GT were measured in rat serum, which are enzymes that are predominantly localized within liver cells. However, upon liver cell damage, they are liberated into the bloodstream, resulting in an elevation of their serum levels. Experimental findings reveal that in comparison to G2, the methanol extract treatment groups significantly lowered the serum levels of both ALT and AST (*p* < 0.001, Figure 9) as well as the serum levels of *γ*-GT (*p* < 0.05, Figure 10). Specifically, the ALT levels in G4 and G5 decreased by 73.6% and 68.6%, respectively, when compared to G2. Analogously, the AST levels declined by 42.1% and 28.0%, respectively. Additionally, the *γ*-GT levels decreased by 26.4% and 12.7%, respectively. This reduction in liver enzyme activity serves as an indication of a decrease in liver injury and inflammation [104]. Our results affirm that the methanol extract from *R. ciliinervis* root exerts a pre-protective effect against *D*-galactosamine-induced liver injury, and this effect is dose-dependent.

In addition to liver enzymes, the albumin level in serum serves as a crucial indicator of liver function. The albumin level is directly correlated with the extent of liver function impairment; as the liver becomes damaged, the synthesis of albumin diminishes, leading to a decline in serum albumin levels. The study revealed that in comparison to G1, the albumin levels in rats from G2 were significantly reduced (*p* < 0.001, Figure 10). Nonetheless, when compared to G2, the administration of silymarin and a high-dose methanol extract resulted in a notable increase in albumin levels after 24 h (*p* < 0.001, Figure 10) with no discernible difference between the two treatments. Notably, the high-dose methanol extract demonstrated superior efficacy in enhancing albumin levels compared to the low-dose methanol extract.

**Figure 11:**
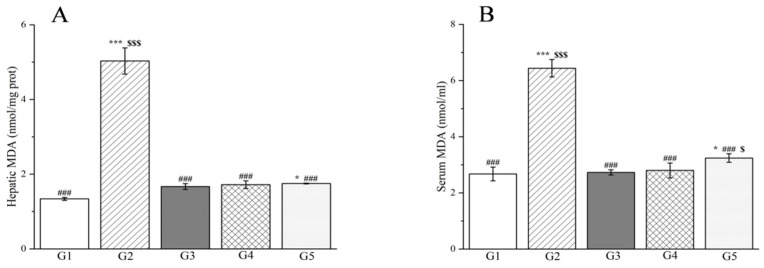
Effects of the methanol extract of *R. ciliinervis* root on hepatic MDA (**A**) and serum MDA (**B**) in rats with liver injury. Values are expressed as the mean ± standard error of the mean (*n* = 8). G1: control group, G2: *D*-GalN group, G3: *D*-GalN + SMN group, G4: *D*-GalN + *R. ciliinervis* root_300_ group, G5: *D*-GalN + *R. ciliinervis* root_150_ group. *D*-GalN: *D*-galactosamine; SMN: silymarin; MDA: malondialdehyde. Significantly different from the control group at * *p* < 0.05 and *** *p* < 0.001. Significantly different from the *D*-GalN group at ### *p* < 0.001. Significantly different from the *D*-GalN + SMN group at $ *p* < 0.05 and $$$ *p* < 0.001.

*D*-galactosamine not only triggers liver damage but also enhances the generation of ROS in the body while diminishing the efficacy of antioxidants, resulting in elevated MDA levels and CAT activity [105]. When compared to G1, the MDA levels in the liver and serum of G2 rats were markedly elevated subsequent to *D*-galactosamine treatment (*p* < 0.001, Figure 11). Notably, treatment with two doses of methanol extract in G2 rats significantly inhibited the increase in MDA levels in both liver tissue and serum, which were induced by liver injury (*p* < 0.001, Figure 11). Furthermore, this inhibitory effect was observed to intensify with an increase in the administered dose.

**Figure 12:**
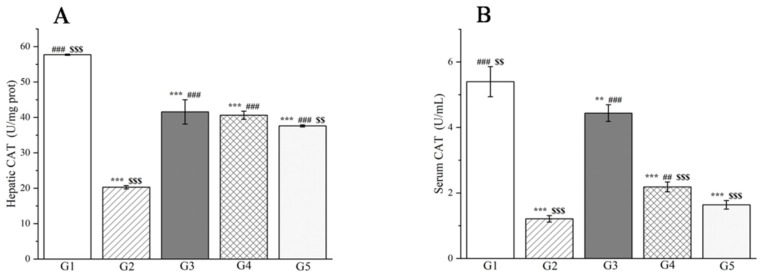
Effects of the methanol extract of *R. ciliinervis* root on hepatic CAT (**A**) and serum CAT (**B**) in rats with liver injury. Values are expressed as the mean ± standard error of the mean (*n* = 8). G1: control group, G2: *D*-GalN group, G3: *D*-GalN + SMN group, G4: *D*-GalN + *R. ciliinervis* root_300_ group, G5: *D*-GalN + *R. ciliinervis* root_150_ group. *D*-galactosamine; SMN: silymarin; CAT: catalase. Significantly different from the control group at ** *p* < 0.01 and *** *p* < 0.001. Significantly different from the *D*-GalN group at ## *p* < 0.01 and ### *p* < 0.001. Significantly different from the *D*-GalN + SMN group at $$ *p* < 0.01 and $$$ *p* < 0.001.

After the injection of *D*-galactosamine, the CAT levels in the liver and serum of G2 rats were significantly reduced (*p* < 0.001, Figure 12). In contrast, the CAT levels in the liver of rats treated with silymarin and the two doses of methanol extract were significantly increased compared to the G2 (*p* < 0.001, Figure 12). Notably, the therapeutic effect observed in the high-dose methanol extract group was comparable to that of silymarin. However, while both doses of methanol extract led to an increase in CAT levels in rat serum, the changes in the low-dose group were not statistically significant (*p* > 0.05, Figure 12). These findings suggest that the methanol extract of *R. ciliinervis* root can mitigate oxidative stress induced by *D*-galactosamine injection in rats to a certain degree, and the efficacy appears to correlate positively with the dose administered. Based on the aforementioned experimental results, it has been conclusively demonstrated that the methanol extract of *R. ciliinervis* root exerts a pre-protective effect against rat liver injury. This is achieved by reducing the levels of ALT, AST, *γ*-GT, and MDA while simultaneously increasing the levels of CAT and albumin. Furthermore, the high-dose methanol extract was found to be more effective than the low-dose extract, and its efficacy was comparable to that of silymarin.

There are reports indicating that the total anthraquinone content of *R. ciliinervis* root has the ability to reduce lactate dehydrogenase activity and MDA levels in tumor-bearing mice, inhibiting tumor growth [12,106]. Additionally, another study has reported that *R. ciliinervis* root polysaccharides can enhance the levels of CAT, SOD, and GSH-Px in mice with immune dysfunction induced by cyclophosphamide while concurrently inhibiting MDA production, demonstrating noteworthy antioxidant effects [6,107]. Notably, the primary constituent of *R. ciliinervis* root, polydatin, has been found to alleviate liver steatosis and damage caused by a methionine-choline deficient diet in mice. It significantly reduces serum ALT and AST levels, inhibits liver oxidative stress, decreases inflammation, and achieves hepatoprotective effects [108]. Resveratrol, a natural plant-derived antitoxin, possesses potent anti-inflammatory properties that have been demonstrated to exert beneficial effects on the liver. In the context of carbon tetrachloride-induced liver injury in mice, resveratrol has been shown to effectively reduce ALT, AST levels, necrosis, and 4-hydroxynonenal, mitigating liver damage. Furthermore, it reduces liver cell apoptosis while carefully modulating liver cell regeneration, contributing to the overall protection of liver function [109]. In summary, the aforementioned observations suggest that *R. ciliinervis* holds potential as a natural source of liver protectants and antioxidants.

**Figure 13:**
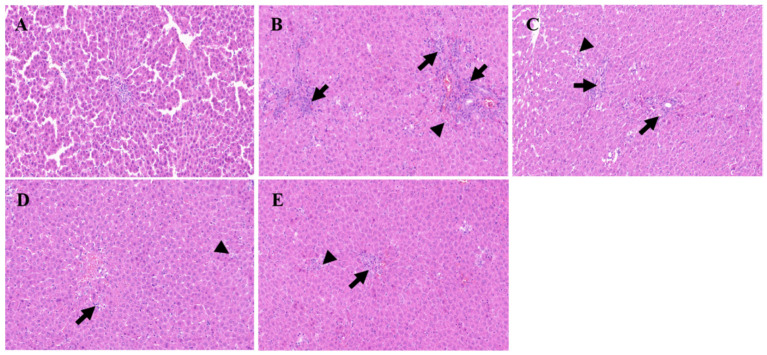
Histological examination of liver sections in different groups (200× magnification). (**A**): Control group (200× magnification); (**B**): *D*-GalN group (200× magnification); (**C**): *D*-GalN + SMN group (200× magnification); (**D**): *D*-GalN + *R. ciliinervis* root_300_ group (200× magnification); (**E**): *D*-GalN + *R. ciliinervis* root_150_ group (200× magnification). *D*-GalN: *D*-galactosamine; SMN: silymarin; no-tailed arrow: single-cell necrosis; long-tailed arrow: inflammatory cells.

The results of the histopathological examination are presented in Figure 13. In the control group (Figure 13A), the liver tissue structure of the rats remains intact, showcasing morphologically normal hepatocytes that are neatly arranged. The portal area is clearly visible, and there is no evidence of inflammatory cell infiltration. Conversely, the model group (Figure 13B) exhibits a disrupted liver tissue structure with the disappearance of hepatic cords. There is an increased cell density, and various-sized lipid droplets are observed within the cells, which are accompanied by cytoplasmic vacuoles. Notably, in regions adjacent to blood vessels, there is a pronounced infiltration of inflammatory cells (Figure 13B, long-tailed arrow) along with scattered single-cell necrosis (Figure 13B, no-tailed arrow). These necrotic cells exhibit reduced volume, enhanced cytoplasmic eosinophilia, and nuclear pyknosis with fragmentation. When compared to the model group, the positive group (Figure 13C) demonstrates significant improvement in hepatocyte damage, which manifested as scattered mild swelling and minimal fatty degeneration in a few hepatocytes. There is also a limited amount of inflammatory cell infiltration around the portal areas (Figure 13C, long-tailed arrow), although individual hepatocyte necrosis can still be observed (Figure 13C, no-tailed arrow). The high-dose group displays a similar pattern of inflammatory cell infiltration around the portal area (Figure 13D, long-tailed arrow), which is accompanied by mild hepatocyte swelling, reduced lipid droplet vacuoles in the cytoplasm, scattered hepatocyte degeneration, and single hepatocyte necrosis (Figure 13D, no-tailed arrow). Likewise, the low-dose group exhibits a small amount of inflammatory cell infiltration near the portal area (Figure 13E, long-tailed arrow), along with mild hepatocyte swelling, scattered hepatocyte degeneration, and individual hepatocyte necrosis (Figure 13E, no-tailed arrow). Notably, both dose groups show a reduction in the degree and extent of lesions compared to the model group with the high-dose group exhibiting a lesser lesion range akin to the positive group. In conclusion, the histopathological examination of the tissue provides a clear illustration of the damage inflicted by *D*-galactosamine on rat livers. It also offers an intuitive view of the protective effects of the positive group and the two dose groups on rat liver health, further emphasizing the potential therapeutic application of methanol extract of *R. ciliinervis* root in liver diseases.

## 3. Material and Methods

### 3.1. Materials

The *R. ciliinervis* root (voucher specimen number: 2023-08-23-002) was collected in Tonghua, which is located at a latitude of 41°13′39.8″ N, longitude of 126°18′54.5″ E, and an altitude of 206.1 m, in Jilin Province, China, in August 2023. The identity of the specimen was verified by a plant taxonomist, Professor Junlin Yu. The voucher specimen is currently being stored in the Herbarium of Tonghua Normal University.

### 3.2. Methods

#### 3.2.1. Qualitative Phytochemical Analysis

A qualitative phytochemical analysis was conducted on 15 distinct chemical components, adhering to a previously established methodology [110]. This analysis aimed to identify the various chemical constituents present within the sample. For detailed experimental procedures, please refer to the Supplementary Materials.

#### 3.2.2. Preparation of Different Extracts of *R. ciliinervis* Root

To investigate the effect of various solvents on the extraction efficiency of active ingredients from *R. ciliinervis* root, this experiment utilized four different polar solvents: methanol, water, ethanol, and 80% ethanol. The extraction process was carried out according to the previously established methodology [110]. For detailed experimental procedures, please refer to the Supplementary Materials.

#### 3.2.3. Quantitative Phytochemical Analysis

A quantitative phytochemical analysis was performed to ascertain the concentrations of various compounds, including TCC, TP_ro_C, TP_he_C, TSC, TAC, TFC, TPAC, TT_an_C, GC, and CTC. These measurements were conducted using methodologies previously outlined in references [110,111]. For detailed experimental procedures, please refer to the Supplementary Materials.

#### 3.2.4. Antioxidant Activity Assays

Antioxidant activity assays were conducted utilizing a diverse array of methods, including DPPH, ABTS, hydroxyl radical, superoxide radical, FRAP, CUPRAC, metal chelation, H_2_O_2_, singlet oxygen, and *β*-carotene bleaching assays. These assays were performed in accordance with previously established protocols [111]. For detailed experimental procedures, please refer to the Supplementary Materials.

#### 3.2.5. UHPLC-MS Analysis

The experimental conditions for UHPLC-MS were implemented according to the previously described methodology [95], ensuring the precision and reproducibility of the analytical process. For detailed experimental procedures, please refer to the Supplementary Materials.

#### 3.2.6. Stability Studies of Methanol Extract

The pH stability, thermal stability, and stability in a gastrointestinal tract model system of the methanol extract of *R. ciliinervis* root were evaluated using methods previously outlined in the research [95]. For detailed experimental procedures, please refer to the Supplementary Materials.

#### 3.2.7. Oxidative Stability Studies of Oils

The oxidative stability of EVOO and CPSO was assessed using methods previously described in the research [95]. For detailed experimental procedures, please refer to the Supplementary Materials.

#### 3.2.8. Oral Acute Toxicity Study

An acute oral toxicity study was conducted in accordance with the established protocols outlined in the previous methods [95], aiming to evaluate the potential adverse effects of the methanol extract under investigation when administered orally in a single dose. For detailed experimental procedures, please refer to the Supplementary Materials.

#### 3.2.9. Hepatoprotective Experiments

The hepatoprotective experiments were conducted in adherence to the procedures outlined in reference [95], encompassing animal selection, experimental protocol design, histopathological examination, and biochemical analyses. For detailed experimental procedures, please refer to the Supplementary Materials.

#### 3.2.10. Statistical Analysis

Statistical significance between groups was determined using a one-way analysis of variance, which was followed by post hoc least significant difference tests and DUNCAN tests. *p*-values of 0.05, 0.01, and 0.001 were considered as indicative of significance, high significance, and very high significance, respectively.

## 4. Conclusions

This study systematically investigated *R. ciliinervis* root through a combination of qualitative and quantitative phytochemical analysis methods along with in vitro antioxidant activity assays. The findings revealed that the methanol extract of *R. ciliinervis* root was abundant in diverse active constituents and demonstrated remarkable performance in antioxidant tests, prompting its selection for further examination. Utilizing UHPLC-MS technology, a total of 45 compounds were identified within the methanol extract. Additionally, stability assessments of the methanol extract under various conditions, including heating, varying pH levels, and simulated human digestive system conditions, demonstrated significant stability of its antioxidant active ingredients and robust antioxidant capacity. Furthermore, oil stability experiments highlighted the potential of this extract as an effective antioxidant for stabilizing edible oils, positioning it as a promising candidate for applications in food preservation, particularly in the stabilization of edible oils.

In the experiment involving *D*-galactosamine-induced liver injury in rats, histopathological evaluations and biochemical markers confirmed a significant pre-protective effect of the methanol extract on the rat liver. In conclusion, our research emphasizes the potent antioxidant properties and protective effects of methanol extract of *R. ciliinervis* root against liver damage stemming from oxidative stress. These findings serve as a foundation for future investigations into the therapeutic potential of this plant in addressing oxidative stress-related disorders, such as neurodegenerative conditions, and cardiovascular complications. Looking ahead, the acute toxicity study of serves as an initial safety assessment, emphasizing the need for more extensive toxicological evaluations to ensure the safe and efficacious integration of this extract into clinical practice or consumer products. Future research should also delve into the mechanisms underlying its antioxidant and protective effects, exploring potential synergistic interactions among the identified compounds.

## Figures and Tables

**Table 1:** Extraction yields of *R. ciliinervis* root extracted with different solvents.

Extracting Solvents	Yields (%, *w*/*w*)
Water	25.50 ± 0.12 ^a^
Methanol	21.97 ± 0.09 ^a,b^
Ethanol	22.11 ± 0.14 ^a^
80% Ethanol	25.07 ± 0.47 ^b^

^a–b^ Columns with different superscripts indicate a significant difference (*p* < 0.05).

**Table 2:** Total carbohydrate content (TCC), total protein content (TP_ro_C), total phenolic content (TP_he_C), total steroid content (TSC), total alkaloid content (TAC), total flavonoid content (TFC), total phenolic acid content (TPAC), total tannin content (TT_an_C), gallotannin content (GC), and condensed tannin content (CTC) of *R. ciliinervis* root extracted with different solvents.

Extracting Solvents	TCC (mg GE/g Extract)	TP_ro_C (mg BSAE/g Extract)	TP_he_C (mg GAE/g Extract)	TSC (mg OAE/g Extract)	TAC (mg BHE/g Extract)	TFC (mg QE/g Extract)	TPAC (mg CAE/g Extract)	TT_an_C (mg TAE/g Extract)	GC (mg GAE/g Extract)	CTC (mg GAE/g Extract)
Water	676.51 ± 3.63 ^b,c^	1233.90 ± 14.71 ^a^	89.18 ± 1.98 ^d^	NONE	64.75 ± 5.59 ^a^	2.73 ± 0.16 ^d^	3.71 ± 0.23 ^d^	75.17 ± 0.79 ^c^	69.03 ± 1.29 ^c^	43.03 ± 2.32 ^c^
Methanol	686.61 ± 12.58 ^b^	N.T.	157.33 ± 1.31 ^c^	3.44 ± 0.08 ^a^	52.66 ± 4.08 ^b^	10.84 ± 0.54 ^a^	11.05 ± 0.64 ^a^	154.82 ± 1.04 ^a^	123.42 ± 1.00 ^a^	85.70 ± 1.83 ^b^
Ethanol	663.23 ± 2.94 ^c^	N.T.	180.61 ±1.59 ^a^	2.16 ± 0.19 ^b^	45.65 ± 2.31 ^b^	7.09 ± 0.63 ^b^	7.12 ± 0.19 ^b^	141.77 ± 2.30 ^b^	125.64 ± 1.92 ^a^	87.10 ± 0.66 ^b^
80% Ethanol	749.49 ± 7.06 ^a^	N.T.	176.63 ± 1.91 ^b^	0.75 ± 0.04 ^c^	35.66 ± 2.69 ^c^	5.51 ± 0.36 ^c^	6.01 ± 0.36 ^c^	153.37 ± 0.96 ^a^	121.89 ± 0.80 ^b^	92.11 ± 2.11 ^a^

^a–d^ Columns with different superscripts indicate a significant difference (*p* < 0.05). N.T. indicates no test. GE: glucose equivalents; BSAE: bovine serum albumin equivalents; GAE: gallic acid equivalents; OAE: oleanolic acid equivalents; BHE: berberine hydrochloride equivalents; QE: quercetin equivalents; CAE: caffeic acid equivalents; TAE: tannic acid equivalents.

**Table 3:** Determination of antioxidant activity of various solvent extracts of *R. ciliinervis* root using DPPH, ABTS, hydroxyl, and superoxide radicals.

Extracting Solvents	DPPH (IC_50_, μg/mL)	ABTS (IC_50_, μg/mL)	Hydroxyl Radicals (IC_50_, μg/mL)	Superoxide Radicals (IC_50_, μg/mL)
Water	29.46 ± 0.48 ^e^	11.96 ± 0.14 ^e^	1666.59 ± 15.58 ^g^	89.95 ± 0.97 ^d^
Methanol	3.15 ± 0.02 ^b^	5.95 ± 0.11 ^d^	489.71 ± 13.65 ^f^	38.02 ± 1.64 ^c^
Ethanol	4.08 ± 0.15 ^c^	5.17 ± 0.15 ^c^	326.93 ± 11.64 ^d^	24.36 ± 0.80 ^b^
80% Ethanol	3.18 ± 0.09 ^b^	5.75 ± 0.26 ^d^	403.83 ± 6.28 ^e^	21.37 ± 1.04 ^a^
*L*-ascorbic acid *	1.80 ± 0.06 ^a^	3.25 ± 0.11 ^a^	92.63± 0.19 ^a^	N.T.
Trolox *	2.04 ± 0.10 ^a^	3.36 ± 0.06 ^a^	133.53 ± 1.88 ^b^	N.T.
BHT *	9.33 ± 0.08 ^d^	4.44 ± 0.05 ^b^	244.87 ± 2.31 ^c^	N.T.
Curcumin *	N.T.	N.T.	N.T.	212.56 ± 3.08 ^e^

^a–g^ Columns with different superscripts indicate a significant difference (*p* < 0.05). * Used as a standard antioxidant; BHT: butylated hydroxytoluene; N.T. indicates no test.

**Table 4:** Determination of antioxidant activity of various solvent extracts of *R. ciliinervis* root using FRAP, CUPRAC, and metal chelating.

Extracting Solvents	TEAC_FRAP_	TEAC_CUPRAC_	Iron Chelating (IC_50_, μg/mL)	Copper Chelating (IC_50_, μg/mL)
Water	0.16 ± 0.00 ^e^	0.23 ± 0.01 ^c^	>2500 ^e^	920.30 ± 22.71 ^d^
Methanol	0.29 ± 0.00 ^d^	0.80 ± 0.05 ^b^	662.81 ± 11.18 ^b^	308.96 ± 6.09 ^b^
Ethanol	0.31 ± 0.00 ^c^	0.79 ± 0.04 ^b^	1218.11 ± 26.50 ^d^	396.11 ± 2.95 ^c^
80% Ethanol	0.29 ± 0.01 ^d^	0.77 ± 0.01 ^b^	899.10 ± 13.94 ^c^	408.60 ± 6.95 ^c^
Trolox *	1.00 ± 0.01 ^a^	1.00 ± 0.01 ^a^	N.T.	N.T.
EDTANa_2_ *	N.T.	N.T.	2.33 ± 0.99 ^a^	41.60 ± 1.90 ^a^

^a–e^ Columns with different superscripts indicate a significant difference (*p* < 0.05). * Used as a standard antioxidant; EDTANa_2_: ethylenediaminetetraacetic acid disodium salt; N.T. indicates no test.

**Table 5:** Determination of antioxidant activity of various solvent extracts of *R. ciliinervis* root using H_2_O_2_, singlet oxygen, and *β*-carotene bleaching assays.

Extracting Solvents	H_2_O_2_ (IC_50_, μg/mL)	Singlet Oxygen (IC_50_, μg/mL)	*β*-Carotene Bleaching AAC
Water	755.15 ± 45.27 ^e^	>2500 ^e^	614.06 ± 38.90 ^c^
Methanol	428.93 ± 8.52 ^b^	800.36 ± 24.95 ^b^	1025.56 ± 55.57 ^a,b^
Ethanol	489.54 ± 10.31 ^c^	925.70 ± 29.19 ^c^	1027.79 ± 50.01 ^a,b^
80% Ethanol	573.37 ± 15.86 ^d^	810.23 ± 34.36 ^b^	1103.08 ± 55.57 ^a^
Gallic acid *	20.13 ± 1.18 ^a^	N.T.	N.T.
Ferulic acid *	N.T.	443.0 ± 8.7 ^a^	N.T.
BHT *	N.T.	N.T.	949.99 ± 41.68 ^b^
BHA *	N.T.	N.T.	956.93 ± 50.01 ^b^
TBHQ *	N.T.	N.T.	957.21 ± 38.90 ^b^

^a–e^ Columns with different superscripts indicate a significant difference (*p* < 0.05). * Used as a standard antioxidant; BHT: butylated hydroxytoluene; BHA: butyl hydroxyanisole; TBHQ: tertiary butylhydroquinone; N.T. indicates no test.

**Table 6:** Compounds identified in methanol extract of *R. ciliinervis* root.

Peak No.	RT (min)	Identification	Molecular Formula	Selective Ion	Full Scan MS (*m*/*z*)		MS/MS Fragments (*m*/*z*)
					Theory	Measured	
1	0.88	*D*-Sucrose	C_12_H_22_O_11_	[M+NH_4_]^+^	360.1506	360.1417	343.1175, 180.0820, 163.0560
2	1.18	Glutamylglycine	C_7_H_12_N_2_O_5_	[M+NH_4_]^+^	222.1090	222.1066	163.0339, 91.0519
3	1.27	Unknown				292.1318	153.0145, 136.0722
4	1.36	Citramalic acid	C_5_H_8_O_5_	[M+Na]^+^	171.0270	171.0241	132.0983, 103.0515
5	3.23	Epigallocatechin/Gallocatechin	C_15_H_14_O_7_	[M+H]^+^	307.0818	307.0742	289.0641, 181.0462
6	4.54	Phenylalanine	C_9_H_11_NO_2_	[M+Na]^+^	188.0688	188.0657	165.0507, 100.0730
7	6.04	Procyanidin B1	C_30_H_26_O_12_	[M+H]^+^	579.1502	579.1385	453.1047, 289.0649
8	6.84	*D*-(+)-Catechin/(−)-Epicatechin	C_15_H_14_O_6_	[M+H]^+^	291.0868	291.0798	273.0710, 165.0512
9	7.29	Caffeic acid 3-glucoside	C_15_H_18_O_9_	[M+Na]^+^	365.0849	365.0759	297.0541, 181.0453
10	9.44	*β*-Syringin	C_17_H_24_O_9_	[M+Na]^+^	395.1318	395.1254	227.1697, 194.1131
11	10.31	2-Phenylethyl 6-*O*-*β*-*D*-xylopyranosyl-*β*-*D*-glucopyranoside	C_19_H_28_O_10_	[M+Na]^+^	439.1580	439.1509	340.2528, 322.2425
12	10.46	1,3,6-Trigalloyl glucose	C_27_H_24_O_18_	[M+NH_4_]^+^	654.1307	654.1180	467.0745, 449.0634
13	10.56	*N*-Acetylmethionine	C_14_H_18_O_9_	[M]^+^	191.0616	191.0653	177.0044, 133.0623
14	10.99	Uridine	C_9_H_12_N_2_O_6_	[M+H]^+^	245.0773	245.0748	227.1707, 133.0612
15	11.97	Chrysoeriol 7-*O*-glucoside	C_22_H_22_O_11_	[M+H]^+^	463.1240	463.1131	340.2531, 283.0540, 163.0340
16	14.01	(+)-Lyoniresinol-3*a*-*O*-*β*-glucoside	C_28_H_38_O_13_	[M+NH_4_]^+^	600.2656	600.2537	340.2528, 163.0143
17	14.25	Polydatin	C_20_H_22_O_8_	[M+H]^+^	391.1393	391.1310	211.0708, 107.0461
18	15.79	Polygonimitin B	C_21_H_22_O_9_	[M+H]^+^	419.1342	419.1234	257.0745, 163.0150
19	16.97	Liquiritin	C_21_H_22_O_9_	[M+H]^+^	419.1342	419.1248	257.0747, 222.1077
20	17.86	Unknown				183.0729	133.0826, 89.0570
21	18.73	Resveratrol	C_14_H_12_O_3_	[M+H]^+^	229.0864	229.0796	229.0810, 127.0123
22	19.53	Cassiachromone	C_13_H_12_O_4_	[M+H]^+^	233.0814	233.0745	215.0650, 205.0547
23	20.94	Emodin 8-*β*-*D*-glucopyranoside	C_21_H_20_O_10_	[M+Na]^+^	455.0954	455.0837	271.0545, 197.0547
24	22.63	Resveratrol-3-*O*-(2″-*O*-galloyl)-*β*-*D*-glucopyranoside	C_27_H_26_O_12_	[M+H]^+^	543.1502	543.1372	245.1807, 192.1337
25	25.74	Torachrysone 8-*O*-glucoside	C_20_H_24_O_9_	[M+H]^+^	409.1498	409.1396	431.1235, 391.1313, 229.0809
26	26.11	Aloe emodin	C_15_H_10_O_5_	[M+H]^+^	271.0606	271.0528	225.0495, 197.0546
27	26.91	Unknown				309.0892	291.0800, 147.0401
28	27.55	7-Methoxycoumarin	C_10_H_8_O_3_	[M+H]^+^	177.0551	177.0495	149.0557, 145.0244, 117.0301
29	28.41	Piscidic acid	C_11_H_12_O_7_	[M]^+^	256.0583	256.0662	222.1073, 194.1128
30	28.53	Emodin	C_15_H_10_O_5_	[M+H]^+^	271.0606	271.0531	163.0348, 107.0464
31	29.41	1-Methyl emodin	C_16_H_12_O_5_	[M+H]^+^	285.0763	285.0684	201.0858, 143.0363
32	30.47	Emodin 3-methyl ether	C_16_H_12_O_5_	[M+H]^+^	285.0763	285.0686	242.0522, 222.1077
33	31.47	Emodin anthrone	C_15_H_12_O_4_	[M]^+^	256.0736	256.0666	224.1228, 194.1128
34	32.67	Physicon-8-*β*-*D*-(6′-*O*-acetyl)glucoside	C_24_H_24_O_11_	[M+Na]^+^	511.1217	511.1279	453.3350, 205.0554
35	33.37	Unknown				285.0686	270.0827, 222.1075
36	33.97	Piceid-2″-*O*-conmarate	C_29_H_28_O_9_	[M+H]^+^	521.1811	521.1683	293.0960, 275.0852
37	37.82	Palmitic acid	C_16_H_32_O_2_	[M+NH_4_]^+^	274.2746	274.2673	212.2320, 71.0831, 57.0679
38	38.23	Phytosphingosine	C_18_H_39_NO_3_	[M+H]^+^	318.3008	318.2925	183.0735, 155.0428, 127.0123
39	39.83	Linoleic acid	C_18_H_32_O_2_	[M+NH_4_]^+^	298.2746	298.2663	235.1866, 222.1074
40	41.25	Unknown				415.2021	340.2524, 183.0737
41	42.49	Sphinganine	C_18_H_39_NO_2_	[M+H]^+^	302.3059	302.2980	276.1902, 183.0741, 100.0730
42	43.85	Unknown				280.2560	263.2314, 222.1071
43	44.47	Stearidonic acid	C_18_H_28_O_2_	[M+NH_4_]^+^	294.2433	294.2350	277.2105, 249.2150
44	44.58	Eicosapentanoic acid	C_20_H_30_O_2_	[M+NH_4_]^+^	320.2590	320.2480	280.2576, 135.0404, 123.0885
45	45.93	*α*-Linolenic acid	C_18_H_30_O_2_	[M+NH_4_]^+^	296.2590	296.2528	279.2264, 149.1033, 135.0404
46	46.52	14-Methylpentadecanoic acid	C_16_H_32_O_2_	[M+Na]^+^	279.2300	279.2245	222.1077, 183.0734, 127.0123
47	47.19	1-Palmitoyl-2-hydroxy-sn-glycero-3-PE	C_21_H_44_NO_7_P	[M+NH_4_]^+^	471.3199	471.3196	155.0432, 140.1148
48	48.35	Erucamide	C_22_H_43_NO	[M+H]^+^	338.3423	338.3331	279.1536, 237.2582, 149.0196
49	48.92	*β*-Sitosterol	C_29_H_50_O	[M+Na]^+^	437.3760	437.3736	407.3290, 338.3350

RT: Retention time.

## Data Availability

The original contributions presented in the study are included in the article/Supplementary Materials, further inquiries can be directed to the corresponding authors.

## Supplementary Materials

The following supporting information can be downloaded at https://www.mdpi.com/article/10.3390/molecules29194701/s1. Table S1: Qualitative phytochemical analysis of *R. ciliinervis* root; Figure S1: Chemical structures of the compounds identified in methanol extract of *R. ciliinervis* root; Figure S2: Positive-ion mode UHPLC-MS findings of methanol extract of *R. ciliinervis* root; Figure S3–Figure S100: Presentation of MS and MS/MS spectra for peaks 1 through 49.
