# Supplementary material for: Evaluating the Root Extract of Reynoutria ciliinervis (Nakai) Moldenke: An Analysis of Active Constituents, Antioxidant Potential, and Investigation of Hepatoprotective Effects in Rats
Source: Molecules. 2024 Oct 4;29(19):4701. doi: 10.3390/molecules29194701 (PMC11478139; doi:10.3390/molecules29194701)
Supplement: Supplementary file 1 [file molecules-29-04701-s001.zip › molecules-3228238-supplementary.pdf]

SUPPLEMENTARY MATERIAL

Evaluating the Root Extract of Reynoutria ciliinervis (Nakai) Moldenke: An Analysis of Active Constituents, Antioxidant Potential, and Investigation of Hepatoprotective Effects in Rats

Zheng Xing [1,2,†], Yang Han [1,†], Hao Pang [1,2], Li Li [2,3], Guangqing Xia [2,3], Junyi Zhu [2,3], Jing Han [1,*] and Hao Zang [1,2,3,*]

1 School of Pharmaceutical Engineering, Shenyang Pharmaceutical University, Benxi 117004, China
2 School of Pharmacy and Medicine, Tonghua Normal University, Tonghua 134002, China
3 Key Laboratory of Evaluation and Application of Changbai Mountain Biological Gerplasm Resources of Jilin Province, Tonghua 134002, China
* Correspondence: hanjing@syphu.edu.cn (J.H.); zanghao@thnu.edu.cn (H.Z.);
Tel.: +86-024-4352-0212 (J.H.); +86-435-320-2678 (H.Z.)
† These authors contributed equally to this work.

Reagents and Chemicals

p-Nitroblue tetrazolium chloride (NBT) was purchased from Sigma-Aldrich. Curcumin, salicylic acid, *L*-ascorbic acid, 2,4,6-tri(2-pyridyl)-s-triazine (TPTZ), ammonium acetate (NH_4Ac), cupric sulphate, ferrous sulfate heptahydrate ($FeSO_4·7H_2O$), copper sulphate ($CuSO_4$), 4-aminoantipyrine, lipoic acid, ferulic acid, sulfanilamide, cupric chloride dihydrate ($CuCl_2·2H_2O$), phosphoric acid (H_3PO_4), ninhydrin hydrate, quercetin, D-(+)-glucose, butylated hydroxytoluene (BHT), 2,9-dimethyl-1,10-phenanthroline (Neocuproine, Nc), α-naphthol, iodine, tertiary butylhydroquinone (TBHQ), 3,5-dinitrosalicylic acid (DNS), gelatin, potassium iodide (KI), ferric chloride ($FeCl_3$), 4-nitroaniline, sodium nitrite, antimony trichloride, calcium hydroxide ($Ca(OH)_2$), ABTS, copper sulfate pentahydrate ($CuSO_4·5H_2O$), phosphomolybdic acid hydrate, hydroxylamine hydrochloride, potassium hydroxide, vanillin, 3,5-dinitrobenzoic acid, phenol, dipotassium hydrogen phosphate, potassium dihydrogen phosphate, sodium dihydrogen phosphate, dibasic sodium phosphate, sodium hypochlorite (NaClO) (10% active chloride), tannic acid, potassium persulfate, potassium chloride (KCl), sodium acetate, gallic acid, sodium molybdate, arbutin, *L*-tyrosine, urea, phloroglucinol, potassium iodate, oleanolic acid were purchased from Energy Chemical. Benedict's Reagent was purchased from Adamas. DPPH was purchased from Alfa Aesar. Bromocresol green, Trolox, pyrocatechol violet, Sudan III, and Sudan IV were purchased from TCI. Folin and Ciocalteu's phenol reagent (FC reagent), aluminum chloride hexahydrate ($AlCl_3·6H_2O$), linoleic acid, 3-(2-pyridyl)-5,6-diphenyl-1,2,4-triazine-4′,4″-disulfonic acid sodium salt (Ferrozine), ferrous chloride tetrahydrate ($FeCl_2·4H_2O$), sodium potassium tartrate tetrahydrate (Rochelle salt), ethylenediaminetetraacetic acid disodium salt dihydrate ($EDTANa_2·2H_2O$), potassium ferricyanide ($K_3[Fe(CN)_6]$), Lead(II) acetate trihydrate, tungstosilicic acid hydrate, bismuth subnitrate, mercury(II) chloride ($HgCl_2$), pepsin (32 U/mg), pancreatin, bovine bile extract, magnesium acetate, sodium thiosulfate standard solution (0.1 M), potassium hydroxide standard solution (0.1 M), phenolphthalein, tween 40, and 1,3-dinitrobenzene were purchased from Xiya Reagent. Concentrated sulfuric acid (H_2SO_4), phenol, sodium carbonate (Na_2CO_3), methanol, ethanol, acetone, ethyl acetate, dichloromethane, hexane, dimethyl sulfoxide (DMSO), petroleum ether (60–90°C), sodium hydroxide (NaOH), concentrated hydrochloric acid (HCl), sodium chloride (NaCl), magnesium powder, acetic acid, ammonium hydroxide ($NH_3·H_2O$), acetic anhydride, 30% hydrogen peroxide (H_2O_2), formaldehyde, and 3% bromine water were purchased from Sinopharm. All reagents and solvents used were analytical grade. Blue litmus paper was purchased from Tianjin Jinda Chemical Reagent Co., Ltd. BCA kit was purchased from Beyotime. Trypsin (2500 U/mg) was purchased from Aladdin (Bay City, MI, USA).

Preparation of Preliminary Experimental Solutions for Qualitative Phytochemical Analysis

Aqueous Extraction Solutions

Reynoutria ciliinervis (Nakai) Moldenke (*R. ciliinervis*) root powder (5 g) was weighed and passed through a sieve (20 mesh). Distilled water (50 mL) was added and the mixture was allowed to stand overnight at room temperature. Next, 5 mL of filtrate was obtained by filtration, and this filtrate was analysed to check for amino acids and proteins. The remaining residue and leaching solution were heated at 60°C for 10 min. After heating, the mixture was filtered immediately. This filtrate was used to check for carbohydrates, organic acids, saponins, glycosides, phenolics, tannins and cyanogenic glycosides.

Methanol Extraction Solutions

R. ciliinervis root powder (5 g) was weighed and passed through a sieve (20 mesh). Ethyl ether (50 mL) was added and the mixture was heated under reflux for 10 min. The filter residue was transferred back into the bottle after filtration. Next, 35 mL of methanol was added and the mixture was heated under reflux for 10 min. After heating, the mixture was filtered immediately. This filtrate was used to check for flavonoids, anthraquinones, cardiac glycosides, coumarins and lactones, volatile oils, terpenoids, steroids, lipids and alkaloids.

Petroleum Ether Extraction Solutions

R. ciliinervis root powder (3 g) was weighed and passed through a sieve (20 mesh). Petroleum ether (15 mL) was added and the mixture was allowed to stand at room temperature for 4 h. Next, 5 mL of filtrate was obtained by filtration, and this filtrate was analysed to check for volatile oils, lipids, steroids and triterpenoids.

Qualitative Phytochemical Analysis

Tests for Proteins/Amino Acids

Ninhydrin Tests

1 mL of aqueous extraction solution was mixed with 1 mL of 0.2% ninhydrin solution. The mixture was boiled for 5 min. Development of a purple colour indicated the presence of amino acids or proteins.

Biuret Tests

1 mL of aqueous extraction solution was mixed with 1 mL of solution A (0.1 g/mL NaOH), and then two drops of solution B (0.01 g/mL $CuSO_4$) were added. This mixture was shaken, and a purple, red, or purplish-red colour indicated the presence of amino acids or proteins.

Tests for Carbohydrates

Fehling's Tests

Equal volumes of solution A (34.66 g of $CuSO_4{\cdot}5H_2O$ dissolved in 500 mL of distilled water) and solution B (173 g of sodium potassium tartrate tetrahydrate and 50 g of NaOH dissolved in 500 mL of distilled water) were mixed together. A sample (1 mL) of this mixture was then mixed with 1 mL of aqueous extraction solution. The resulting mixture was boiled gently. Formation of a brick-red precipitate indicated the presence of reducing sugars.

Benedict's Tests

1 mL of aqueous extraction solution was mixed with 1 mL of Benedict's reagent and the resulting mixture was boiled gently. Formation of a reddish-brown precipitate indicated the presence of carbohydrates.

Molisch's Tests

1 mL of aqueous extraction solution was mixed with 1 mL of Molisch's solution (2 g of α-naphthol dissolved in 100 mL of 95% ethanol). The mixture was then poured carefully into another test tube containing 1 mL of H_2SO_4. A purple ring at the aqueous phase/organic phase interface indicated the presence of carbohydrates.

Iodine Tests

1 mL of aqueous extraction solution was mixed with 1 mL of iodine solution (127 mg of iodine and 200 mg of KI dissolved in 10 mL of distilled water). Development of a dark blue or purple colour indicated the presence of carbohydrates.

Tests for Phenolics

$FeCl_3$ Tests

1 mL of aqueous extraction solution was mixed with 1 mL of 2% $FeCl_3$ solution. Development of a blue-green or black colour indicated the presence of phenolics.

$FeCl_3$-$K_3[Fe(CN)_6]$ Tests

A few drops of aqueous extraction solution were added onto a thin-layer chromatography plate, and the chromogenic reagent (1% $K_3[Fe(CN)_6]$ solution was mixed with 2% $FeCl_3$ solution in equal volumes) was sprayed onto the plate, thus generating a blue colour. Then, 2 M HCl was sprayed onto the plate, and a darker colour indicated the presence of phenolics.

Diazotization Tests

1 mL of aqueous extraction solution was mixed with 1 mL of 3% Na_2CO_3 solution. The resulting mixture was boiled for 3 min and then cooled in ice water. Two drops of newly prepared diazotization reagent were added. Development of a red colour indicated the presence of phenolics.

Tests for Organic Acids

pH Tests

The pH of aqueous extraction solution was measured with pH meter. A pH value below 7.0 indicated the presence of organic acids.

Blue Litmus Paper Tests

A few drops of aqueous extraction solution were placed on a blue litmus paper. Development of a red colour indicated the presence of organic acids.

Bromocresol Green Tests

A few drops of aqueous extraction solution were added onto a thin-layer chromatography plate. Chromogenic reagent (0.1 g of bromocresol green dissolved in 500 mL of ethanol and mixed with 5 mL of 0.1 N NaOH) was sprayed onto the plate. Development of a yellow colour on a blue background indicated the presence of organic acids.

Tests for Tannins

$FeCl_3$ Tests

The experimental procedure was the same as that described in $FeCl_3$ tests. Development of a blue-green or black colour indicated the presence of tannins.

Bromine Water Tests

Bromine water (3%) was added to 1 mL of aqueous extraction solution. Formation of a precipitate indicated the presence of tannins.

Lead Acetate Tests

1 mL of lead acetate solution was added to 1 mL of aqueous extraction solution; a precipitate was considered evidence for the presence of tannins.

Lime Water Tests

Clear lime water (1 mL) was added to 1 mL of aqueous extraction solution. Formation of a precipitate indicated the presence of tannins.

Gelatin Tests

1 mL of aqueous extraction solution was mixed with 1 mL of 0.5% gelatin dissolved in 10% NaCl solution. Turbidity indicated the presence of tannins.

Tests for Flavonoids

Shinoda Tests

An appropriate amount of magnesium powder was added to 1 mL of methanol extraction solution, followed by two drops of HCl. Development of a red to red-purple colour indicated the presence of flavonoids.

Alkaline Reagent Tests

1 mL of methanol extraction solution was mixed with 1 mL of 2% NaOH solution. Development of an intense yellow colour followed by a change to colourless on addition of a few drops of diluted HCl indicated the presence of flavonoids.

$AlCl_3$ Tests

A few drops of methanol extraction solution were added onto a thin-layer chromatography plate. A 1% $AlCl_3$ methanol solution was sprayed onto the plate. Observation of yellow-green fluorescence under an ultraviolet lamp indicated the presence of flavonoids.

Lead Acetate Tests

A few drops of lead acetate solution were added to 1 mL of methanol extraction solution. Formation of a yellow precipitate indicated the presence of flavonoids.

Tests for Saponins

Foam Tests

1 mL of aqueous extraction solution was mixed with 5 mL of distilled water. This mixture was shaken and then left to stand for 10 min. Formation of a stable foam indicated the presence of saponins.

Tests for Steroids and Triterpenoids

Liebermann-Burchard Tests

5 mL of the aqueous extraction solution was placed in an evaporating dish and then evaporated. The residue was dissolved in 1 mL of acetic anhydride. One drop of H_2SO_4 was added and development of a red or purple colour indicated the presence of triterpenoids. Development of a blue-green colour indicated the presence of steroids.

Salkowski Tests

1 mL of methanol extraction solution was mixed with 1 mL of $CHCl_3$. Then, 1 mL of H_2SO_4 was added carefully, and the mixture was shaken gently. A reddish-brown colour in the $CHCl_3$ layer and green fluorescence in the H_2SO_4 layer indicated the presence of steroids or triterpenoids.

Tests for Terpenoids

$CHCl_3$-H_2SO_4 Tests

1 mL of methanol extraction solution was mixed with 2 mL of $CHCl_3$ and then evaporated. H_2SO_4 (2 mL) was added carefully, and the mixture was heated at 60°C for 2 min. Development of a grey colour indicated the presence of terpenoids.

Vanillin-H_2SO_4 Tests

A few drops of petroleum ether extraction solution were added onto a thin-layer chromatography plate. Chromogenic reagent was prepared by dissolving 5 g of vanillin in 100 mL of 10% H_2SO_4 ethanol solution, and then sprayed onto the plate. Development of a red, blue, or purple colour indicated the presence of volatile oils, terpenoids, and steroids.

Tests for Alkaloids

Bertrad's Reagent Tests

1 mL of methanol extraction solution was mixed with 1 mL of tungstosilicic acid reagent. The reagent was prepared by dissolving 5 g of tungstosilicic acid hydrate in 100 mL of distilled water and adding a small amount of HCl to adjust the pH to 2.0. Formation of a pale yellow or off-white precipitate indicated the presence of alkaloids.

Dragendorff's Reagent Tests

1 mL of methanol extraction solution was mixed with 1 mL of Dragendorff 's reagent. For the reagent, solution A (850 mg of bismuth subnitrate dissolved in 40 mL of distilled water and 10 mL of acetic acid) and solution B (8 g of KI dissolved in 20 mL of distilled water) were mixed in equal volumes to prepare a stock solution. A sample of this stock solution (10 mL) was then mixed with 20 mL of acetic acid and diluted to 100 mL with distilled water. Formation of a light yellow or reddish brown precipitate indicated the presence of alkaloids.

Mayer's Reagent Tests

1 mL of methanol extraction solution was mixed with 1 mL of Mayer's reagent. For the reagent, solution A (1358 mg of $HgCl_2$ dissolved in 60 mL of distilled water) and solution B (5 g of KI dissolved in 10 mL of distilled water) were mixed and then diluted to 100 mL with distilled water. Formation of a white or light yellow precipitate indicated the presence of alkaloids.

Tests for Anthraquinones

Borntrager's Tests

1 mL of methanol extraction solution was mixed with 1 mL of 10% NaOH solution. A red colour developed. Next, a small volume of 30% H_2O_2 solution was added and the mixture was heated at 60°C. HCl solution was then added and the red colour disappeared, finally, NaOH solution was added and development of a red colour indicated the presence of anthraquinones.

Magnesium Acetate Tests

Three drops of 1% magnesium acetate methanol solution were added to 1 mL of methanol extraction solution. Development of a red colour indicated the presence of anthraquinones.

Tests for Coumarins and Lactones

Hydroxamic Acid Iron Tests

Three drops of 7% hydroxylamine hydrochloride methanol solution and 10% KOH methanol solution were added to 1 mL of methanol extraction solution. After heating at 60°C, 5% HCl was added to adjust the pH to 3.0-4.0. Next, two drops of 1% $FeCl_3$ ethanol solution were added. Development of a orange or purple colour indicated the presence of coumarins and lactones.

Diazotization Tests

Methanol extraction solution was used. The experimental procedure was the same as that described in Diazotization tests. Development of a red colour indicated the presence of coumarins and lactones.

Fluorescence Tests

A few drops of methanol extraction solution were added onto a thin-layer chromatography plate and blue-green fluorescence was observed under ultraviolet lamp. 1% KOH solution was sprayed onto the plate. Generation of intense fluorescence indicated the presence of coumarins.

Tests for Volatile Oils and Fats

Phosphomolybdic Acid Tests

A few drops of petroleum ether extraction solution were added onto a thin-layer chromatography plate and 25% phosphomolybdic acid solution (2.5 g of phosphomolybdic acid hydrate dissolved in 10 mL of absolute ethanol) was sprayed onto the plate. Development of a blue colour indicated the presence of lipids, triterpenoids, and steroids.

Vanillin-H_2SO_4 Tests

Petroleum ether extraction solution was used. The experimental procedure was the same as that described in Vanillin-H_2SO_4 tests. Development of a red, blue, or purple colour indicated the presence of volatile oils, terpenoids, and steroids.

Sudan Tests

One drop of Sudan III solution (0.1 g of sudan III dissolved in 10 mL of 95% ethanol) was added to 1 mL of methanol extraction solution. Development of an orange colour indicated the presence of oils and fats. One drop of Sudan IV solution (0.01 g of Sudan IV dissolved in 5 mL of acetone, followed by addition of 5 mL of 70% ethanol) was added to 1 mL of methanol extraction solution. Development of a red colour indicated the presence of oils and fats.

Tests for Cardiac Glycosides

Kedde Tests

A few drops of methanol extraction solution were added onto a thin-layer chromatography plate. Chromogenic reagent was prepared by mixing solution A (2% methanol solution of 3,5-dinitrobenzoic acid) and solution B (2 M KOH solution) in equal volumes. The reagent was sprayed onto the plate. Development of a purple-red colour followed by a change to colourless indicated the presence of cardiac glycosides.

Raymond Tests

Methanol extract (1 mg) was dissolved in 50% ethanol. Both 2% *m*-dinitrobenzene ethanol solution (0.1 mL) and 20% NaOH solution (0.2 mL) were added. Development of a blue-purple colour indicated the presence of cardiac glycosides.

Legal Tests

Methanol extract (1 mg) was dissolved in two drops of pyridine. One drop of 3% sodium nitroprusside solution and one drop of 2 M NaOH solution were added. Development of a dark red colour followed by a change to colourless indicated the presence of cardiac glycosides.

Keller-Kilani Tests

1 mL ofmethanol extraction solution was mixed with acetic acid containing two drops of 2% $FeCl_3$ solution. Then the mixture was poured into another test tube containing 1 mL of H_2SO_4. A brown ring at the aqueous phase/organic phase interface indicated the presence of cardiac glycosides.

Tests for Cyanogenic Glycosides

Prussian Blue Tests

1 g of *R. ciliinervis* root powder was placed in a test tube, 2 mL of distilled water was added, and the test tube was immediately wrapped with filter paper. Then, one drop of 10% KOH solution was added onto the filter paper, and the system was heated at 60°C for 30 min. Next, one drop each of 10% ferrous sulphate, 10% HCl, and 5% $FeCl_3$ were sequentially added onto the filter paper. A blue colour on the filter paper indicated the presence of cyanogenic glycosides.

Preparation of Different Extracts of R. ciliinervis Root for Quantitative Phytochemical Analysis, Antioxidant Activity Assays and UHPLC-MS Analysis

The collected samples of *R. ciliinervis* root were dried in a cool ventilated place, and pulverized to powder. The powder (20 g) of *R. ciliinervis* root were added to a single-neck round-bottomed flask (glass, 500 mL), followed by addition of 200 mL of various solvents (water, methanol, ethanol or 80% ethanol) and refluxing using a hotplate magnetic stirrer employing methyl silicone oil as the heating medium for 6 h at the respective boiling points of the solvents. The same solvent is employed once for each individual extraction process. To minimize errors, three separate but parallel experiments were conducted, and the data collected from these experiments were subsequently utilized to describe the observed statistical differences. Extracts were filtered through a Whatman No.1 filter paper and evaporated under reduced pressure at < 50°C until dry using a rotary evaporator. All solvent extracts utilized in this experiment underwent rigorous drying to achieve a constant weight prior to their application, thereby ensuring minimal residual solvent content. All dried extracts were weighed and stored at −20°C until use. Yield was calculated as % yield = (weight of dry extract/initial weight of dry sample) × 100.

Quantitative Phytochemical Analysis

Determination of Ttotal Carbohydrate Content (TCC)

Briefly, 250 μL of *R. ciliinervis* root extract (0.2-5.0 mg/mL) in distilled water, 125 μL of phenol solution (5%), and 625 μL of H_2SO_4 were mixed in an Eppendorf tube and incubated for 30 min. Subsequently, 200 μL of the sample was pipetted from each Eppendorf tube onto a microplate. A calibration curve was produced based on glucose (0–200 mg/L) as a standard. The absorbance of the sample was recorded at 490 nm against a blank sample consisting of *R. ciliinervis* root extract with distilled water. The mean of three readings was used and TCC was expressed in milligrams of glucose equivalents (GE)/g of *R. ciliinervis* root extract.

Determination of Total Protein Content ($TP_{ro}C$)

Briefly, 200 μL of bicinchoninic acid (BCA) working solution and 20 μL of *R. ciliinervis* root extract (0.4-5.0 mg/mL) in distilled water were mixed in a microplate and incubated at 37°C for 30 min. A calibration curve was produced based on bovine serum albumin (BSA) (0–500 mg/L) as a standard. The absorbance of the sample was recorded at 562 nm against a blank sample consisting of *R. ciliinervis* root extract with distilled water. The mean of three readings was used and $TP_{ro}C$ was expressed in milligrams of BSA equivalents (BSAE)/g of *R. ciliinervis* root extract.

Determination of Total Phenolic Content ($TP_{he}C$)

Briefly, 100 μL of Folin & Ciocalteu's phenol reagent (FC reagent) (1 M) and 200 μL of *R. ciliinervis* root extract (0.4-5.0 mg/mL) in distilled water were mixed in an Eppendorf tube and incubated for 5 min. Subsequently, 500 μL of Na_2CO_3 solution (20%) was added and allowed to stand at room temperature for 40 min in the dark (with mixing every 10 min). Subsequently, 200 μL of the sample was pipetted from each Eppendorf tube onto a microplate. A calibration curve was produced based on gallic acid (0–100 mg/L) as a standard. The absorbance of the sample was recorded at 750 nm against a blank sample consisting of *R. ciliinervis* root extract with distilled water and Na_2CO_3. The mean of three readings was used and $TP_{he}C$ was expressed in milligrams of gallic acid equivalents (GAE)/g of *R. ciliinervis* root extract.

Determination of Total Steroid Content (TSC)

Briefly, 180 μL of *R. ciliinervis* root extract (0.1-2.0 mg/mL) in acetic anhydride and 20 μL of H_2SO_4 were mixed in a microplate and incubated for 10 min. A calibration curve was produced based on oleanolic acid (0–40 mg/L) as a standard. The absorbance of the sample was recorded at 350 nm against a blank sample consisting of *R. ciliinervis* root extract with acetic anhydride without H_2SO_4. The mean of three readings was used and TSC was expressed in milligrams of oleanolic acid equivalents (OAE)/g of *R. ciliinervis* root extract.

Determination of Total Alkaloid Content (TAC)

Briefly, 1 mL of *R. ciliinervis* root extract dissolved in 2N HCl at 1 mg/mL concentration was mixed with 5 mL of citrate buffer solution (pH 4.7, comprising 0.5M K_2HPO_4 and 0.2 M citric acid) and 5 mL of bromocresol green solution. After thorough mixing, 5 mL of chloroform was added, vigorously shaken, and left to settle at room temperature for 5 min. A separatory funnel was employed to isolate the chloroform layer, which was then dried by adding a suitable quantity of anhydrous sodium sulfate and allowed to stand at room temperature for 30 min. The absorbance of this layer was measured at 420 nm, utilizing a chloroform blank as the reference. A calibration curve was produced based on berberine hydrochloride (1.24–12.36 mg/L) as a standard. The mean of three readings was used and TAC was expressed in milligrams of berberine hydrochloride equivalents (BHE)/g of *R. ciliinervis* root extract.

Determination of Total Flavonoid Content (TFC)

Briefly, 100 μL of $AlCl_3$ (2%) in methanol and 100 μL of *R. ciliinervis* root extract (0.2-5.0 mg/mL) in methanol were mixed in a microplate and incubated at room temperature for 10 min. A calibration curve was produced based on quercetin (0–100 mg/L) as a standard. The absorbance of the sample was recorded at 415 nm against a blank sample consisting of *R. ciliinervis* root extract with methanol. The mean of three

readings was used and TFC was expressed in milligrams of quercetin equivalents (QE)/g of *R. ciliinervis* root extract.

Determination of Total Phenolic Acid Content (TPAC)

Briefly, 20 μL of *R. ciliinervis* root extract (0.4-5.0 mg/mL) in distilled water, 20 μL of Arnow reagent, 20 μL of HCl solution (0.1 M), 120 μL of distilled water and 20 μL of NaOH solution (1 M) were mixed in a microplate and recorded immediately at 490 nm against a blank sample (Arnow reagent was replaced with distilled water). A calibration curve was produced based on caffeic acid (0–100 mg/L) as a standard. The mean of three readings was used and TPAC was expressed in milligrams of caffeic acid equivalents (CAE)/g of *R. ciliinervis* root extract.

Determination of Total Tannin Content ($TT_{an}C$)

Briefly, 200 μL of FC reagent (1 M) and 200 μL of *R. ciliinervis* root extract (0.4-5.0 mg/mL) in distilled water were mixed in an Eppendorf tube and incubated for 5 min. Subsequently, 100 μL of Na_2CO_3 solution (20%) and 1500 μL of distilled water were added and allowed to stand at room temperature for 30 min in the dark (with mixing every 10 min). Subsequently, 200 μL of the sample was pipetted from each Eppendorf tube onto a microplate. A calibration curve was produced based on tannic acid (0–200 mg/L) as a standard. The absorbance of the sample was recorded at 725 nm against a blank sample consisting of *R. ciliinervis* root extract with distilled water and Na_2CO_3. The mean of three readings was used and $TT_{an}C$ was expressed in milligrams of tannic acid equivalents (TAE)/g of *R. ciliinervis* root extract.

Determination of Gallotannin Content (GC)

Briefly, 875 μL of *R. ciliinervis* root extract (0.4-5.0 mg/mL) in methanol and 375 μL of saturated KIO_3 solution were mixed in an Eppendorf tube and incubated at 15°C for 120 min. A calibration curve was produced based on gallic acid (0–400 mg/L) as a standard. The absorbance of the sample was recorded at 550 nm against a blank sample (KIO_3 was replaced with distilled water). The mean of three readings was used and GC was expressed in milligrams of GAE/g of *R. ciliinervis* root extract.

Determination of Condensed Tannin Content (CTC)

Briefly, 4 mg of phloroglucinol was added to 2 mL of *R. ciliinervis* root extract (0.4-5.0 mg/mL) in distilled water. Subsequently, 1 mL of HCl solution and 1 mL of formaldehyde solution were added and mixed in an Eppendorf tube and incubated at room temperature overnight. The precipitate was separated by filtration, the unprecipitated phenolics were measured in the filtrate according to the method of $TP_{he}C$.

Antioxidant Activity Assay

DPPH Assay

Briefly, 100 μL of *R. ciliinervis* root extract (0.625-10.0 mg/mL) in methanol and 100 μL of DPPH in methanol (50 μM) were mixed in a microplate and allowed to stand at room temperature for 20 min in the dark. The absorbance of the sample was recorded at 515 nm. The Half-maximal inhibitory concentration (IC_{50}) values were calculated and expressed as the mean ± standard deviation (SD) in μg/mL.

ABTS Assay

Briefly, 190 μL of diluted ABTS solution and 10 μL of *R. ciliinervis* root extract (0.625-10.0 mg/mL) in DMSO were mixed in a microplate and incubated for 20 min in the dark. The absorbance of the sample was recorded at 734 nm. The IC_{50} values were calculated and expressed as the mean ± SD in μg/mL.

Hydroxyl Radical Assay

Briefly, 50 μL of *R. ciliinervis* root extract (0.625-10.0 mg/mL) in DMSO, 50 μL of $FeSO_4$ solution (3 mM) and 50 μL of H_2O_2 solution (3 mM) were mixed in a microplate and incubated for 10 min. After then 50 μL of salicylic acid solution (6 mM) was added and incubated at room temperature for 30 min in the dark. The

absorbance of the sample was recorded at 492 nm. The scavenging activity was expressed as % scavenging rate and was calculated as follows:

$$\%scavenging=\left(1-\frac{\Delta A_{sample}-\Delta A_{control}}{\Delta A_{control}}\right)\times 100\%$$

Superoxide Radical Assay

Briefly, 45 μL of *R. ciliinervis* root extract (0.625-10.0 mg/mL) in DMSO (10 mg/mL), 15 μL of *p*-nitroblue tetrazolium chloride (NBT) in DMSO (1 mg/mL) and 150 μL of NaOH in DMSO (50 μM) were mixed in a microplate and the absorbance of the sample was recorded immediately at 560 nm against a blank sample (NBT was replaced with DMSO). Curcumin was used as a positive reference. The scavenging activity was expressed as % scavenging rate and was calculated as follows:

$$\%scavenging=\left(1-\frac{\Delta A_{sample}}{\Delta A_{control}}\right)\times 100\%$$

FRAP Assay

Briefly, 20 μL of *R. ciliinervis* root extract (0.1 mg/mL) in DMSO and 180 μL of FRAP reagent were mixed in a microplate and incubated at 37°C for 30 min in the dark. A calibration curve was produced based on $FeSO_4$ (0–600 mg/L) as a standard. The absorbance of the sample was recorded at 595 nm. The standard curve of ferrous ion is y=4.416x+0.087. Trolox was used as positive reference. The FRAP was expressed as the Trolox Equivalent Antioxidant Capacity ($TEAC_{FRAP}$).

CUPRAC Assay

Briefly, 20 μL of $CuCl_2$ solution (100 mM), 50 μL of neocuproine in 96% ethanol (7.5 mM), 50 μL of NH_4Ac solution, 20 μL of *R. ciliinervis* root extract (0.25 mg/mL) in DMSO, and 30 μL of distilled water were mixed in a microplate and incubated at 50°C for 20 min. This mixture was allowed to stand at room temperature for 10 min. The absorbance of the sample was recorded at 450 nm. Trolox was used as positive reference. The CUPRAC was expressed as the Trolox Equivalent Antioxidant Capacity ($TEAC_{CUPRAC}$).

Iron Chelating Assay

Briefly, 50 μL of *R. ciliinervis* root extract (0.625-10.0 mg/mL) in methanol, 110 μL of ultra-pure water, and 20 μL of $FeCl_2$ solution (0.5 mM) were mixed in a microplate and incubated for 5 min. Subsequently, 20 μL of ferrozine solution (2.5 mM) was added and incubated for 10 min. The absorbance was recorded at 562 nm against a blank sample (ferrozine solution was replaced with water). $EDTANa_2$ was used as a positive reference. The IC_{50} values were calculated and expressed as the mean ± SD in μg/mL.

Copper Chelating Assay

Briefly, 40 μL of *R. ciliinervis* root extract (0.625-10.0 mg/mL) in ultra-pure water, 140 μL of acetic acid-sodium acetate buffer solution (pH 6.0, 50 mM), and 10 μL of $CuSO_4$ solution (5 mM) were mixed in a microplate and incubated for 30 min. Subsequently, 10 μL of pyrocatechol violet solution (4 mM) was added and incubated for 30 min. The absorbance was recorded at 632 nm against a blank sample (pyrocatechol violet was replaced with water). $EDTANa_2$ was used as a positive reference. The IC_{50} values were calculated and expressed as the mean ± SD in μg/mL.

H_2O_2 Assay

Briefly, 70 μL of phenol solution (pH 7.0, 12 mM, in 84 mM phosphate buffer (PBS)), 20 μL of 4-aminoantipyrine solution (pH 7.0, 0.5 mM, in 84 mM PBS), 32 μL of H_2O_2 solution (pH 7.0, 0.7 mM, in 84 mM PBS), 8 μL of horseradish peroxidise (EC 1.11.1.7) solution (pH 7.0, 1 U/mL, in 84 mM PBS) and 70 μL of

R. *ciliinervis* root extract (0.625-10.0 mg/mL) (pH 7.0, in 84 mM PBS) were mixed in a microplate and the absorbance of the sample was recorded immediately at 504 nm against a blank sample (phenol solution was replaced with PBS). Gallic acid was used as a positive reference. The IC_{50} values were calculated and expressed as the mean ± SD in μg/mL.

Singlet Oxygen Assay

Briefly, 40 μL of *R. ciliinervis* root extract (0.625-10.0 mg/mL) (pH 7.4, in 45 mM PBS), 50 μL of *N*,*N*-dimethyl-4-nitrosoaniline (pH 7.4, 0.2 mM, in 45 mM PBS), 20 μL of histidine solution (pH 7.4, 0.1 mM, in 45 mM PBS), 20 μL of NaClO solution (pH 7.4, 0.1 mM, in 45 mM PBS), 20 μL of H_2O_2 (pH 7.4, 0.1 mM, in 45 mM PBS) and 50 μL of PBS (pH 7.4, 45 mM) were mixed in a microplate and allowed to stand at room temperature for 40 min. The absorbance of the sample was recorded at 440 nm against a blank sample (*R. ciliinervis* root extract was replaced with PBS). Ferulic acid was used as a positive reference. The IC_{50} values were calculated and expressed as the mean ± SD in μg/mL.

β-Carotene Bleaching Assay

Briefly, *β*-carotene solution was prepared by dissolving *β*-carotene (2 mg) in $CHCl_3$ (10 mL). Then, 2 mL of the solution was pipetted into a flask and vortex-mixed with linoleic acid (40 mg) and Tween 40 (400 mg). After the removal of $CHCl_3$, 100 mL of oxygenated ultrapure water was added, and the emulsion shaken vigorously. Aliquots (2.4 mL) of the emulsion were pipetted into different test tubes containing 0.1 mL of *R. ciliinervis* root extract (5 mg/mL) in methanol. Butylated hydroxytoluene (BHT), butyl hydroxyanisole (BHA) and tertiary butylhydroquinone (TBHQ) were used as positive controls. In the control group, *R. ciliinervis* root extract was replaced with methanol. When the sample was added to the emulsion, it was recorded as t = 0 min. The tubes were capped and placed in a water bath at 60°C. The absorbance was recorded at 470 nm every 15 min until 120 min. Antioxidant activity coefficient (AAC) was calculated according to the following equation:

$$AAC = \frac{A_{A(120)} - A_{C(120)}}{A_{C(0)} - A_{C(120)}} \times 1000$$

where $A_{A(120)}$ is the absorbance of the antioxidant at 120 min, $A_{C(120)}$ is the absorbance of the control at 120 min, and $A_{C(0)}$ is the absorbance of the control at 0 min.

UHPLC-MS

Methanol extract of *R. ciliinervis* root was analyzed using UHPLC (Agilent 1290 system) with Q-TOF-MS (Agilent 6545 system). A ZORBAX SB-C_{18} column (150 × 3.0 mm, 1.8 μm; Agilent) was used. The column temperature was set to 40°C. The mobile phase was a mixture of 0.1% formic acid in water (solvent A) and a mixture of 0.1% formic acid in acetonitrile (solvent B) at a flow rate of 0.4 mL/min. Linear gradient elution was applied (0–1 min, 95% A; 1–30 min, 95–70% A; 30–50 min, 70–30% A; 50–56 min, 30–1% A; 56–60 min, 1% A). The extract was diluted to 1 mg/mL with methanol and filtered using a 0.22 μm membrane before use. The sample injection volume was 5 μL. The Q-TOF-MS (Agilent) was operated in positive-ion mode with scan range *m*/*z* 100–1700. Data were recorded and analyzed with qualitative analysis software (version B. 07.00, Agilent).

Stability Studies of Methanol Extract of R. ciliinervis Root

pH Stability

The stability in acidic and basic environments was investigated using a methanol extract of *R. ciliinervis* root dissolved in deionized water with the pH adjusted to 1, 3, 5, 7, 9, or 11 using 1 M HCl or 1 M NaOH. The final concentration of methanol extract was 5 mg/mL. After incubation at room temperature for 1 h, the pH of the mixture was adjusted to 7 and the $TP_{he}C$ and the ABTS scavenging abilities were examined.

Thermal Stability

To evaluate the thermal stability, methanol extract of *R. ciliinervis* root dissolved in deionized water (5 mg/mL, pH 7) was placed in test tubes with screw caps. The test tubes were placed in a boiling water bath (100°C). Samples were removed after 0, 15, 30, 60, 120, 180, and 240 min and cooled in an ice-water bath. and the $TP_{he}C$ and the ABTS scavenging abilities were examined.

Modeling of the Stability in the Gastrointestinal Tract

100 mL of methanol extract of *R. ciliinervis* root in distilled water (5 mg/mL) were mixed with 10 mL of PBS (pH 6.8, 10 mM) and incubated at 37 °C for 2 min (oral condition). Then 0.5 mL of 1 M HCl-KCl buffer (pH 1.5) and 5 mL of pepsin solution (pH 1.5, 32 U/mL in 1 M HCl-KCl buffer) were added to samples. The mixtures incubated at 37°C for 60 min (stomach condition). Thereafter, 1 mL of 1 M $NaHCO_3$ together with 1 mL of a mixture of bile and pancreatic juice (pH 8.2, 10 mg/mL of pancreatin, 14,600 U/mL of trypsin, and 13.5 mg/mL of bile extract in 10 mM PBS) was added to the mixture, and the pH was adjusted to 6.8. The mixtures were incubated at 37°C for 3 h (duodenal condition). The results were used for the determination of $TP_{he}C$ and ABTS scavenging abilities of methanol extract during simulated gastrointestinal digestion and were taken at 0, 0.5, 1-4 h.

Oxidative Stability of the Oils

Extra virgin olive oil (EVOO) and cold-pressed sunflower oil (CPSO) were placed in separate flasks. Methanol extract of *R. ciliinervis* root was added to the EVOO and CPSO flasks at concentrations of 100 and 25 μg/g, respectively. To compare with the stabilizing effect of methanol extract, EVOO and CPSO were supplemented with synthetic antioxidants TBHQ and BHT at 20 μg/g. A control group was prepared without antioxidants. The flasks were left open and placed in an oil bath at 160°C to simulate frying. Two samples from each category were removed from the flasks every 4 h for duplicate analysis. The oxidative stability of the oils was evaluated by measurement of the free acidity (percentage of oleic acid), peroxide values (milliequivalents of O_2/kg oil), and ultraviolet absorption at 232 and 270 nm (K_{232} and K_{270}).

Oral Acute Toxicity Study

Twenty adult Kunming mice (19–22 g) were acquired by Liaoning Changsheng Biotechnology Co., Ltd. (animal license number SCXK (Liao) 2020–0001; Liaoning, China). Housed mice had free access to food and water under a 12 h light–dark cycle. All mice were reared adaptively for 3 d before starting the experiment. We followed the relevant policies in the Guidelines for the Use of Laboratory Animals developed by Tonghua Normal University. The Institutional Animal Care and Use Committee of Tonghua Normal University approved the experimental protocol (Ethic approval code: 20240035) and the protocol follows the rules of the Declaration of Helsinki. The mice were divided into two groups (n = 20) with ten males and ten females in each group. The mice in the healthy control group received vehicle treatment. The methanol extract of *R. ciliinervis* root was dissolved in water to a final volume of 10 mL/kg mouse body weight (BW) in a single dose of 2000 mg/kg methanol extract. The mice were then continuously observed for 1 h for behavioral changes and toxicity. Intermittent observations were made for next 6 h, and a final observation was conducted at 24 h. At this stage, the survival rate was calculated and we found that no mice died. All mice were euthanized using isoflurane. On the basis of the study results, two doses (150 and 300 mg/kg) were selected for further study.

Hepatoprotective Experiments

Animals

Adult male Wistar rats (170–200 g) were acquired by Liaoning Changsheng Biotechnology Co., Ltd. (animal license number SCXK (Liao) 2020–0001; Liaoning, China). Housed rats had free access to food and water under a 12 h light–dark cycle. All rats were reared adaptively for 1 week before starting the experiment. We followed the relevant policies in the Guidelines for the Use of Laboratory Animals developed by Tonghua Normal University. The Institutional Animal Care and Use Committee of Tonghua Normal University approved the experimental protocol (Ethic approval code: 20240035) and the experimental protocol follows the rules of the Declaration of Helsinki.

Experimental Protocol

All the treatments were performed daily for 7 d. After the last day of treatment, rats in G2–G5 received *D*-galactosamine (700 mg/kg BW) by intraperitoneal injection. The rats were then fasted for 24 h with access to water. After all the above processes were completed, we found that no rats died after *D*-galactosamine injection. Next, all rats were anesthetized by intraperitoneal injection of pentobarbital sodium (50 mg/kg BW). Abdominal aortic blood was collected, and the hepatic tissue was rapidly excised. After leaving the blood samples at room temperature for 0.5 h, they were centrifuged for 0.25 h at 3000 rpm and 4°C. The serum was stored at −80°C. The tissue was washed with normal saline, dried using filter paper, and weighed. A 10% homogenate of the hepatic tissue was prepared using normal saline, centrifuged for 10 min at 10000 rpm and 4°C, and the supernatant was stored at −80°C.

Histopathological examination

The hepatic tissues of three rats were selected from each group, fixed with 4% paraformaldehyde, dehydrated and washed, and embedded in paraffin. After cutting into 4-μm thick sections, hematoxylin and eosin were used for staining. Observations were made using a light microscope. A digital camera was used to record histopathological changes.

Biochemical analyses

Before anesthesia, each rat was weighed. The hepatic tissue weight and BW of each rat were used to calculate the viscera index (VI) as follows: VI = viscera weight (g)/BW (g) × 100%. For assessment of biochemical parameters related to liver function, serum samples were analyzed for alanine aminotransferase (ALT), aspartate aminotransferase (AST), *γ*-glutamyl transpeptidase (*γ*-GT), malondialdehyde (MDA), catalase (CAT) and albumin. All samples were analyzed using commercial kits according to the manufacturer's guidelines.

Table S1. Qualitative phytochemical analysis of *R. ciliinervis* root.

No.	Phytochemicals	Types of tests	Sample Solution		
			Water	Methanol	Petroleum Ether
1	Proteins/Amino acids	1. Ninhydrin test	+	○	○
		2. Biuret test	+	○	○
2	Carbohydrates	1. Fehling's test	+	○	○
		2. Benedict's test	+	○	○
		3. Molisch's test	+	○	○
		4. Iodine tests	+	○	○
3	Phenolics	1. $FeCl_3$ test	+	○	○
		2. $FeCl_3$-$K_3[Fe(CN)_6]$ test	+	○	○
		3. Diazotization test	+	○	○
4	Organic acids	1. pH tests	+	○	○
		2. Blue litmus paper test	+	○	○
		3. Bromocresol green test	+	○	○
5	Tannins	1. $FeCl_3$ test	+	○	○
		2. Bromine water test	+	○	○
		3. Lead acetate test	+	○	○
		4. Lime water test	+	○	○
		5. Gelatin tests	+	○	○
6	Flavonoids	1. Shinoda test	○	+	◦
		2. Alkaline reagent test	○	–	○
		3. $AlCl_3$ test	○	–	○
		4. Lead acetate test	○	+	○
7	Saponins	1. Foam test	–	○	○
8	Steroids and triterpenoids	1. Liebermann–Burchard test	○	+	○
		2. Salkowski test	○	+	○
9	Terpenoids	1. $CHCl_3$-H_2SO_4 test	○	○	+
		2. Vanillin-H_2SO_4 test	○	○	+
10	Alkaloids	1. Bertrad's reagent	○	–	○
		2. Dragendorff's reagent	○	–	○
		3. Mayer's reagent	○	–	○
11	Anthraquinones	1. Borntrager's test	○	+	○
		2. Magnesium acetate test	○	+	◦

12	Coumarins and lactones	1. Hydroxamic acid iron test	○	+	◦
		2. Diazotization test	○	+	◦
		3. Fluorescence test	○	–	◦
13	Volatile oils and fats	1. Phosphomolybdic acid test	○	○	+
		2. Vanillin-H_2SO_4 test	○	○	+
		3. Sudan test III	○	○	+
		4. Sudan test IV	○	○	+
14	Cardiac glycosides	1. Kedde test	○	+	○
		2. Legal test	○	+	○
		3. Keller-kilani test	◦	+	○
15	Cyanogenic glycosides	1. Prussian blue test	–	◦	○

(+) indicates presence; (–) indicates absence; (○) indicates no test.

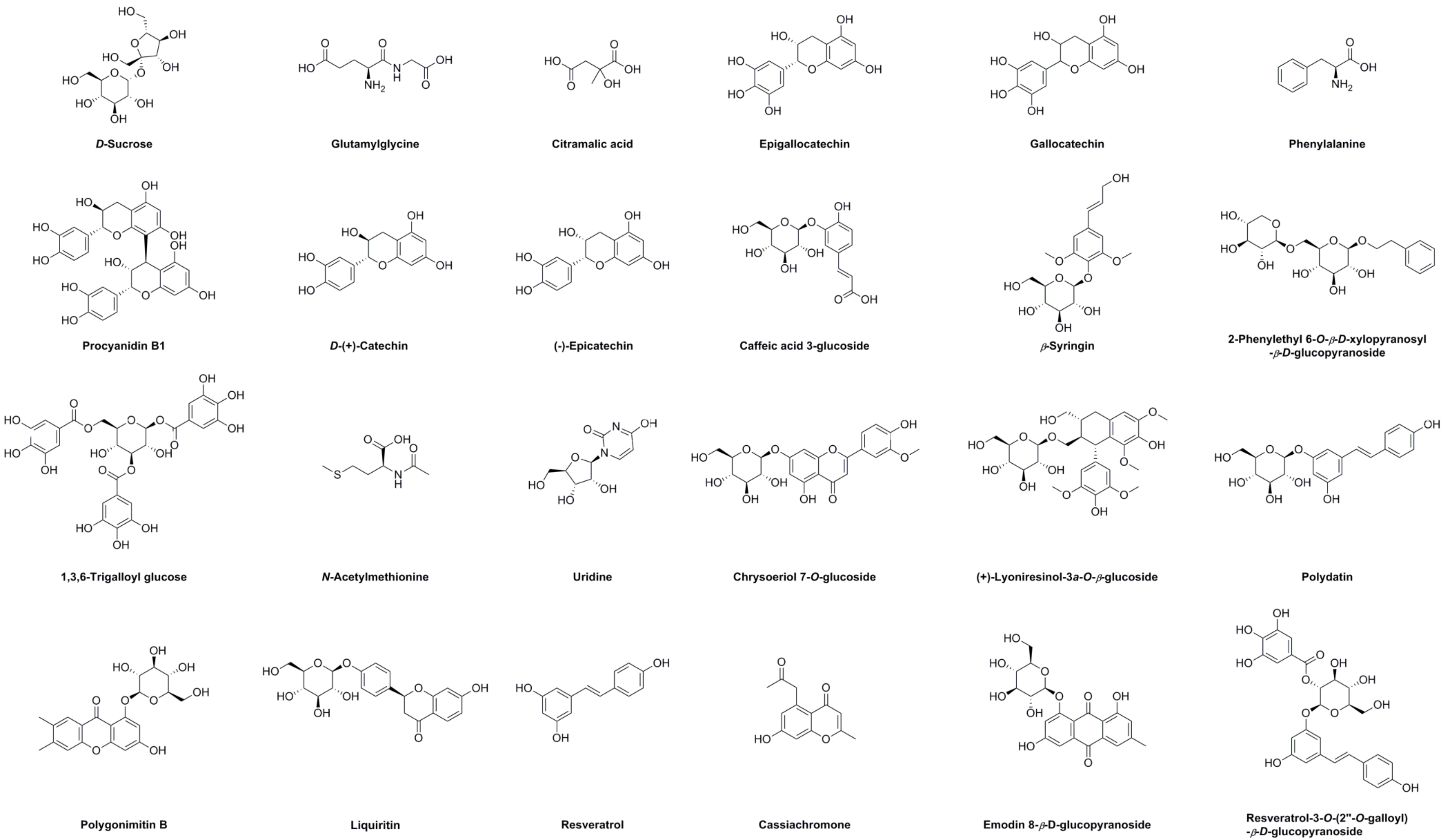

D-Sucrose
Glutamylglycine
Citramalic acid
Epigallocatechin
Gallocatechin
Phenylalanine
Procyanidin B1
D-(+)-Catechin
(-)-Epicatechin
Caffeic acid 3-glucoside
β-Syringin
2-Phenylethyl 6-*O*-*β*-*D*-xylopyranosyl -*β*-*D*-glucopyranoside
1,3,6-Trigalloyl glucose
N-Acetylmethionine
Uridine
Chrysoeriol 7-*O*-glucoside
(+)-Lyoniresinol-3*a*-*O*-*β*-glucoside
Polydatin
Polygonimitin B
Liquiritin
Resveratrol
Cassiachromone
Emodin 8-*β*-D-glucopyranoside
Resveratrol-3-*O*-(2''-*O*-galloyl) -*β*-*D*-glucopyranoside

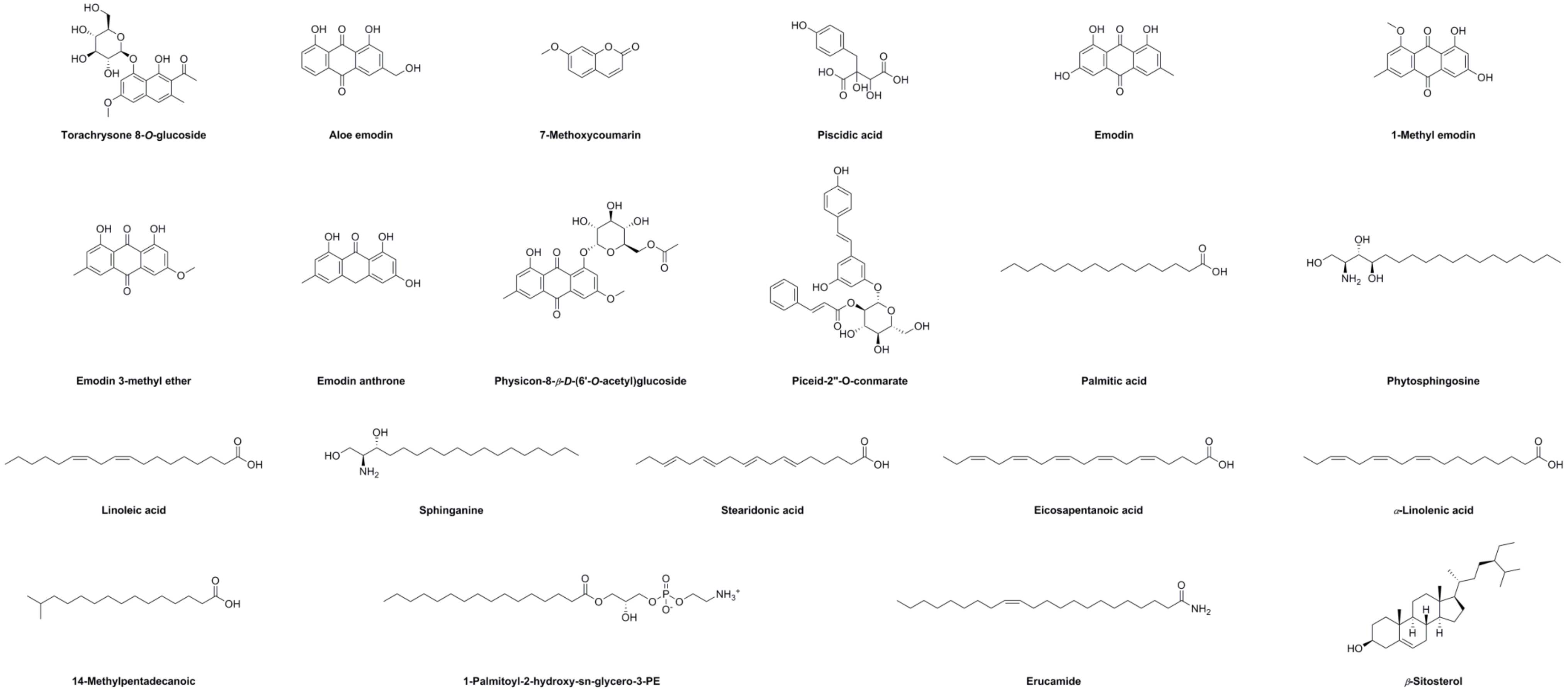

Figure S1. Chemical structures of the compounds identified in methanol extract of *R. ciliinervis* root.

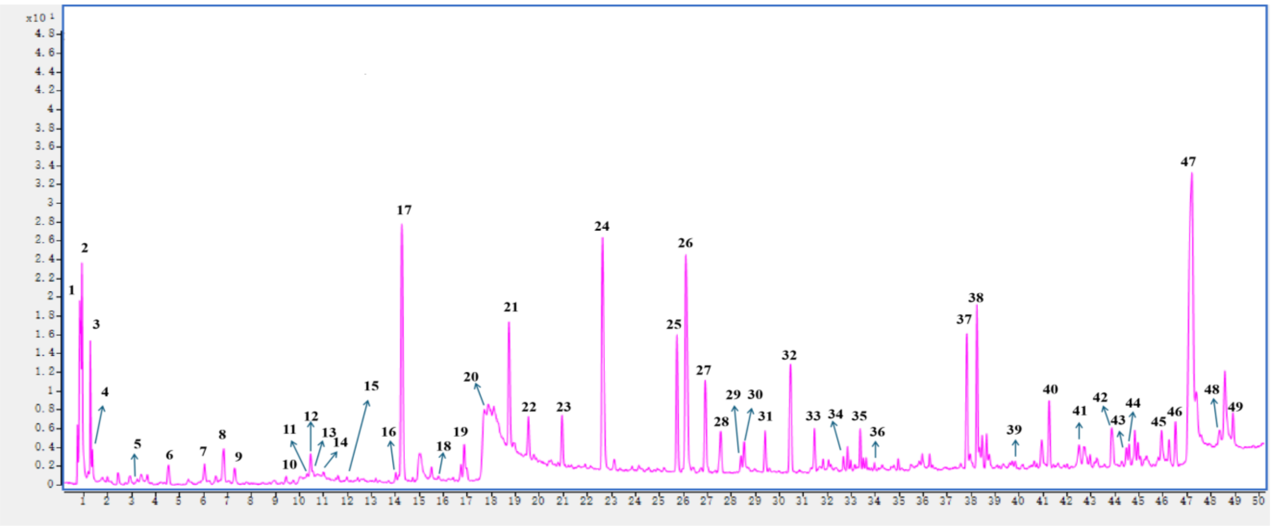

Figure S2. Positive-ion mode UHPLC-MS findings of methanol extract of *R. ciliinervis* root.

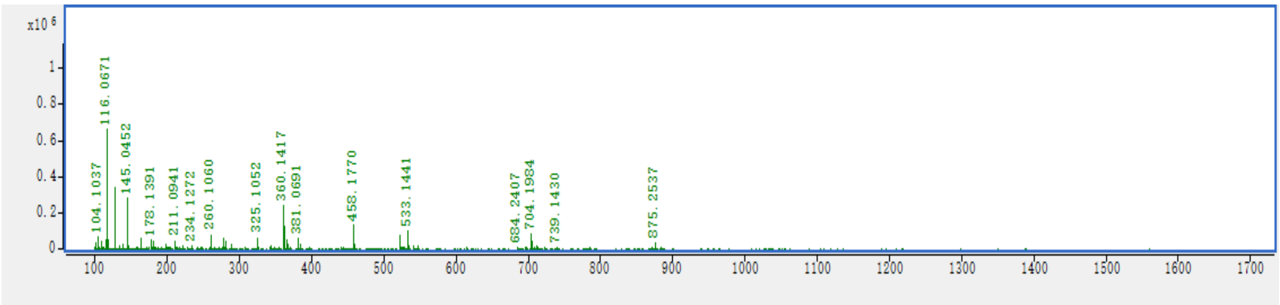

Figure S3. MS spectrum of peak 1

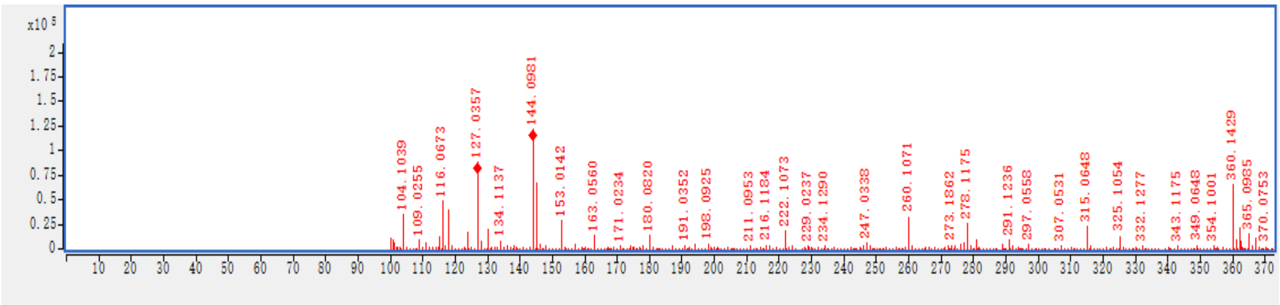

Figure S4. MS/MS spectrum of peak 1

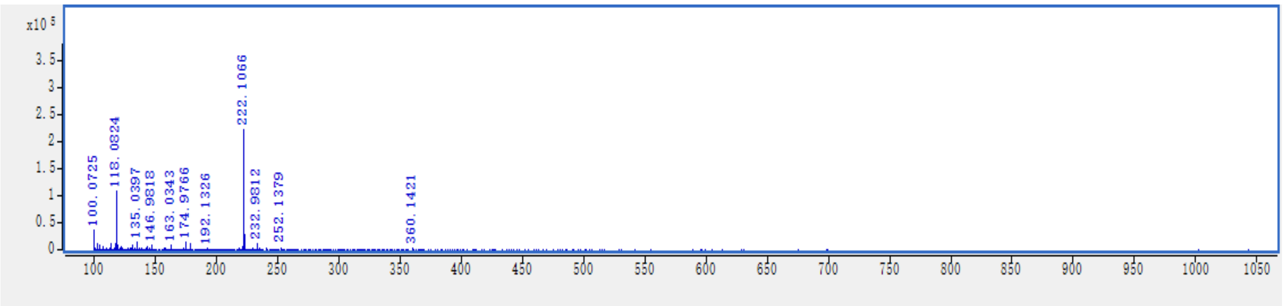

Figure S5. MS spectrum of peak 2

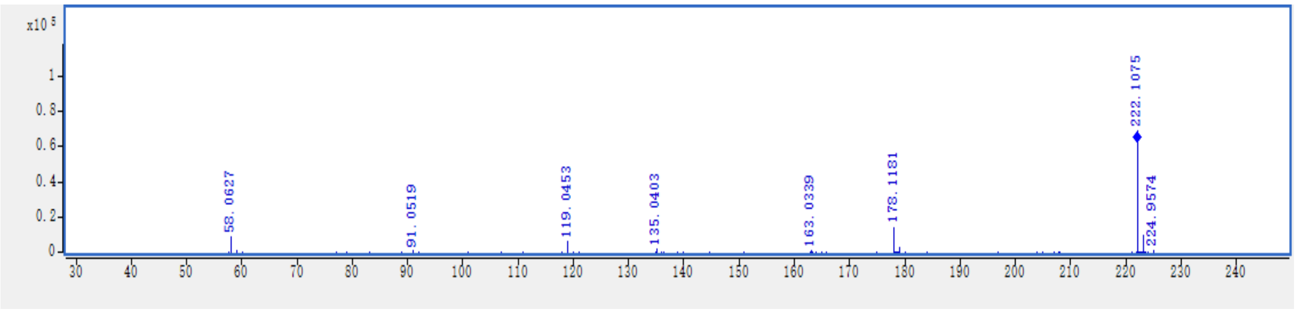

Figure S6. MS/MS spectrum of peak 2

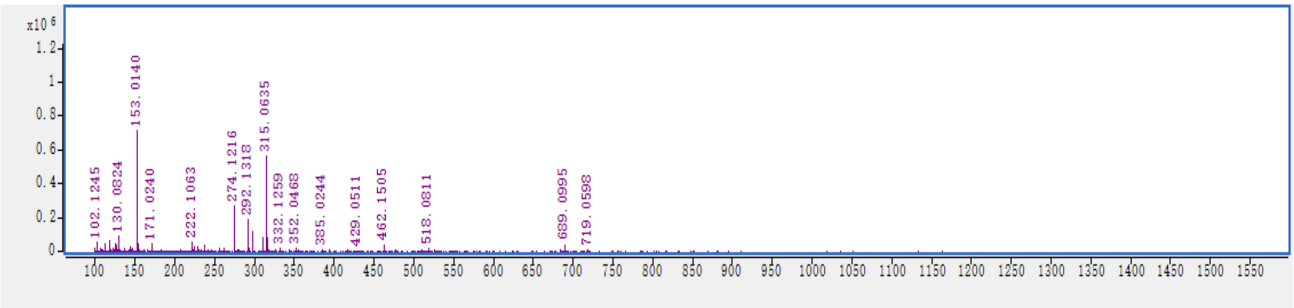

Figure S7. MS spectrum of peak 3

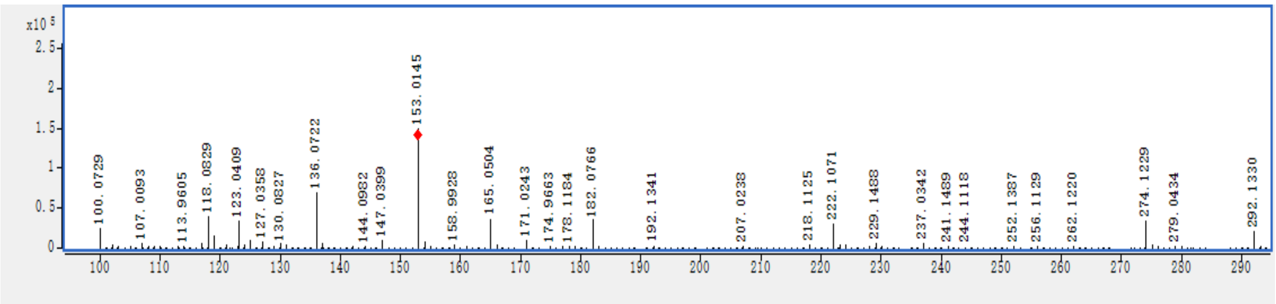

Figure S8. MS/MS spectrum of peak 3

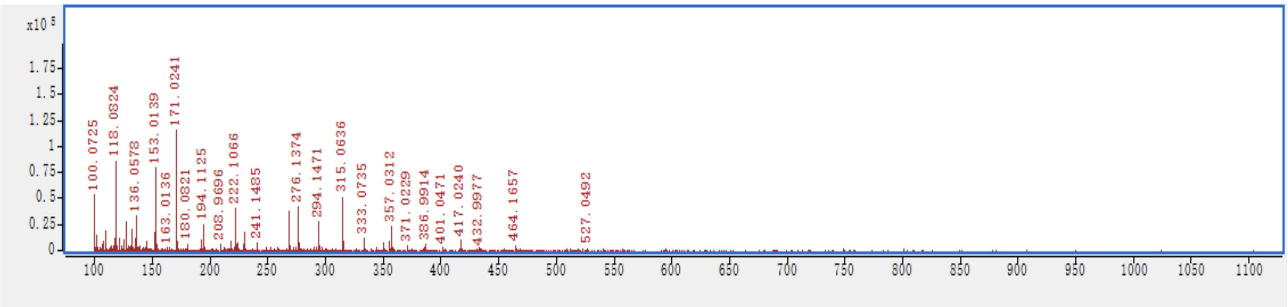

Figure S9. MS spectrum of peak 4

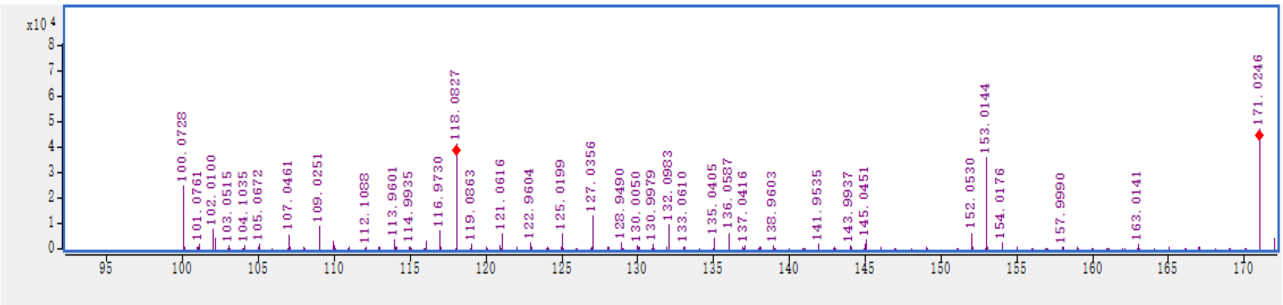

Figure S10. MS/MS spectrum of peak 4

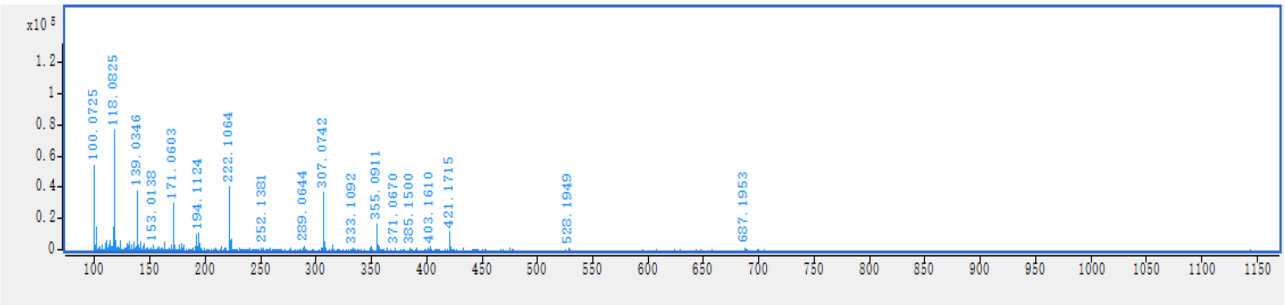

Figure S11. MS spectrum of peak 5

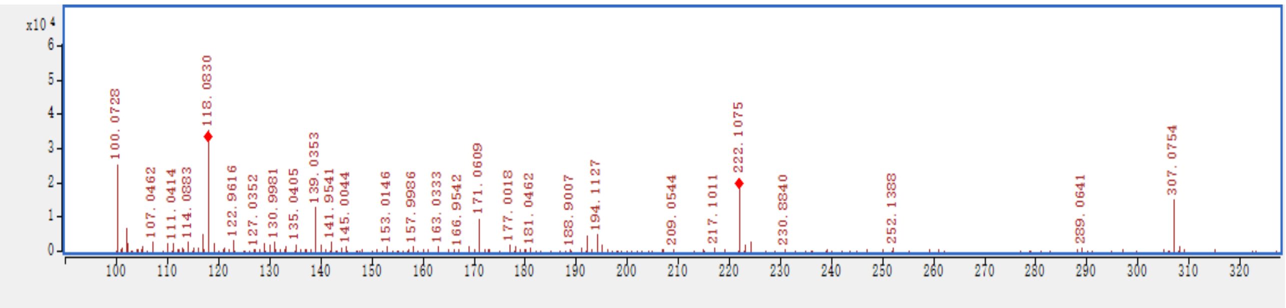

Figure S12. MS/MS spectrum of peak 5

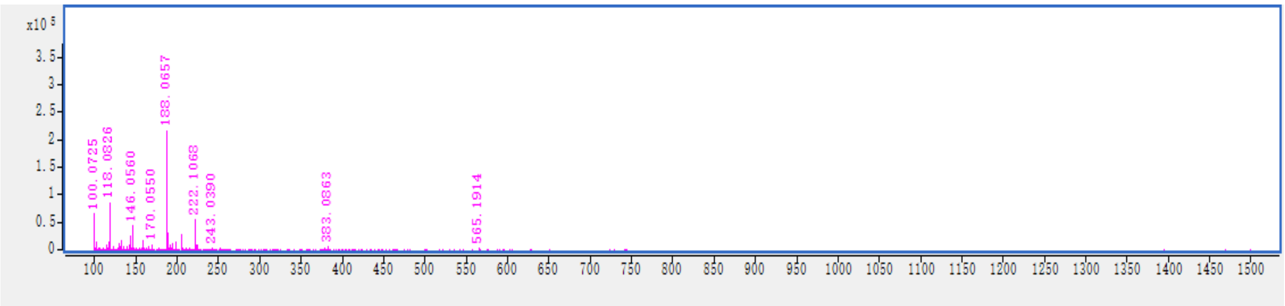

Figure S13. MS spectrum of peak 6

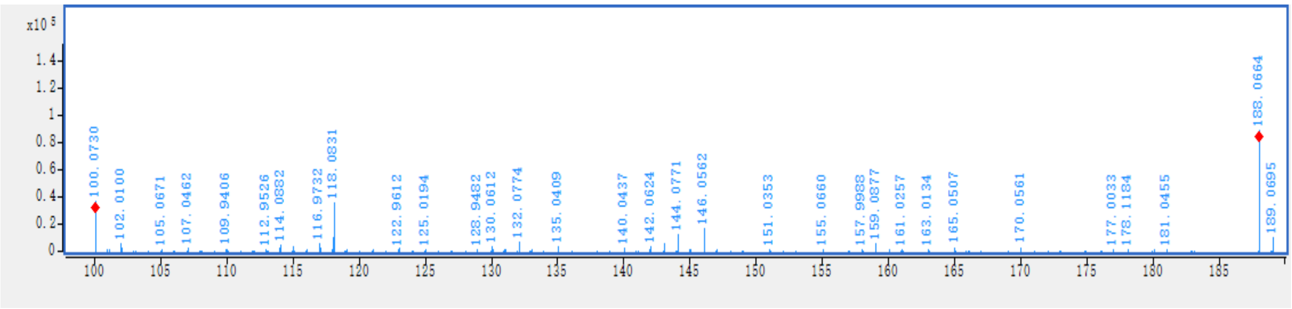

Figure S14. MS/MS spectrum of peak 6

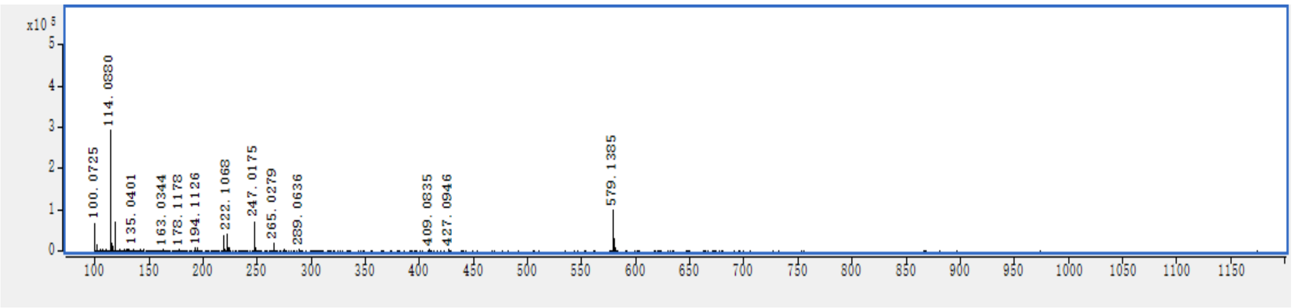

Figure S15. MS spectrum of peak 7

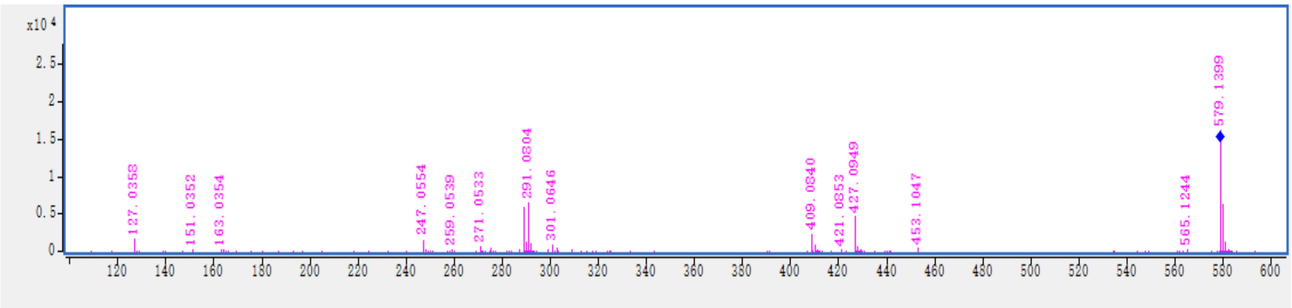

Figure S16. MS/MS spectrum of peak 7

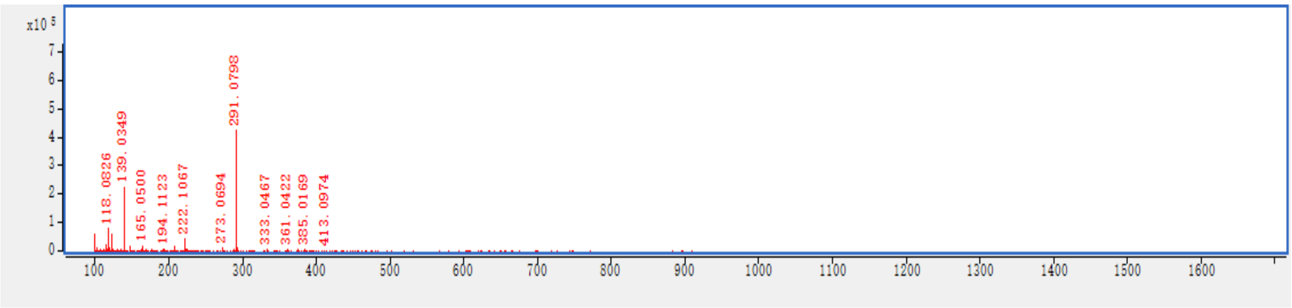

Figure S17. MS spectrum of peak 8

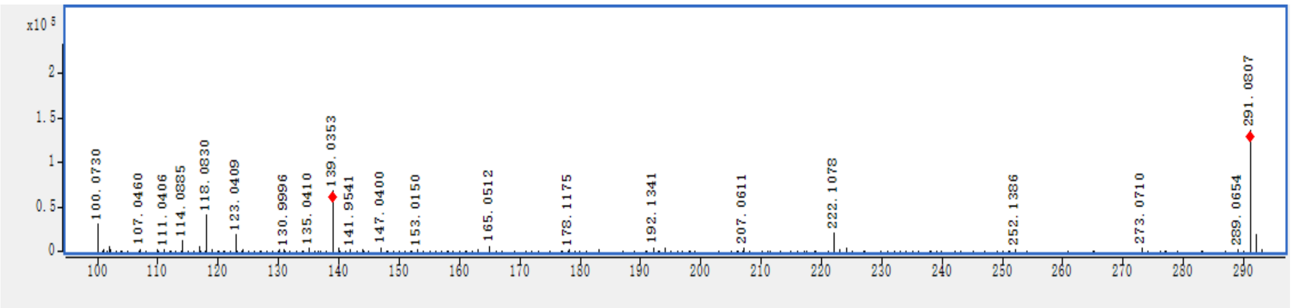

Figure S18. MS/MS spectrum of peak 8

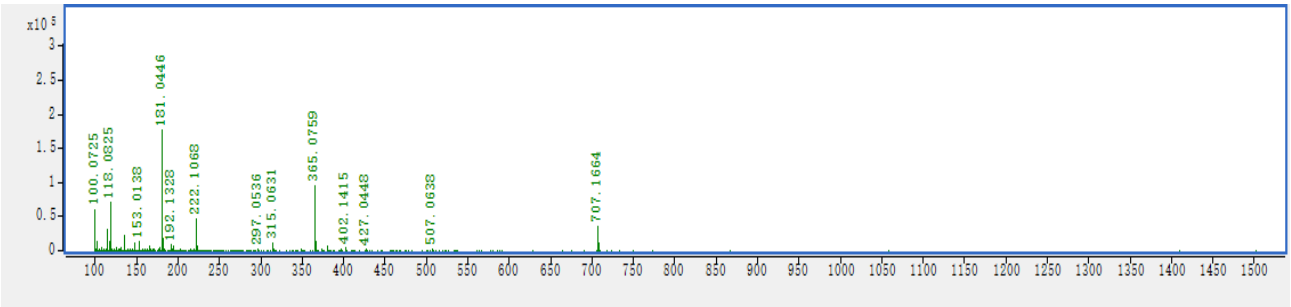

Figure S19. MS spectrum of peak 9

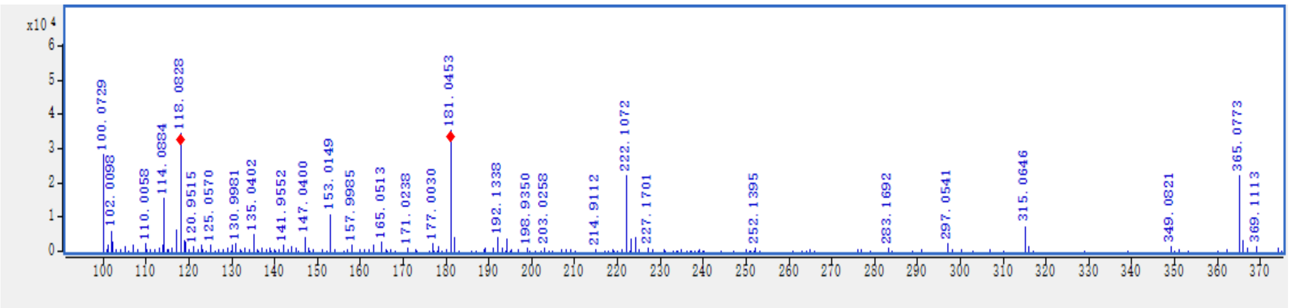

Figure S20. MS/MS spectrum of peak 9

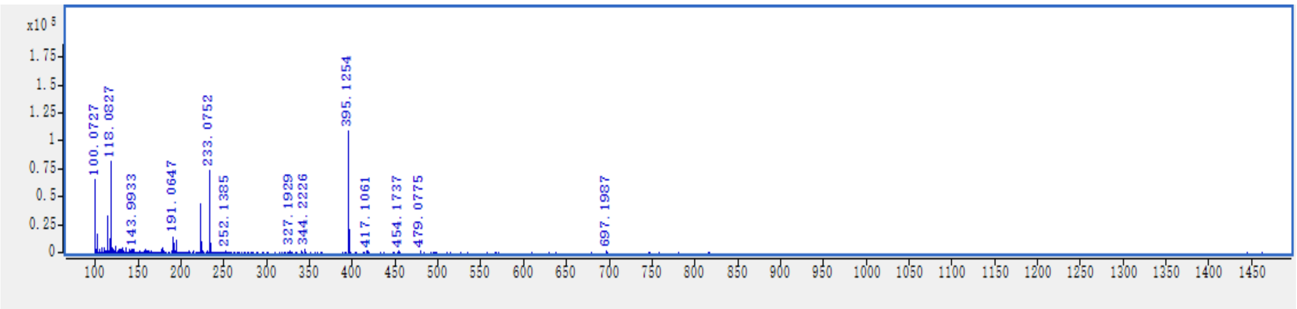

Figure S21. MS spectrum of peak 10

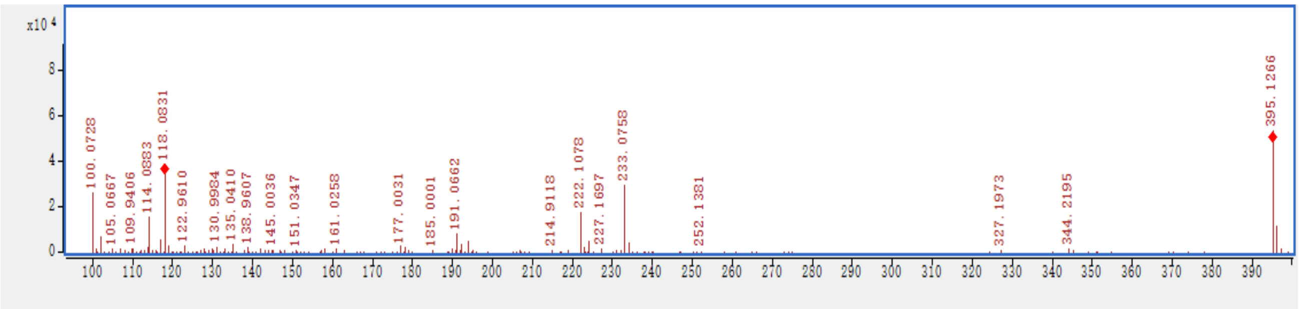

Figure S22. MS/MS spectrum of peak 10

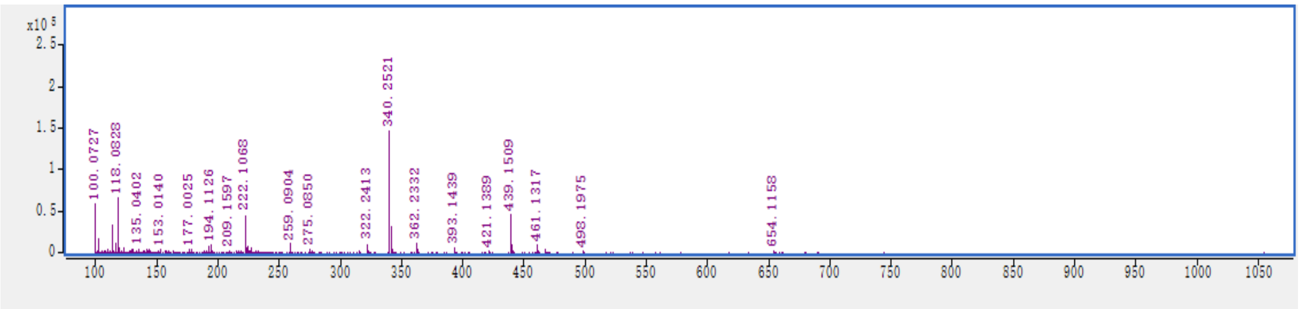

Figure S23. MS spectrum of peak 11

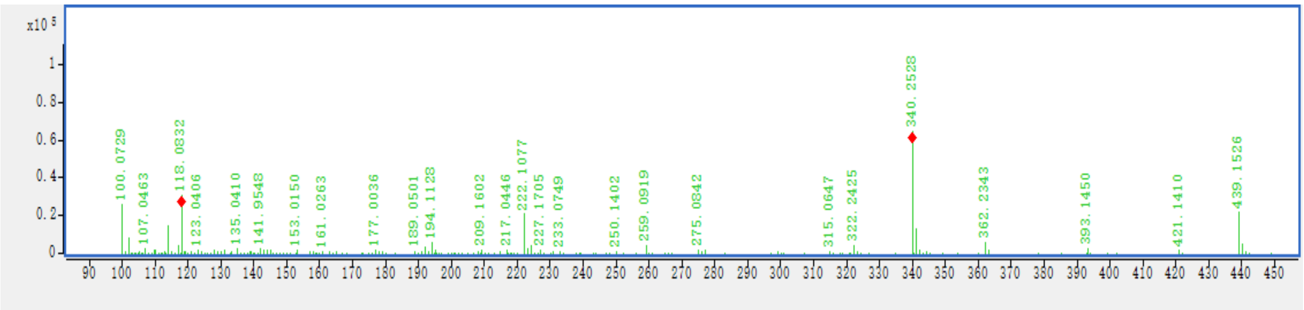

Figure S24. MS/MS spectrum of peak 11

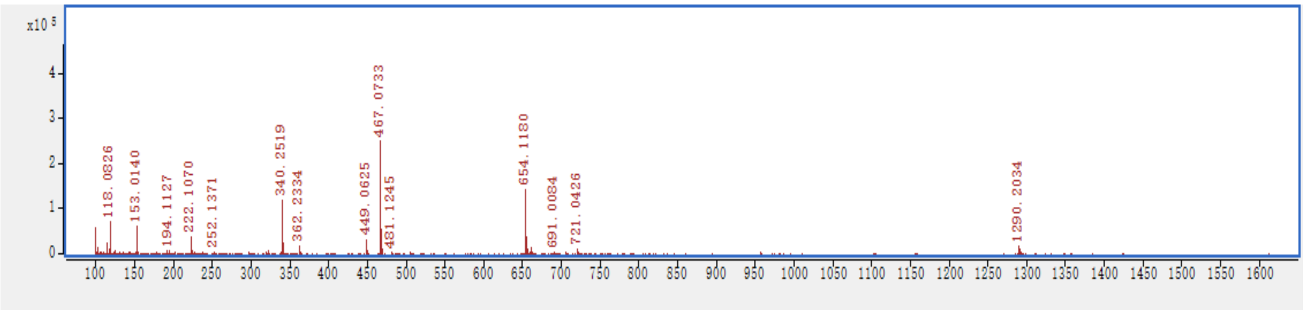

Figure S25. MS spectrum of peak 12

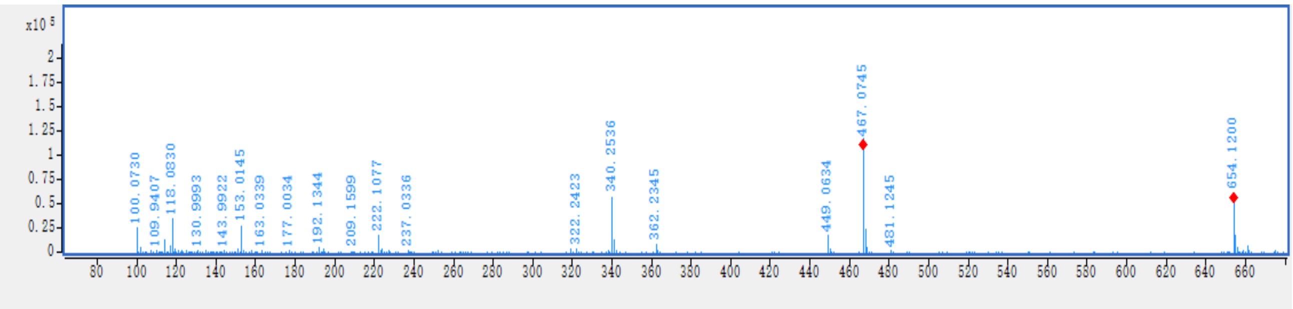

Figure S26. MS/MS spectrum of peak 12

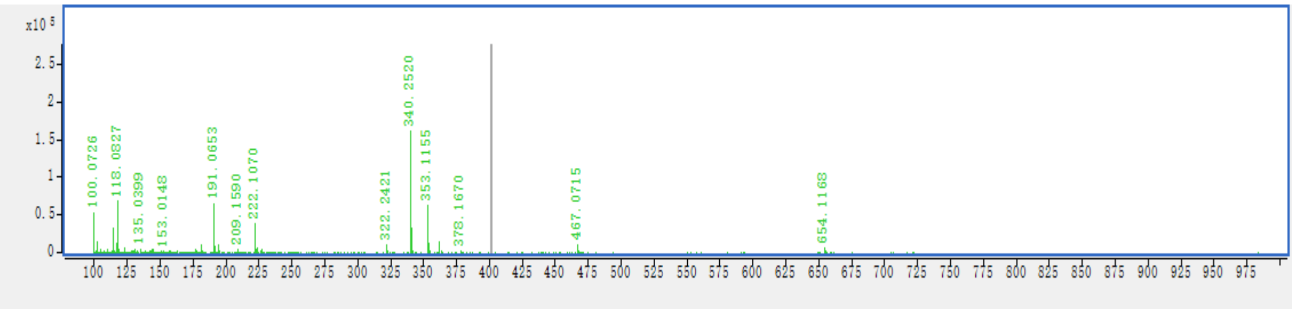

Figure S27. MS spectrum of peak 13

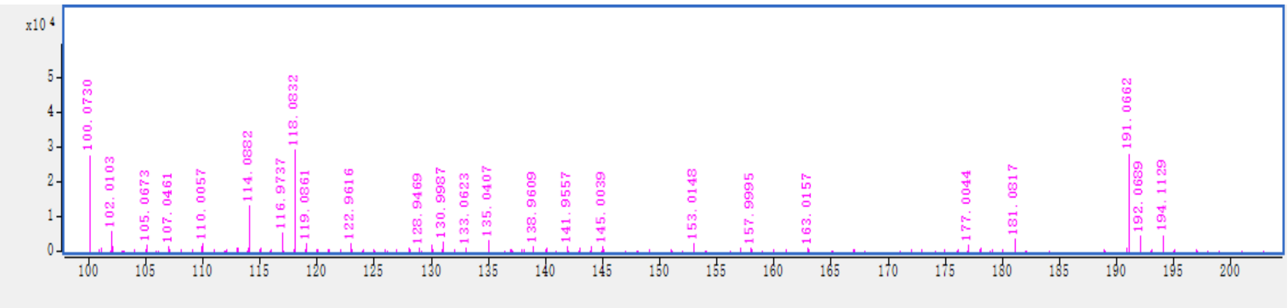

Figure S28. MS/MS spectrum of peak 13

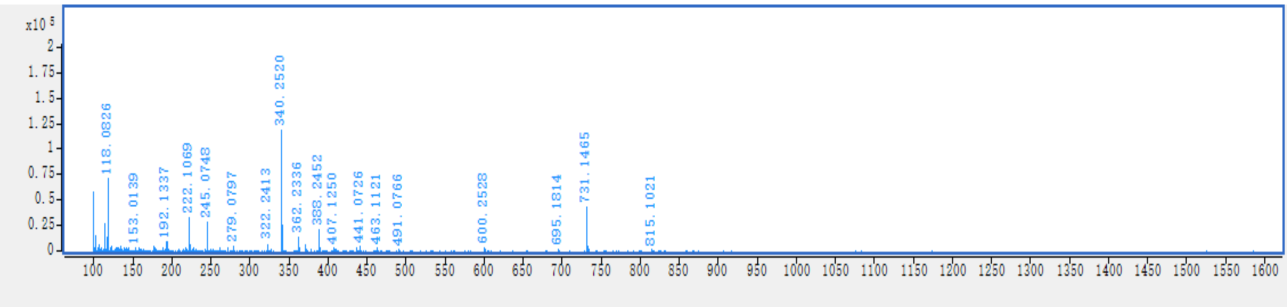

Figure S29. MS spectrum of peak 14

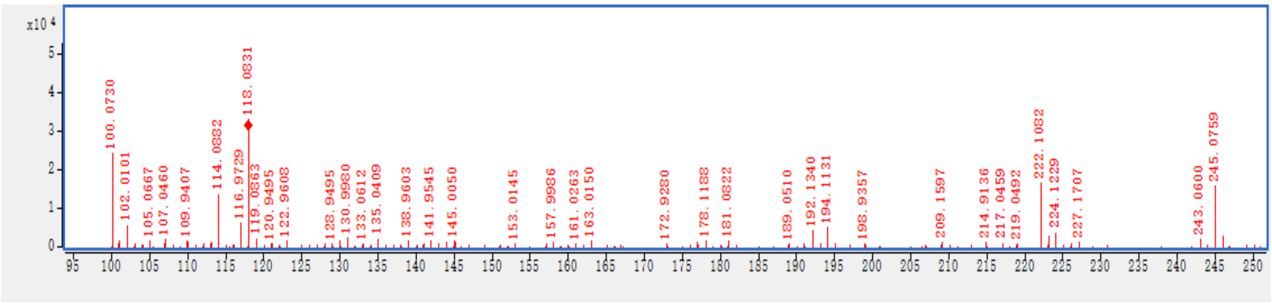

Figure S30. MS/MS spectrum of peak 14

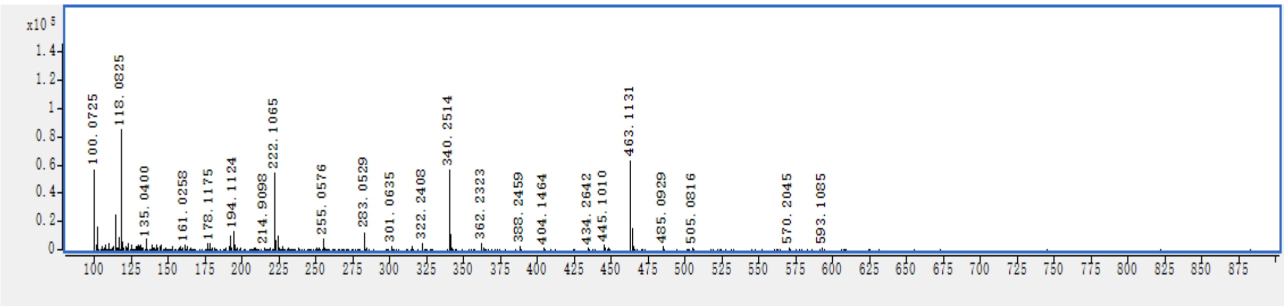

Figure S31. MS spectrum of peak 15

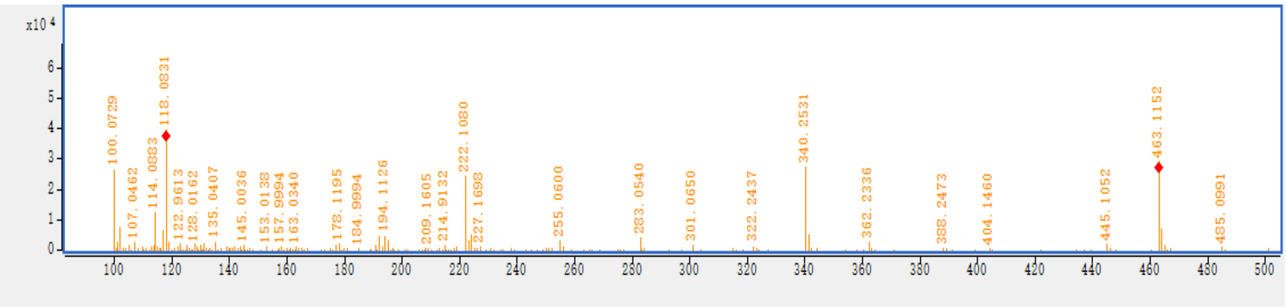

Figure S32. MS/MS spectrum of peak 15

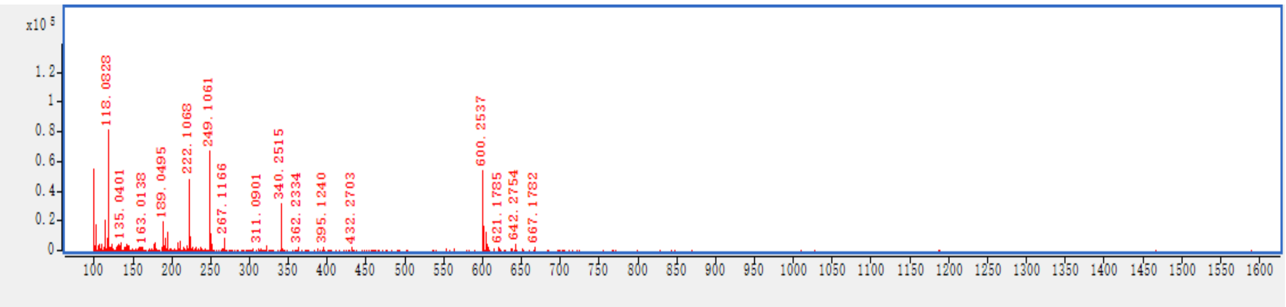

Figure S33. MS spectrum of peak 16

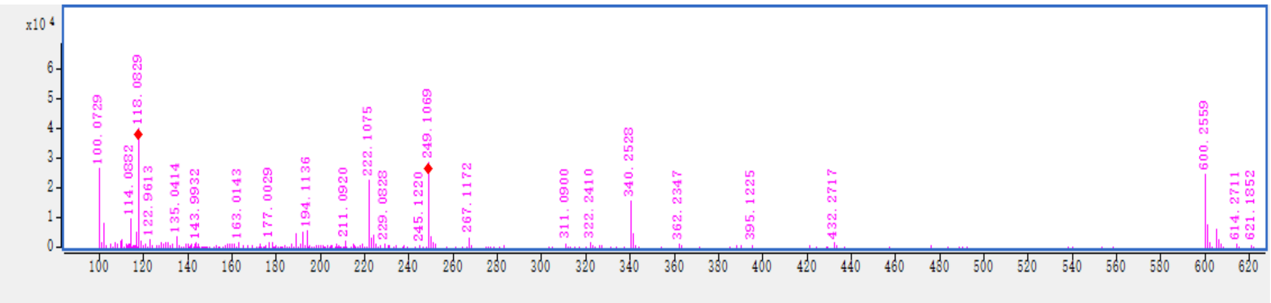

Figure S34. MS/MS spectrum of peak 16

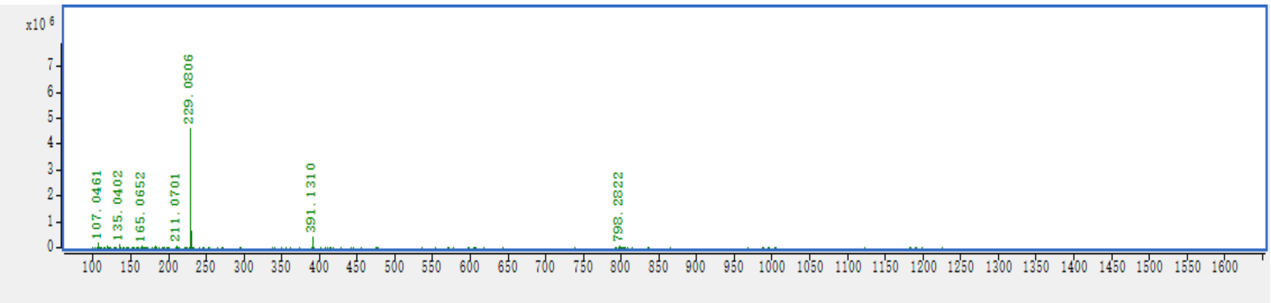

Figure S35. MS spectrum of peak 17

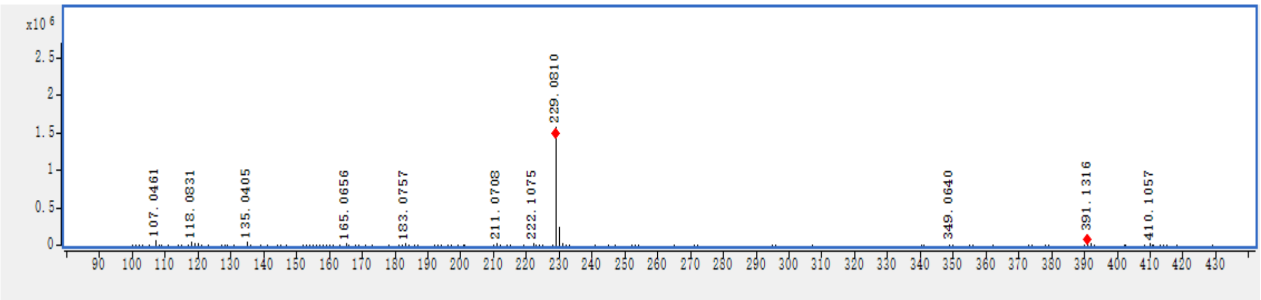

Figure S36. MS/MS spectrum of peak 17

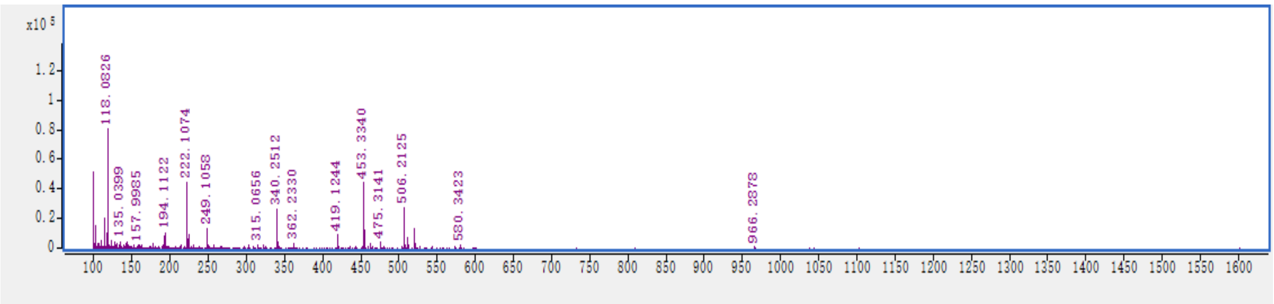

Figure S37. MS spectrum of peak 18

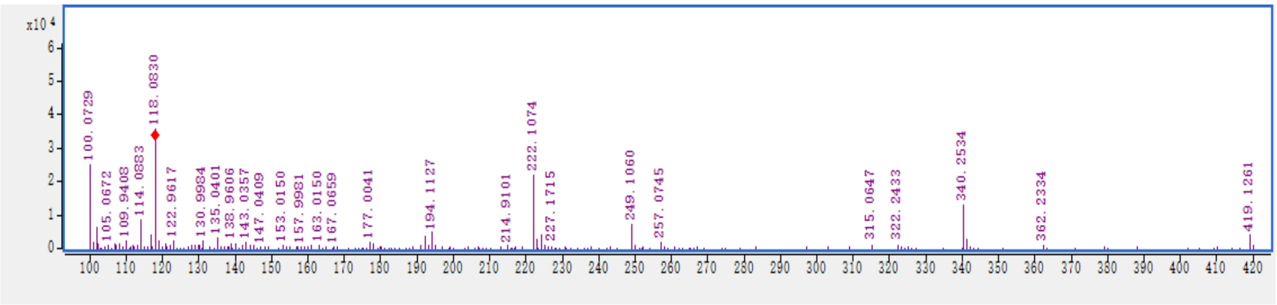

Figure S38. MS/MS spectrum of peak 18

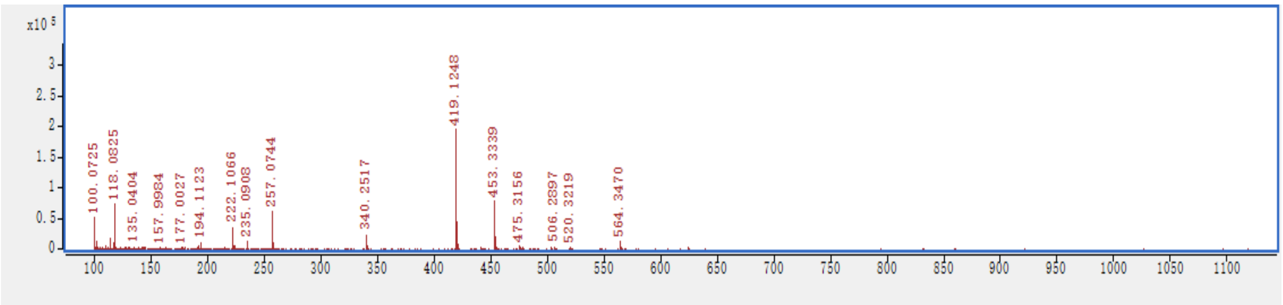

Figure S39. MS spectrum of peak 19

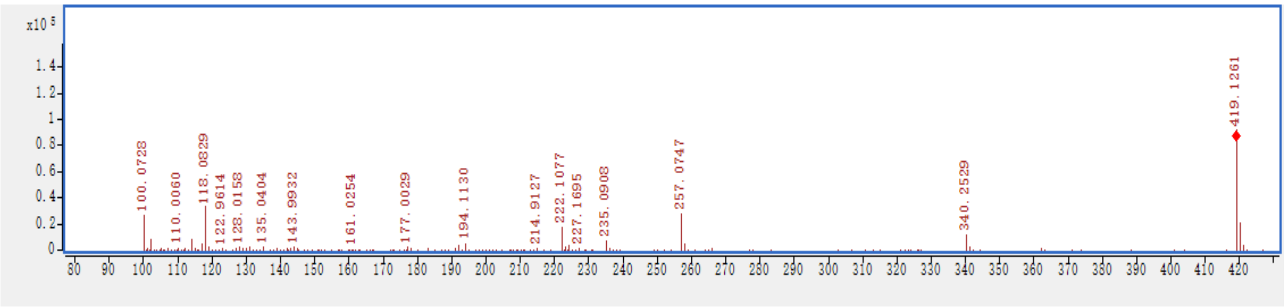

Figure S40. MS/MS spectrum of peak 19

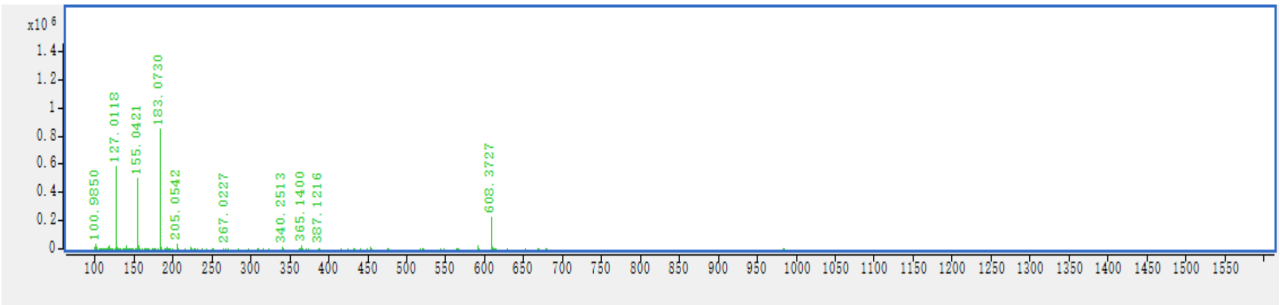

Figure S41. MS spectrum of peak 20

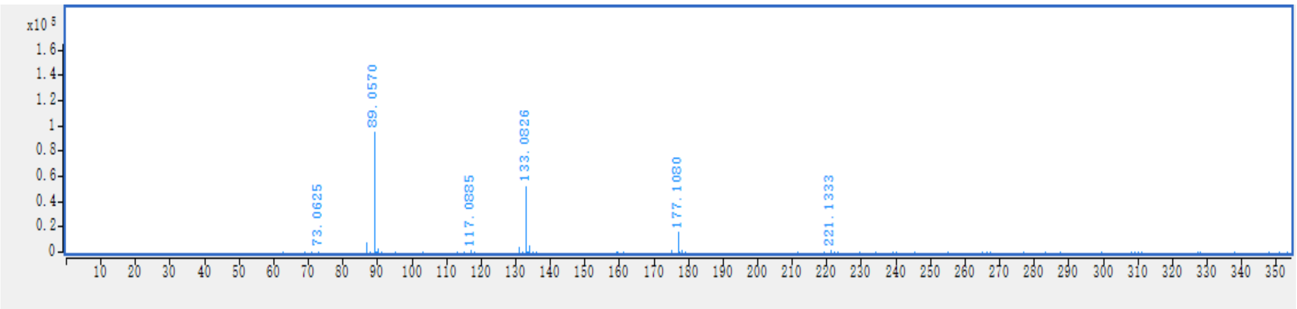

Figure S42. MS/MS spectrum of peak 20

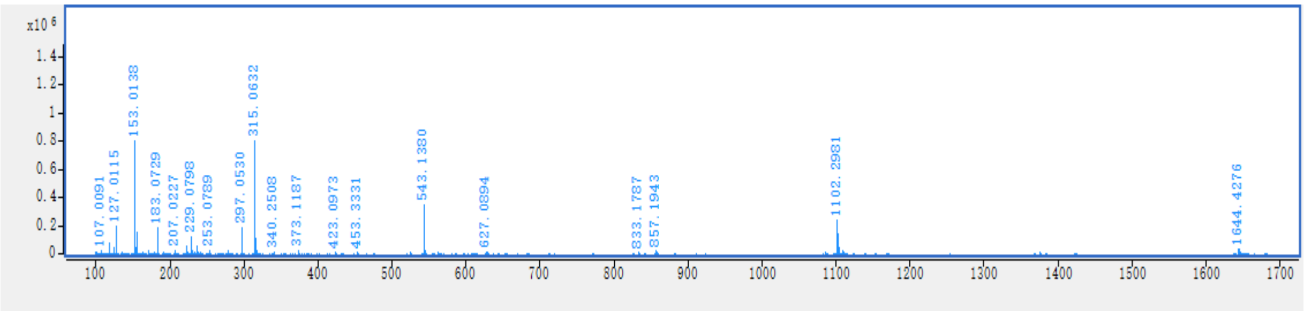

Figure S43. MS spectrum of peak 21

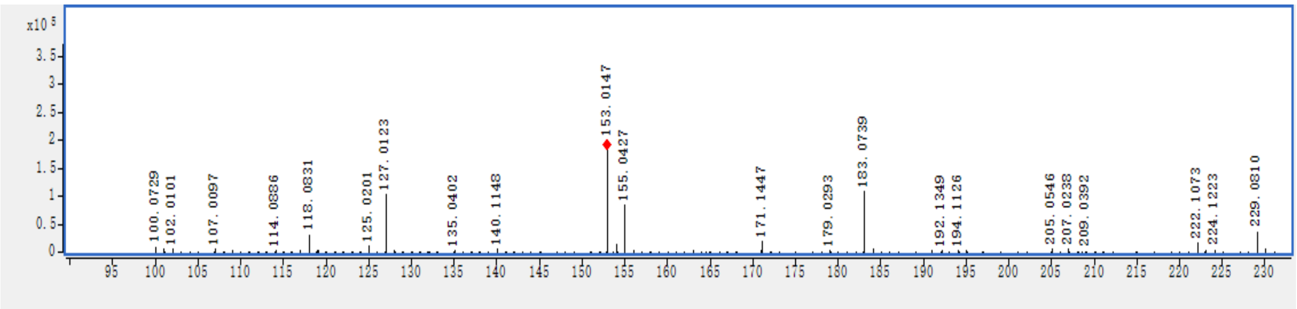

Figure S44. MS/MS spectrum of peak 21

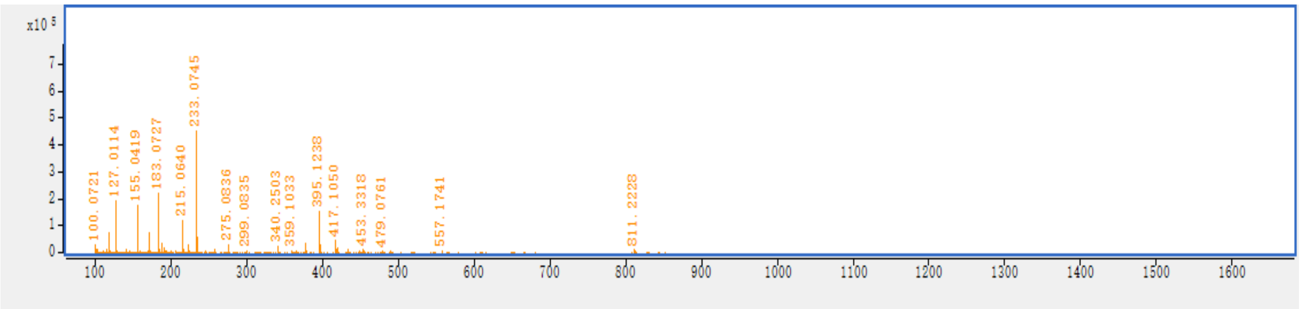

Figure S45. MS spectrum of peak 22

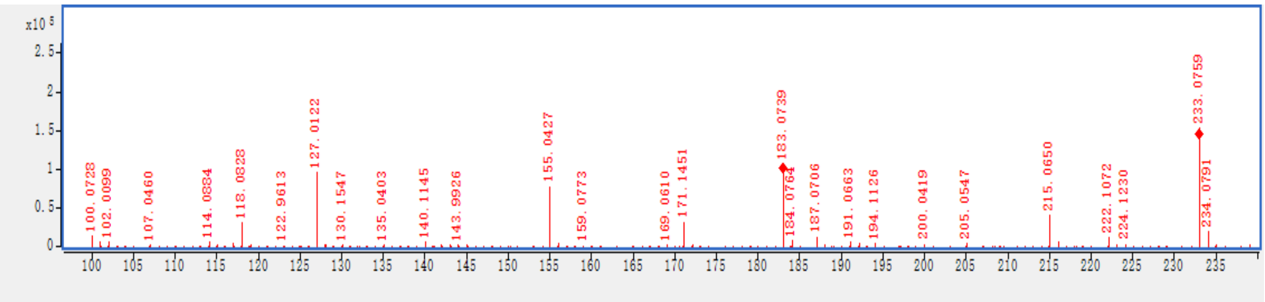

Figure S46. MS/MS spectrum of peak 22

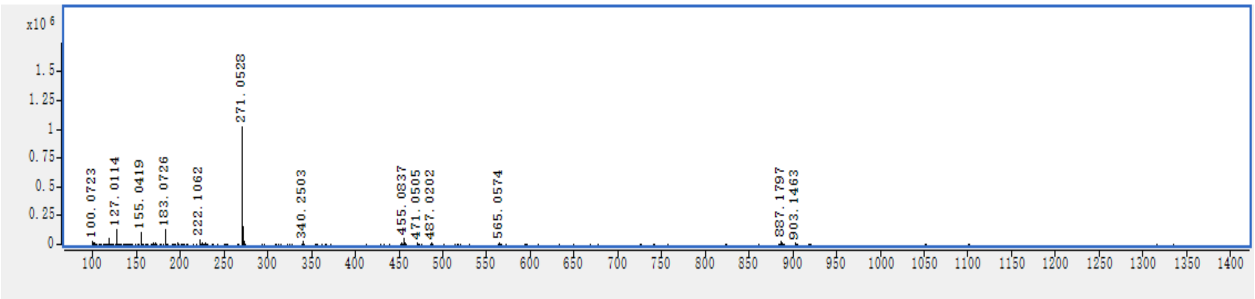

Figure S47. MS spectrum of peak 23

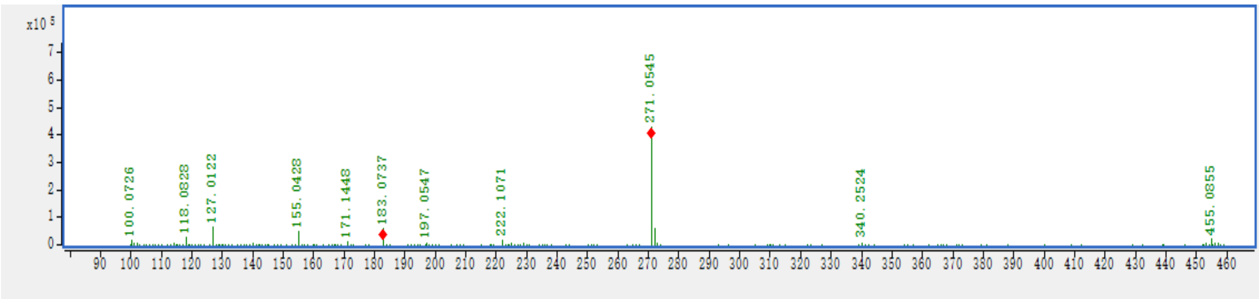

Figure S48. MS/MS spectrum of peak 23

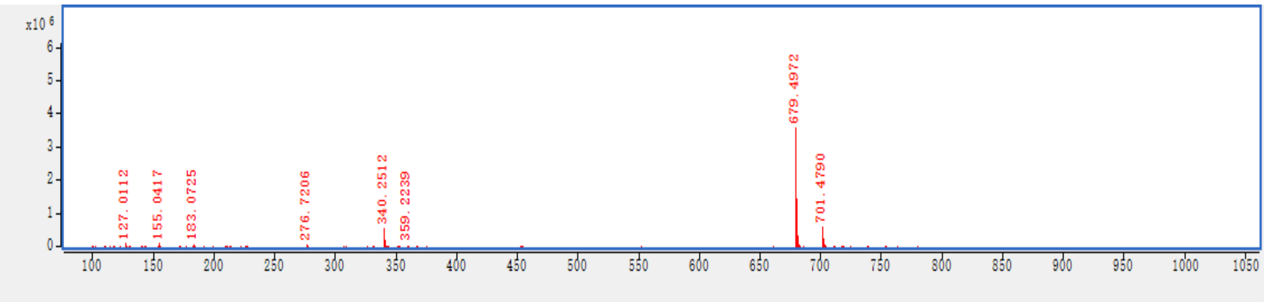

Figure S49. MS spectrum of peak 24

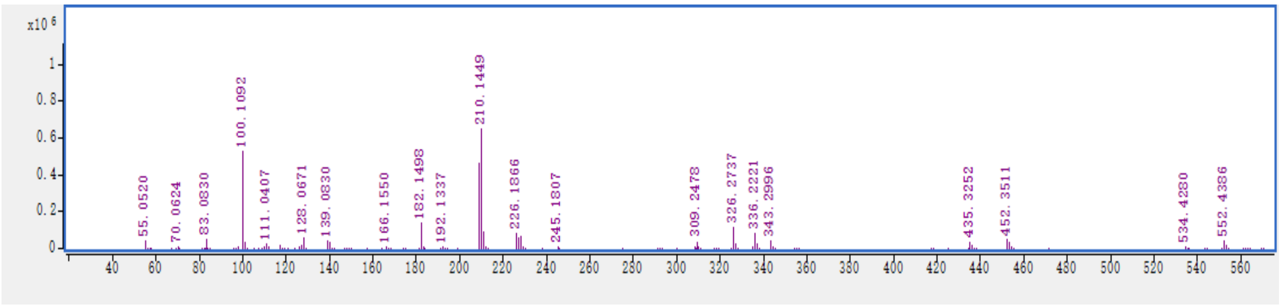

Figure S50. MS/MS spectrum of peak 24

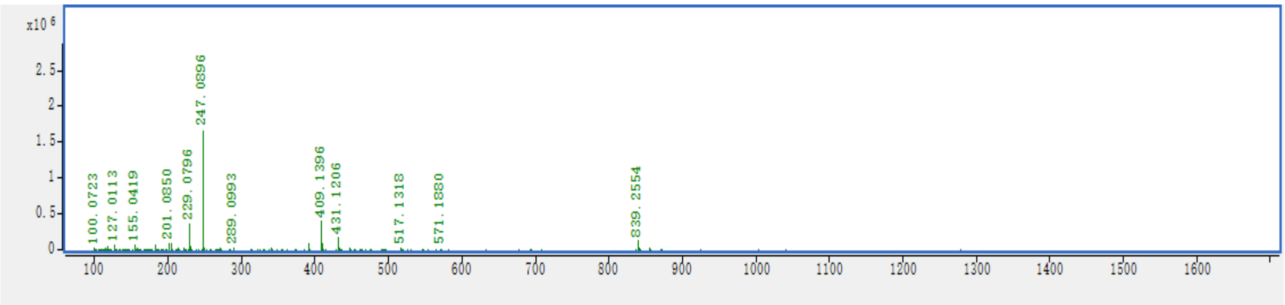

Figure S51. MS spectrum of peak 25

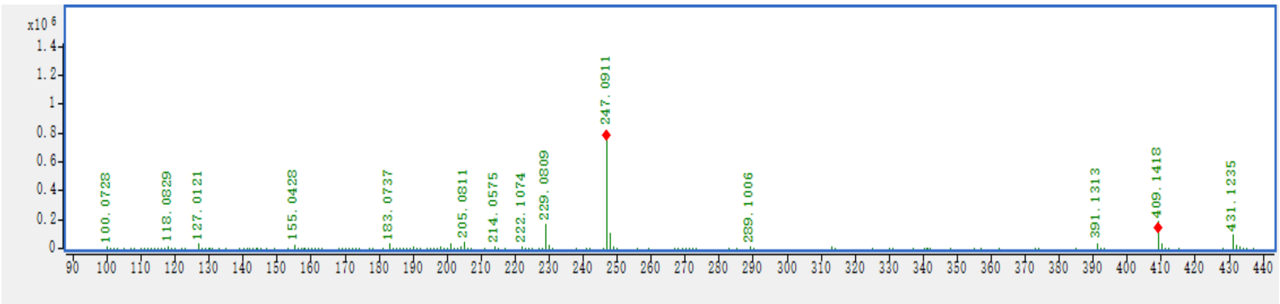

Figure S52. MS/MS spectrum of peak 25

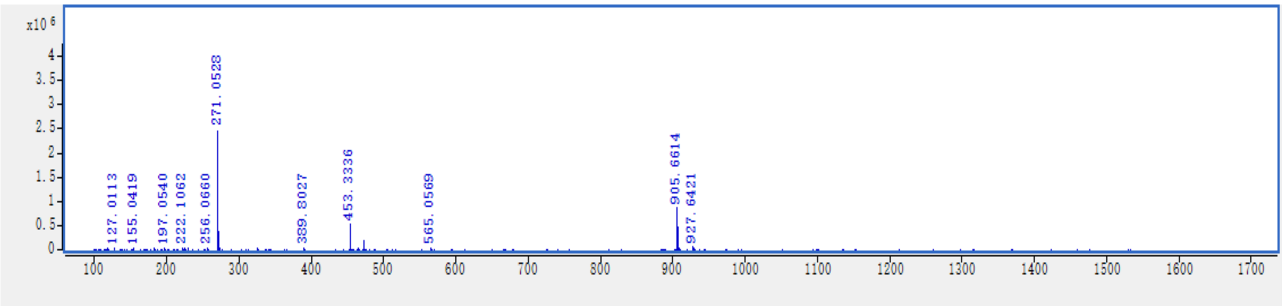

Figure S53. MS spectrum of peak 26

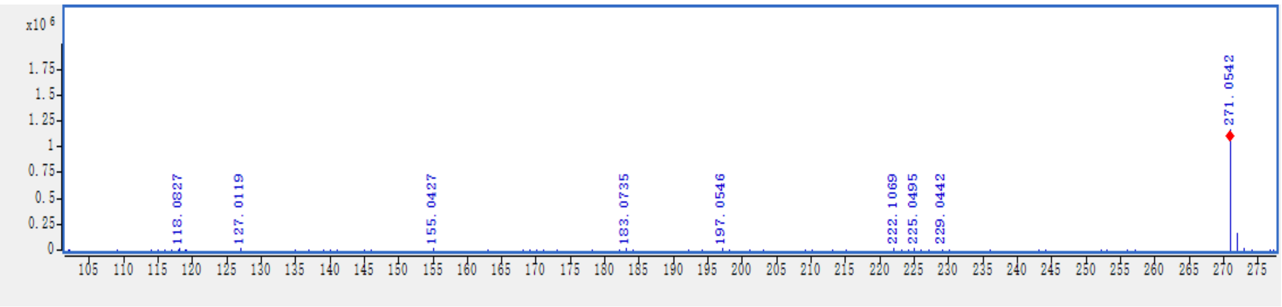

Figure S54. MS/MS spectrum of peak 26

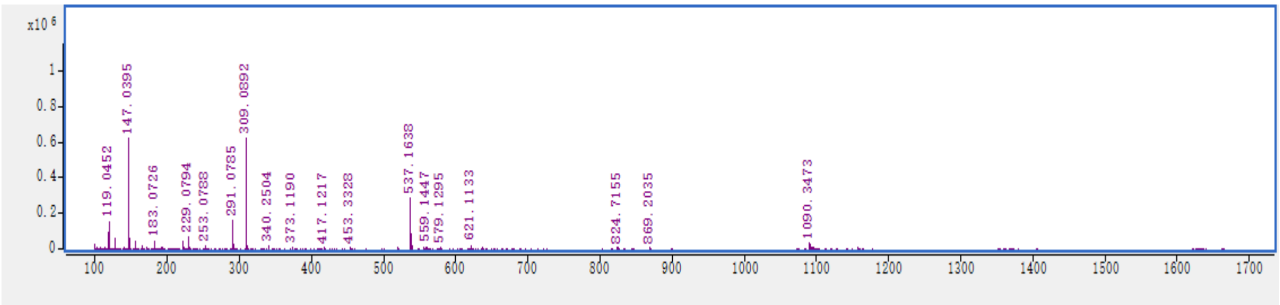

Figure S55. MS spectrum of peak 27

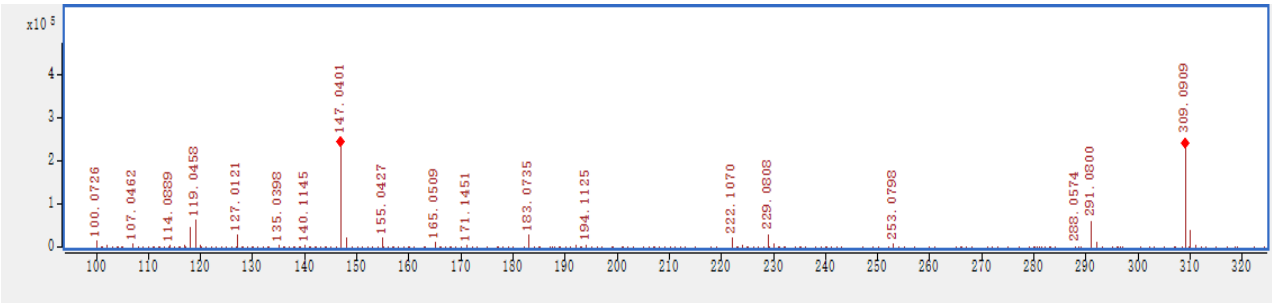

Figure S56. MS/MS spectrum of peak 27

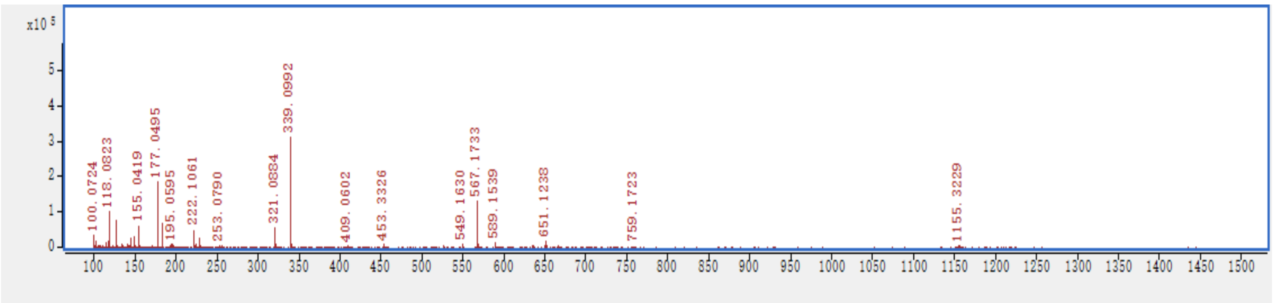

Figure S57. MS spectrum of peak 28

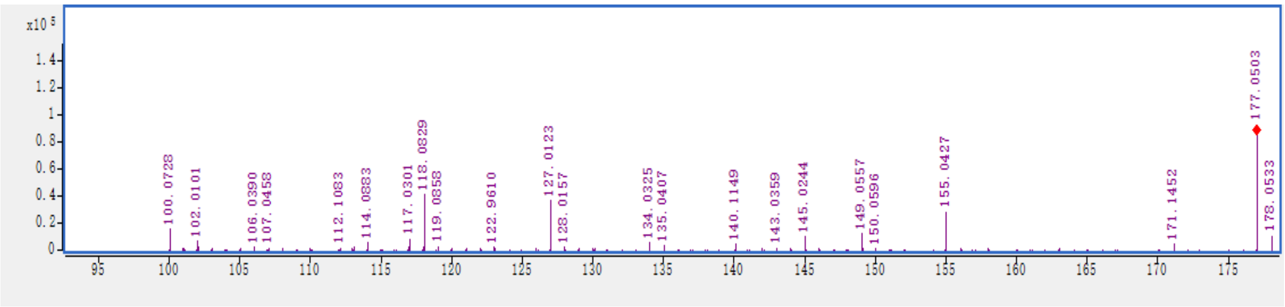

Figure S58. MS/MS spectrum of peak 28

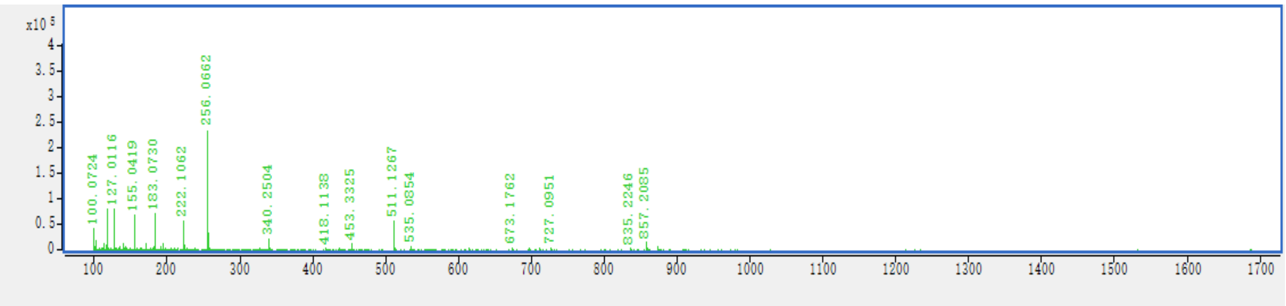

Figure S59. MS spectrum of peak 29

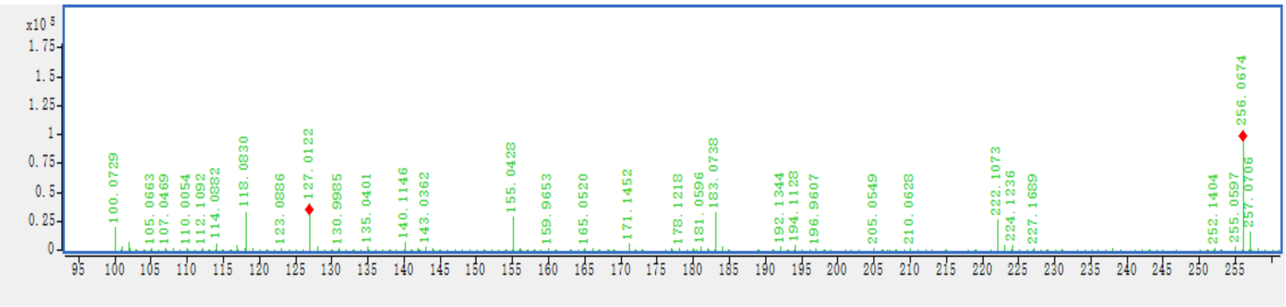

Figure S60. MS/MS spectrum of peak 29

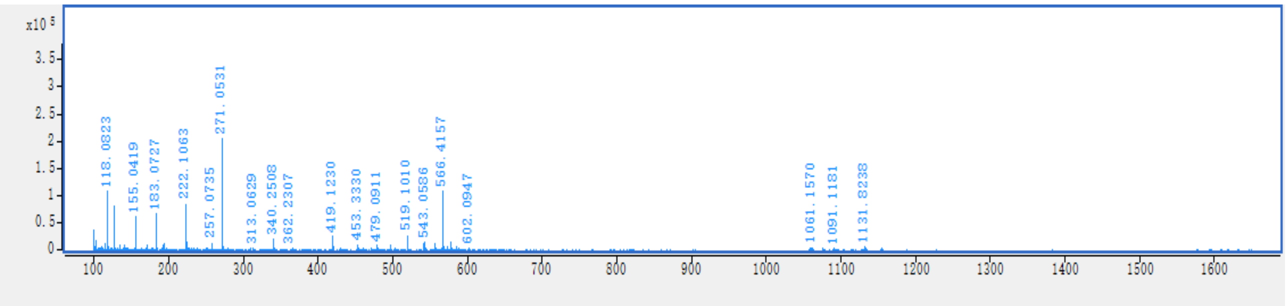

Figure S61. MS spectrum of peak 30

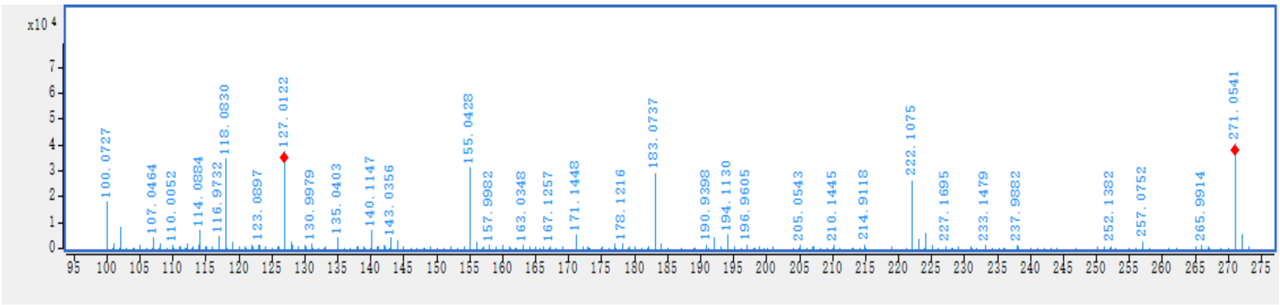

Figure S62. MS/MS spectrum of peak 30

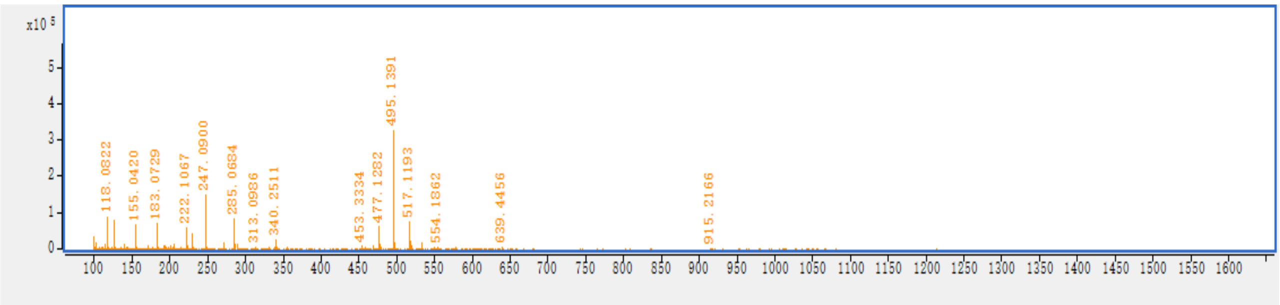

Figure S63. MS spectrum of peak 31

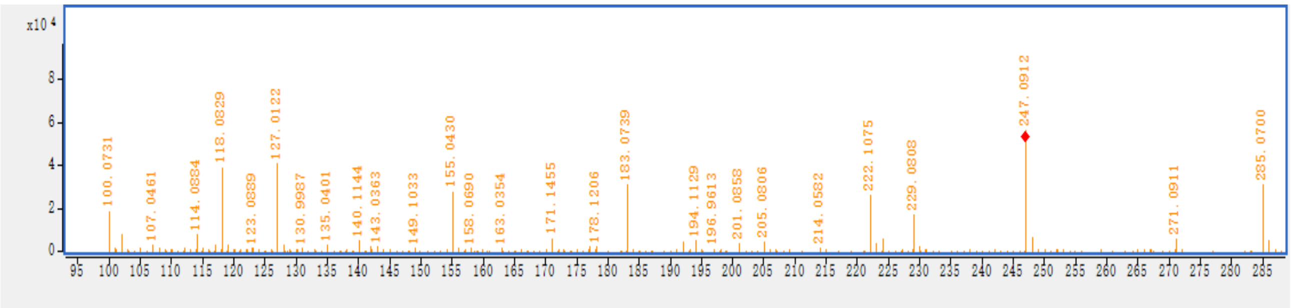

Figure S64. MS/MS spectrum of peak 31

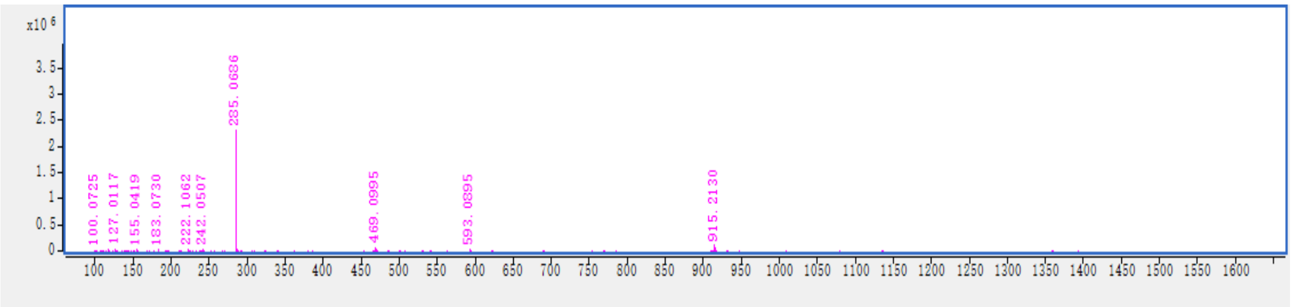

Figure S65. MS spectrum of peak 32

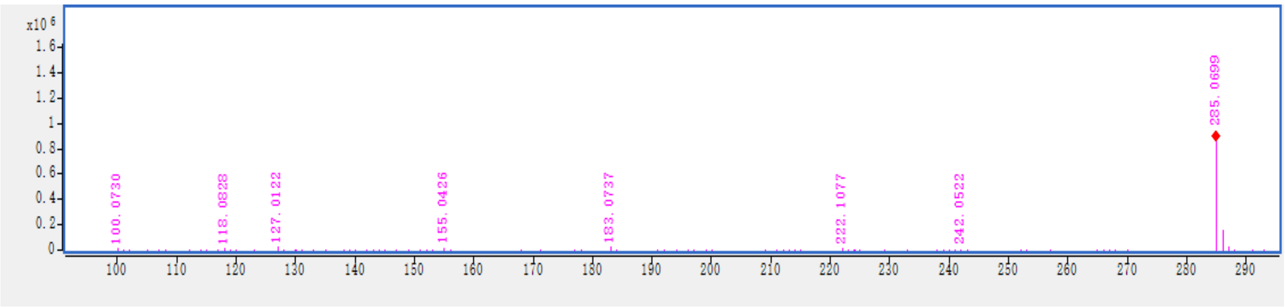

Figure S66. MS/MS spectrum of peak 32

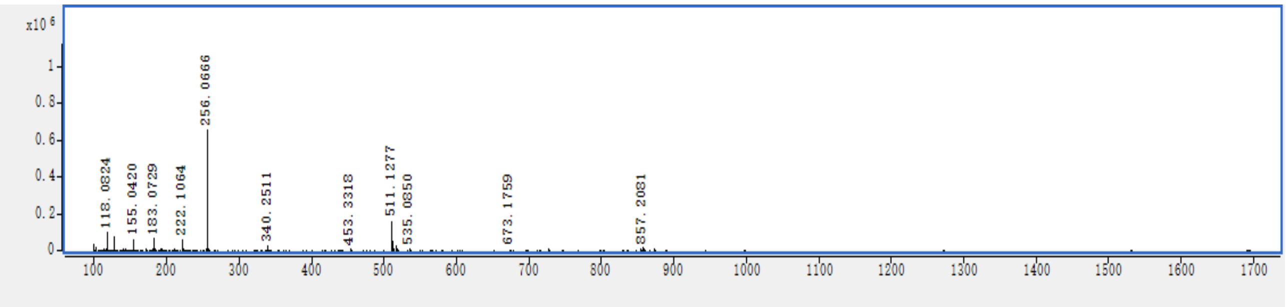

Figure S67. MS spectrum of peak 33

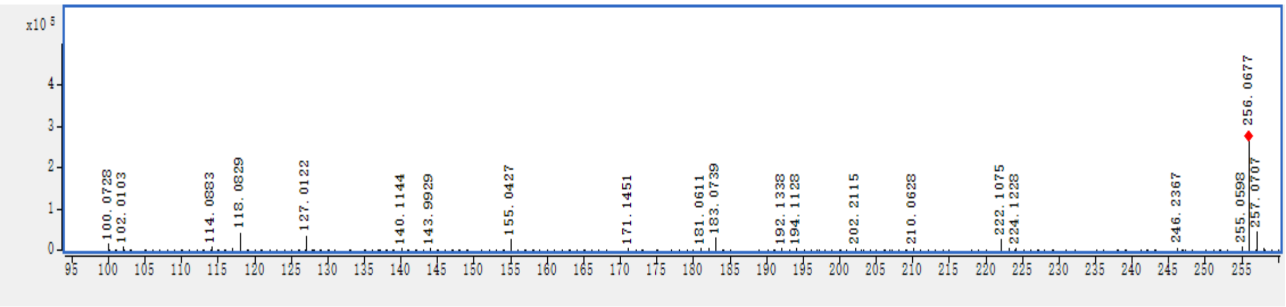

Figure S68. MS/MS spectrum of peak 33

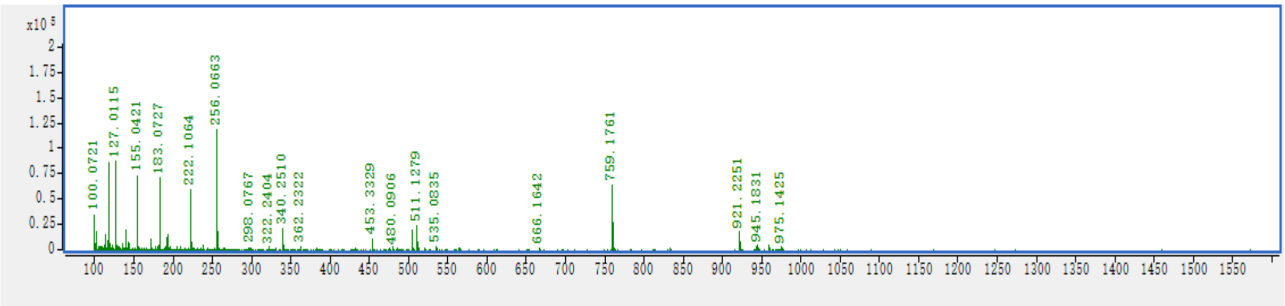

Figure S69. MS spectrum of peak 34

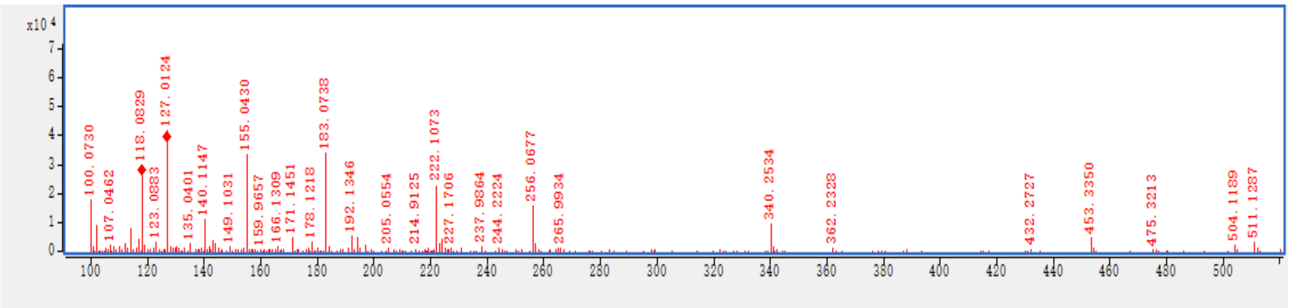

Figure S70. MS/MS spectrum of peak 34

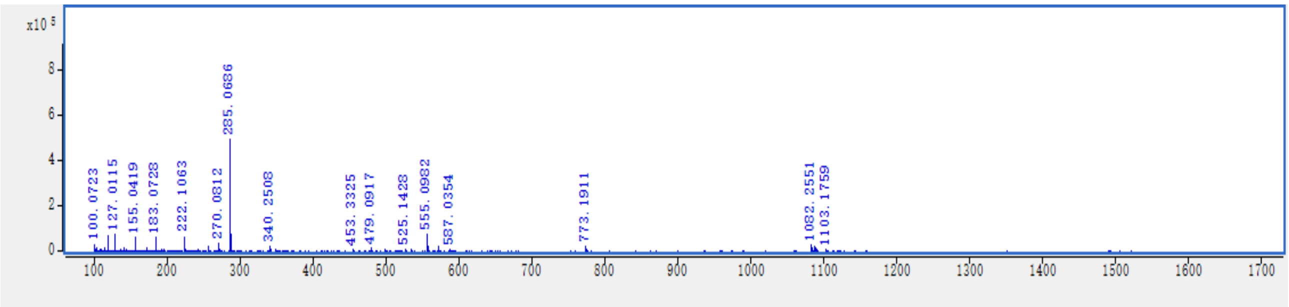

Figure S71. MS spectrum of peak 35

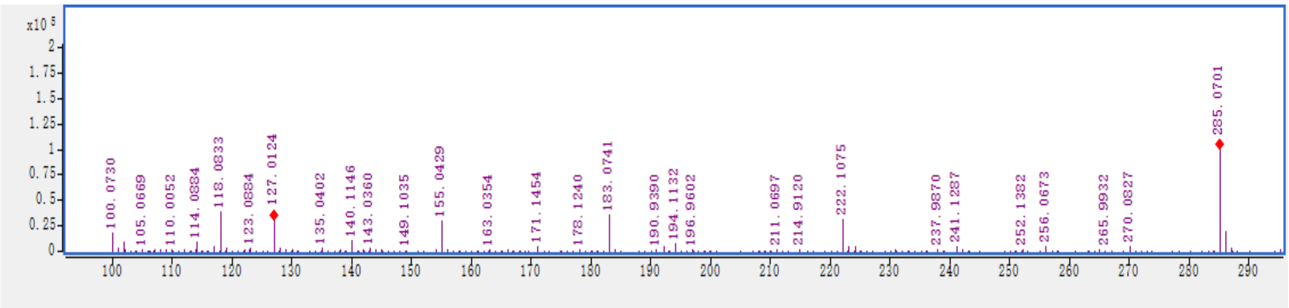

Figure S72. MS/MS spectrum of peak 35

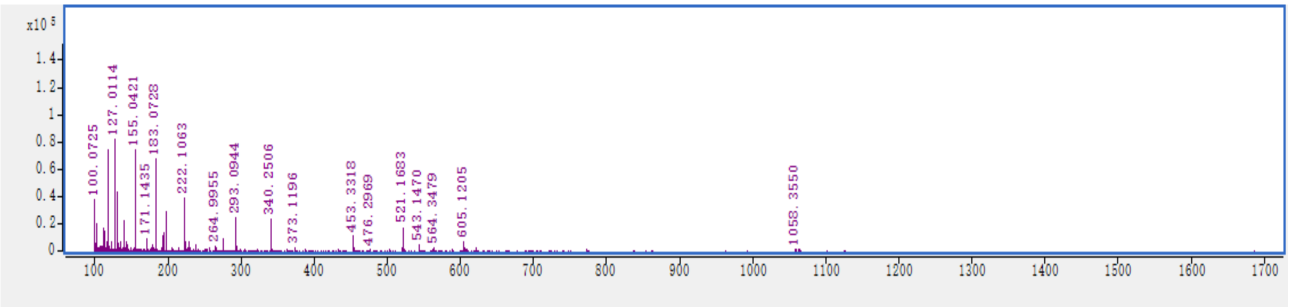

Figure S73. MS spectrum of peak 36

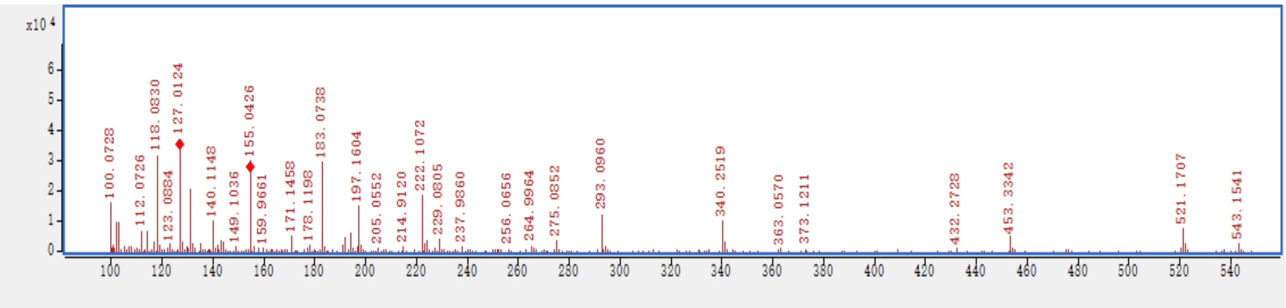

Figure S74. MS/MS spectrum of peak 36

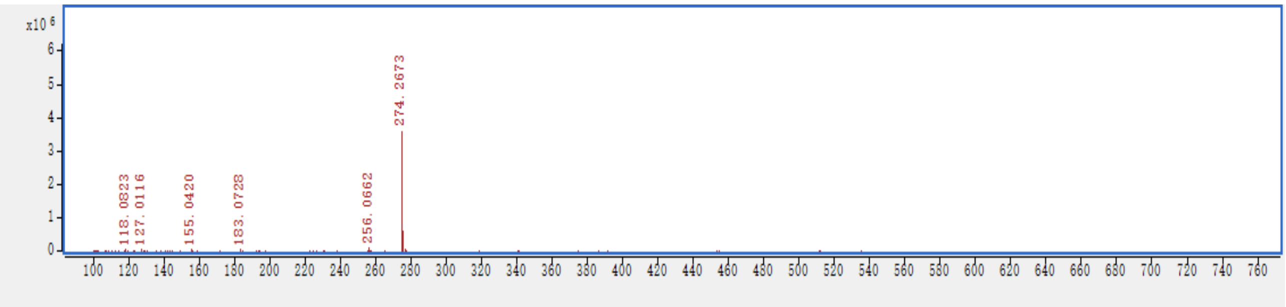

Figure S75. MS spectrum of peak 37

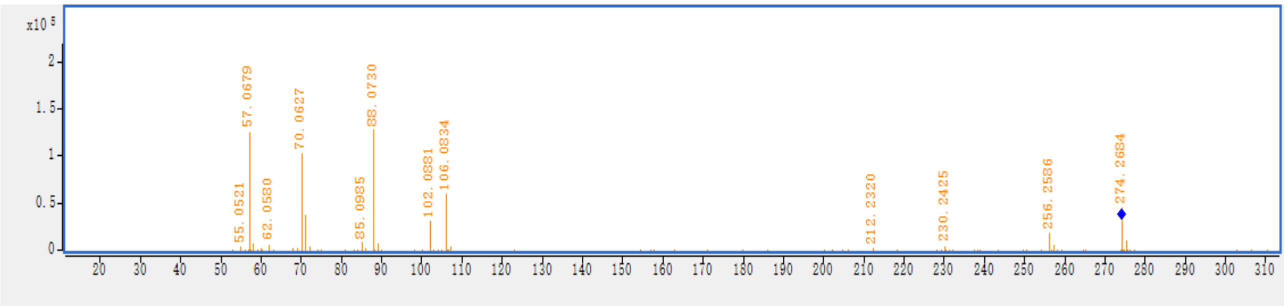

Figure S76. MS/MS spectrum of peak 37

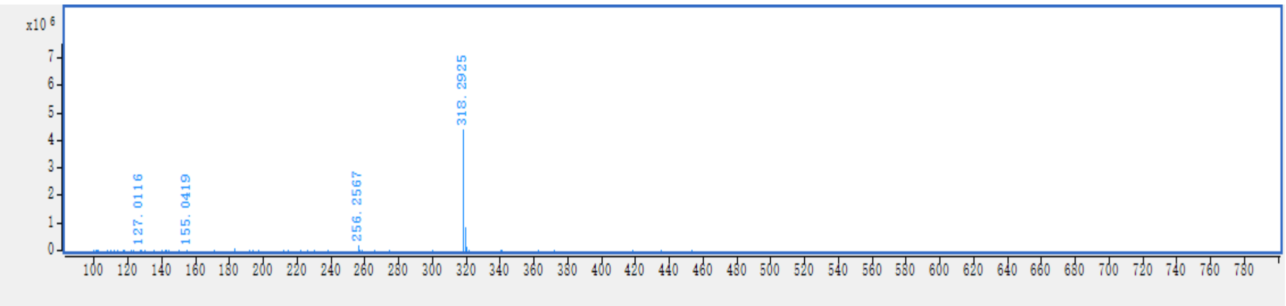

Figure S77. MS spectrum of peak 38

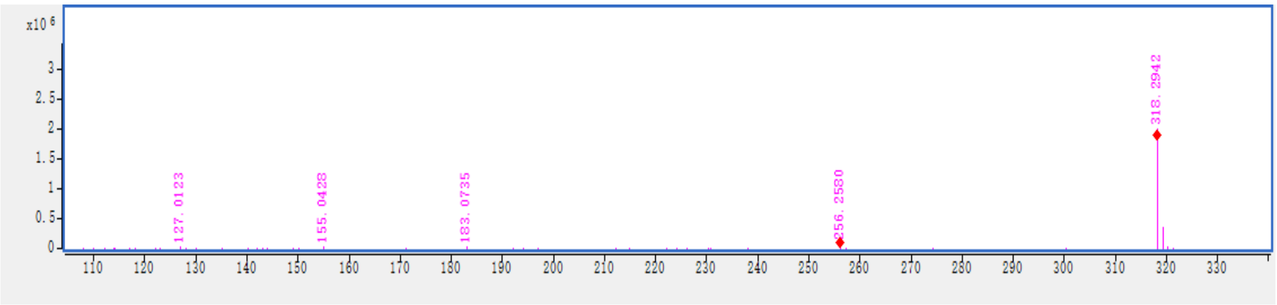

Figure S78. MS/MS spectrum of peak 38

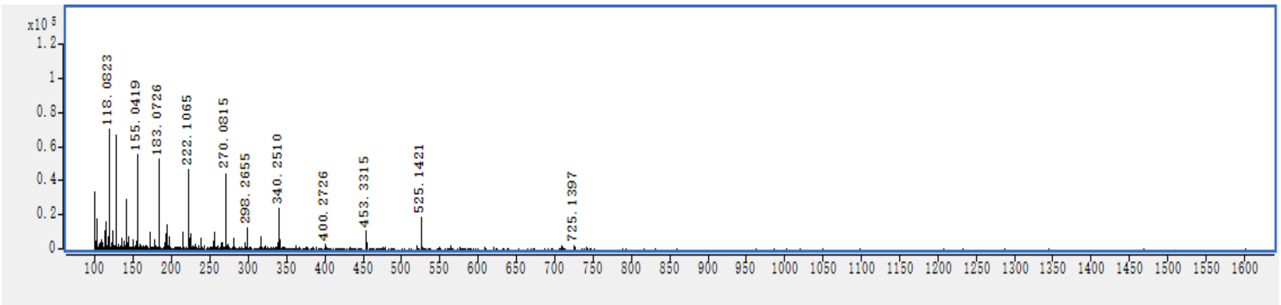

Figure S79. MS spectrum of peak 39

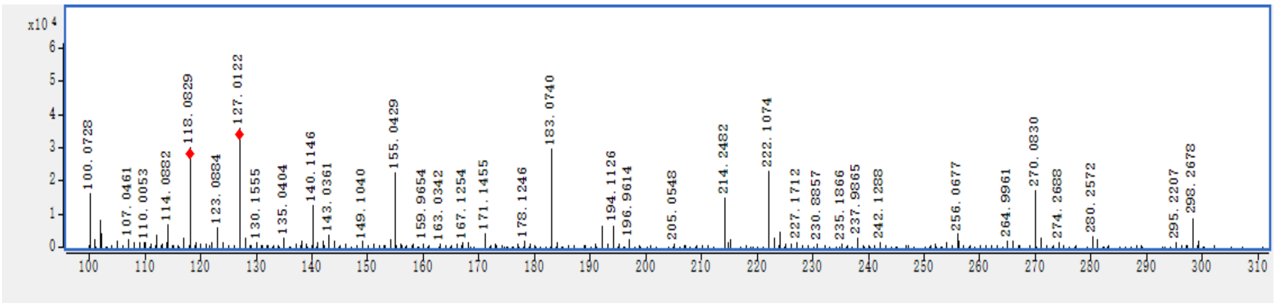

Figure S80. MS/MS spectrum of peak 39

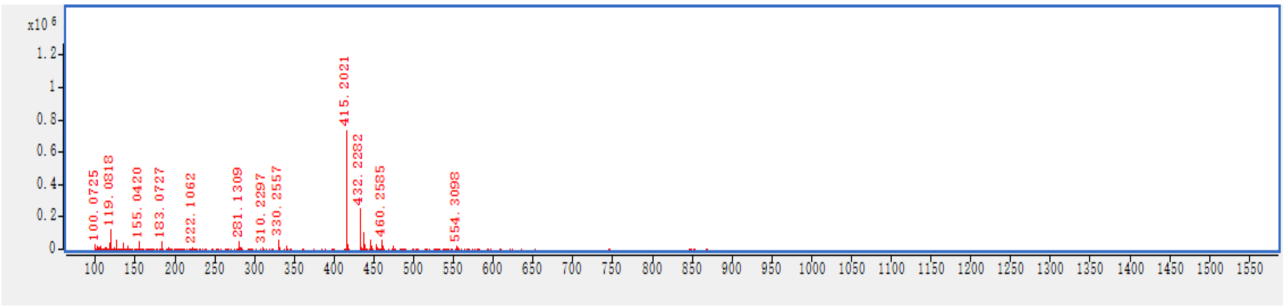

Figure S81. MS spectrum of peak 40

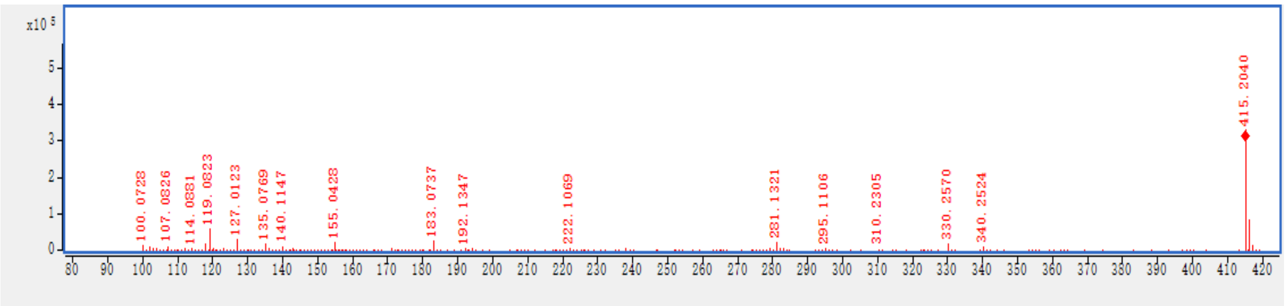

Figure S82. MS/MS spectrum of peak 40

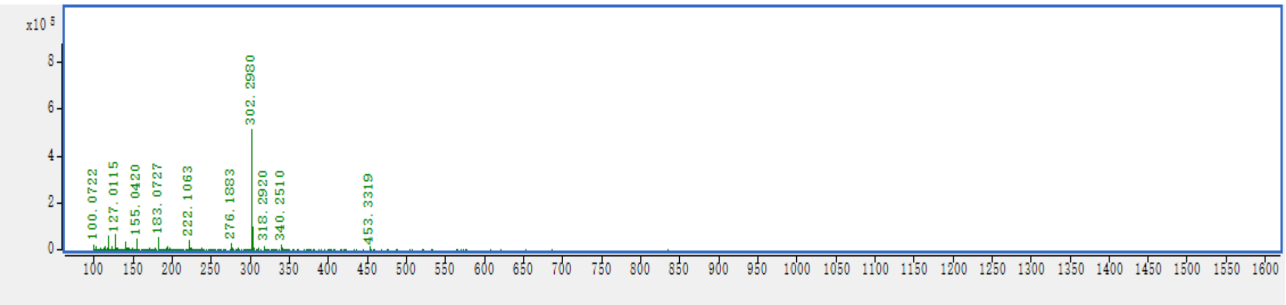

Figure S83. MS spectrum of peak 41

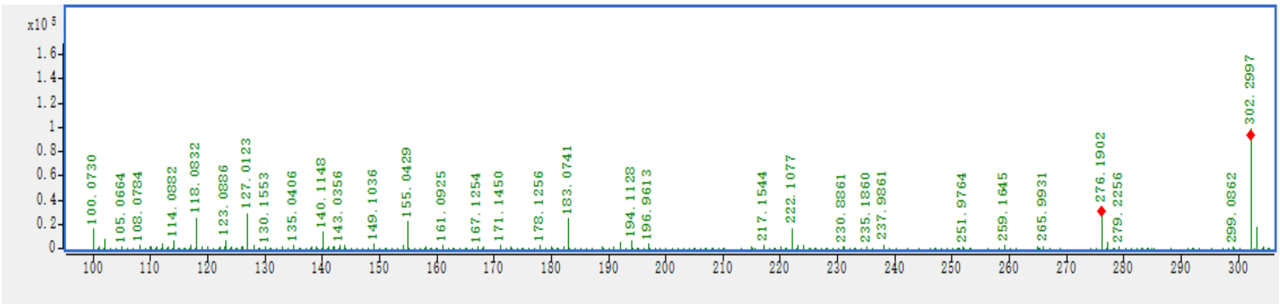

Figure S84. MS/MS spectrum of peak 41

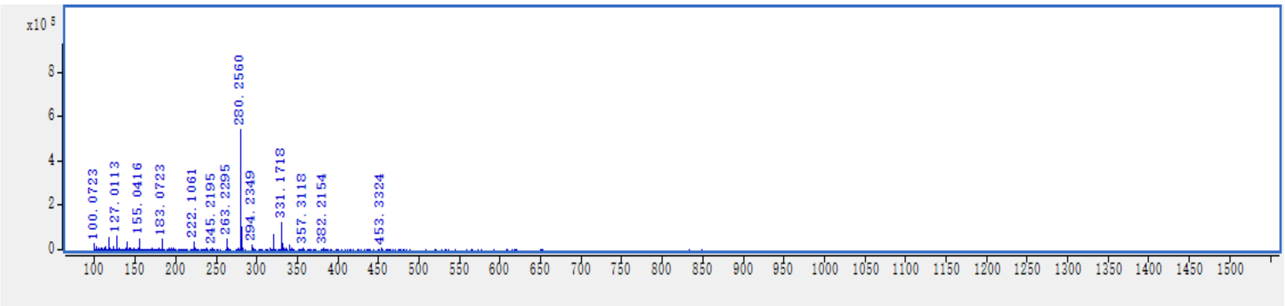

Figure S85. MS spectrum of peak 42

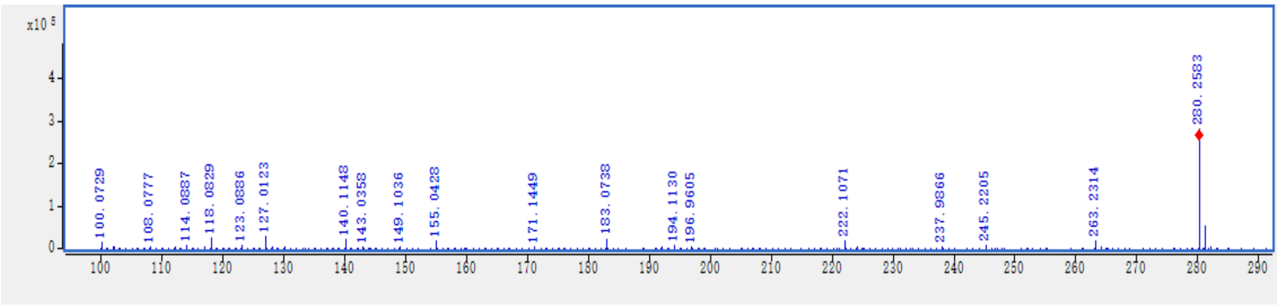

Figure S86. MS/MS spectrum of peak 42

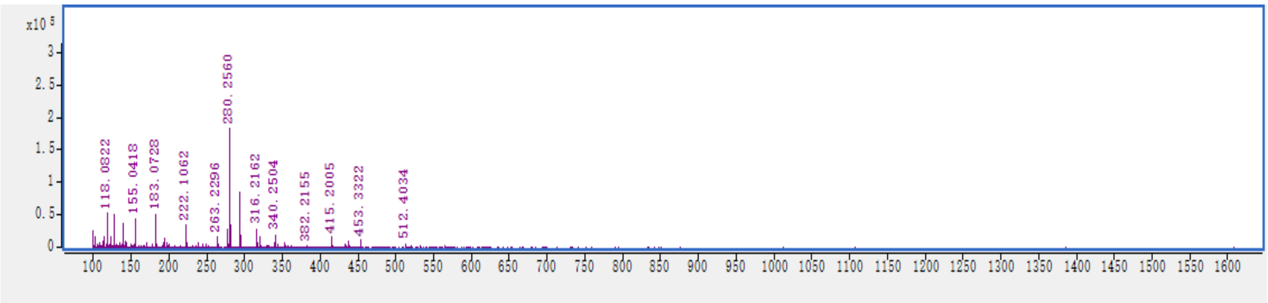

Figure S87. MS spectrum of peak 43

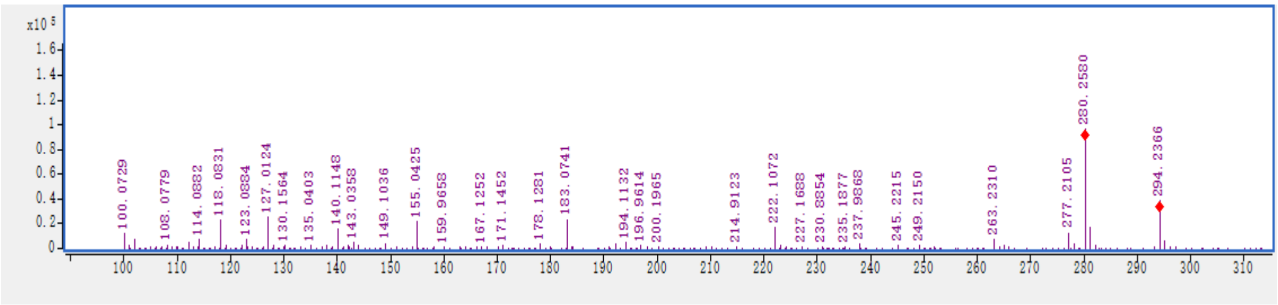

Figure S88. MS/MS spectrum of peak 43

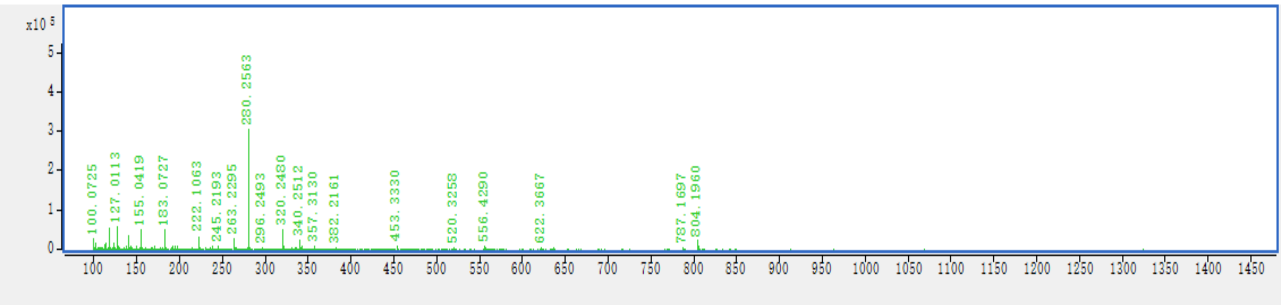

Figure S89. MS spectrum of peak 44

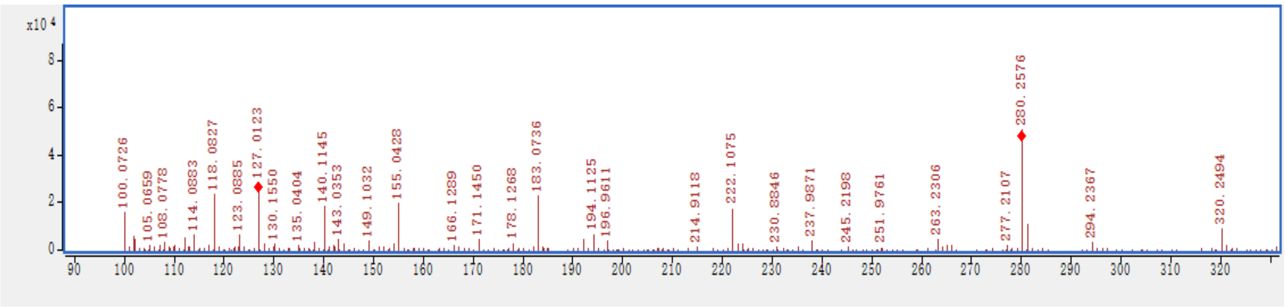

Figure S90. MS/MS spectrum of peak 44

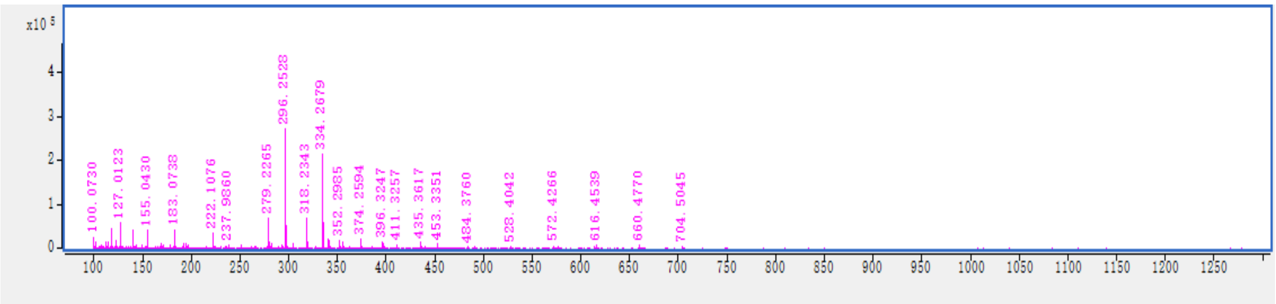

Figure S91. MS spectrum of peak 45

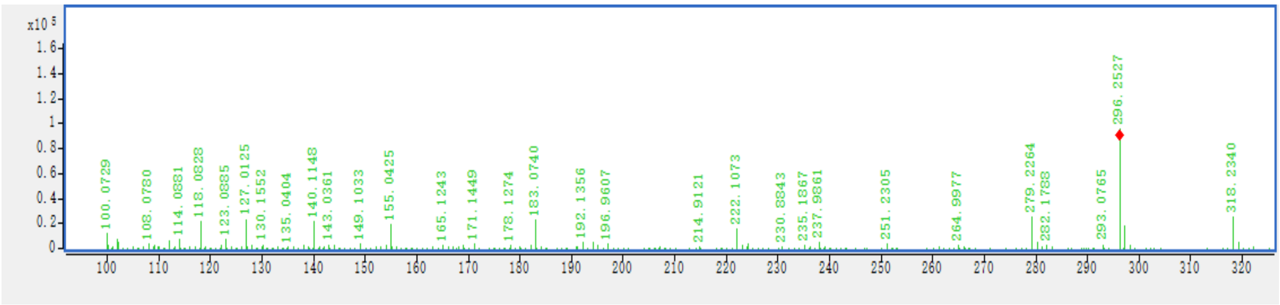

Figure S92. MS/MS spectrum of peak 45

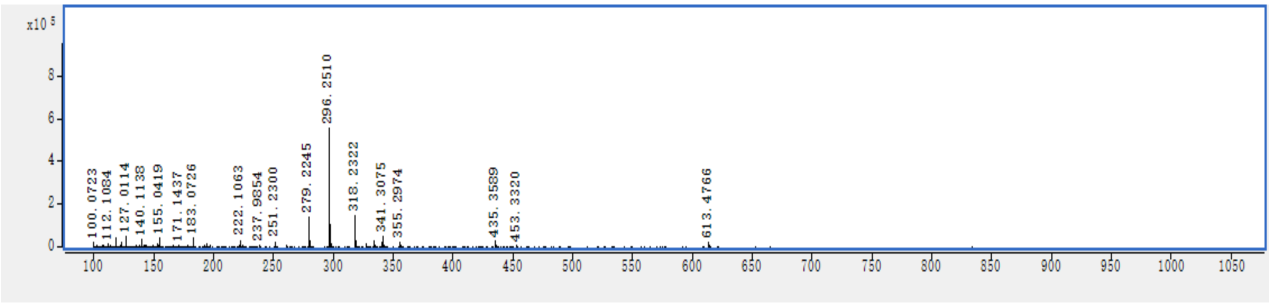

Figure S93. MS spectrum of peak 46

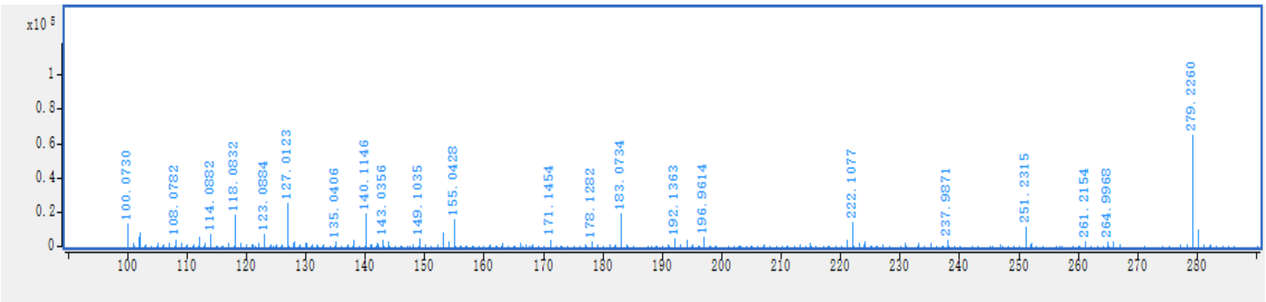

Figure S94. MS/MS spectrum of peak 46

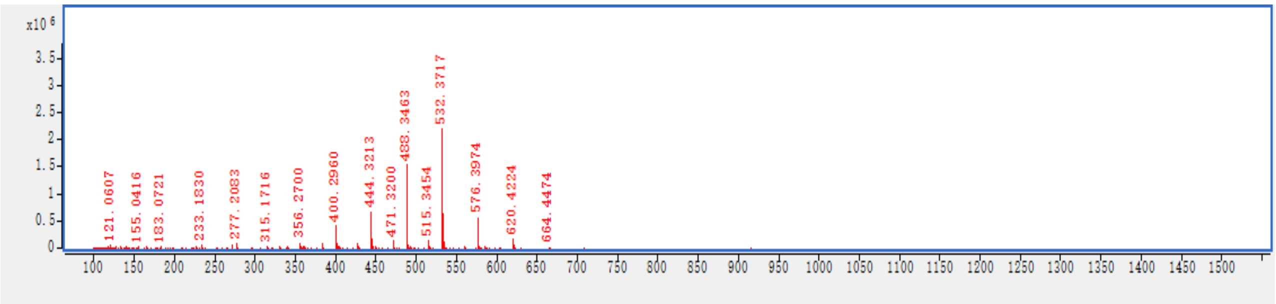

Figure S95. MS spectrum of peak 47

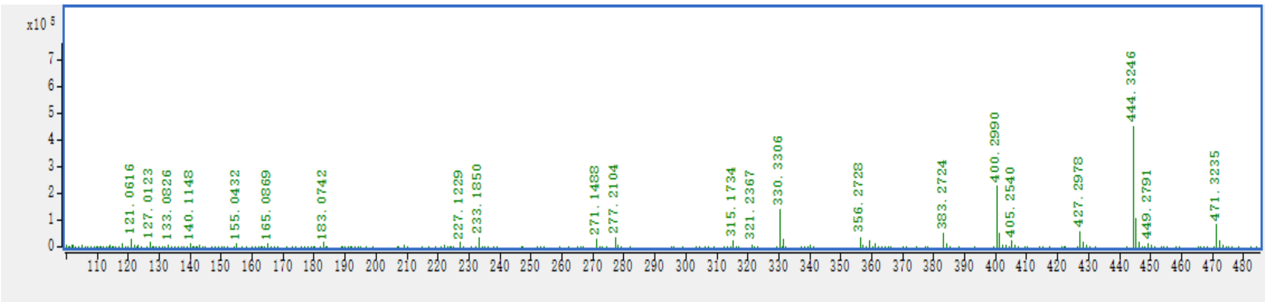

Figure S96. MS/MS spectrum of peak 47

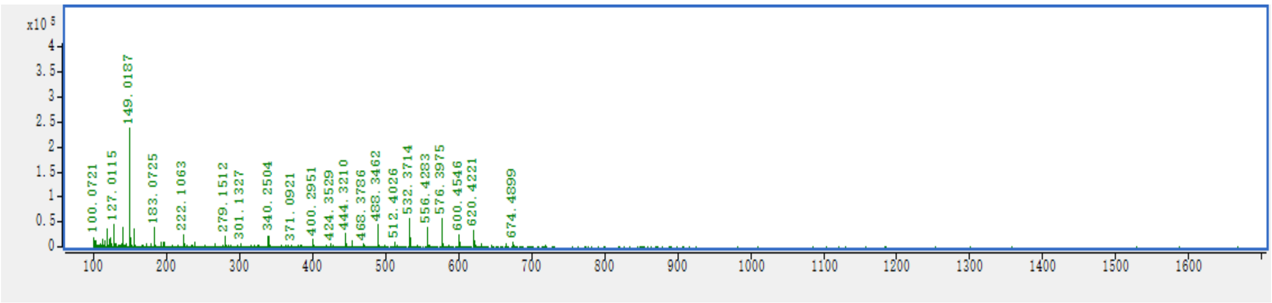

Figure S97. MS spectrum of peak 48

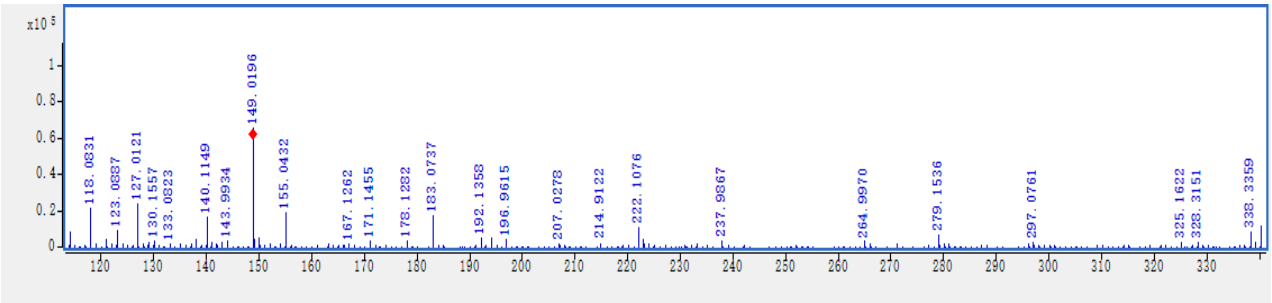

Figure S98. MS/MS spectrum of peak 48

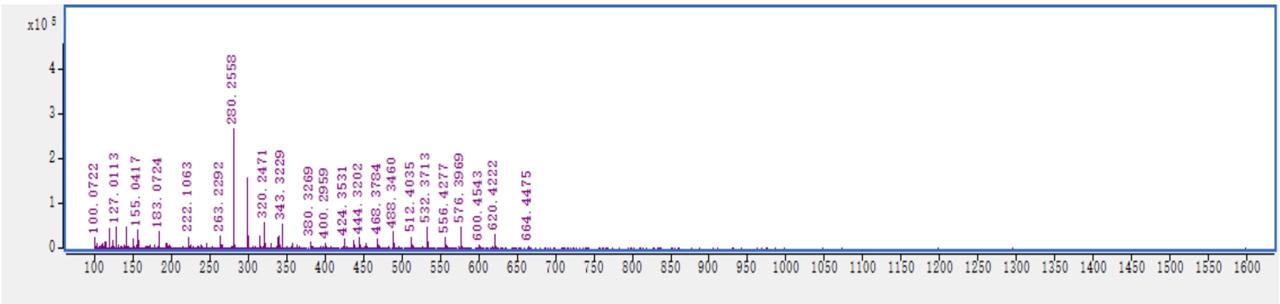

Figure S99. MS spectrum of peak 49

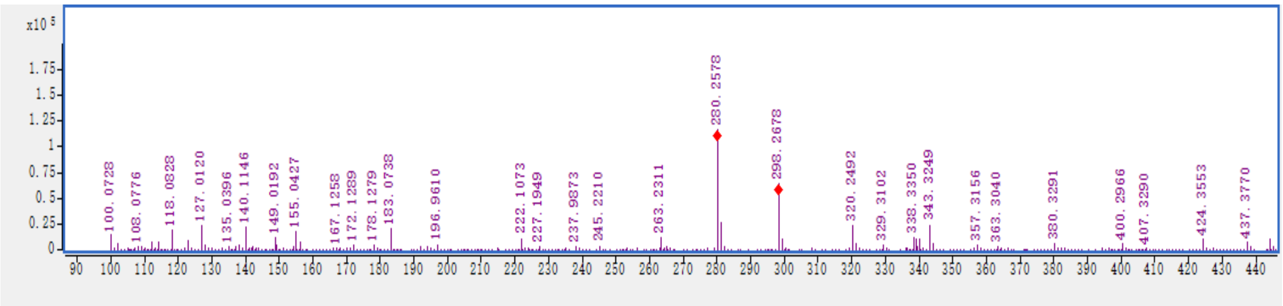

Figure S100. MS/MS spectrum of peak 49
